# Nickel‐Catalyzed, Reductive C(sp^3^)−Si Cross‐Coupling of α‐Cyano Alkyl Electrophiles and Chlorosilanes

**DOI:** 10.1002/anie.202107492

**Published:** 2021-07-16

**Authors:** Liangliang Zhang, Martin Oestreich

**Affiliations:** ^1^ Institut für Chemie Technische Universität Berlin Strasse des 17. Juni 115 10623 Berlin Germany

**Keywords:** cross-coupling, nickel, silicon, synthetic methods, zinc

## Abstract

A nickel/zinc‐catalyzed cross‐electrophile coupling of alkyl electrophiles activated by an α‐cyano group and chlorosilanes is reported. Elemental zinc is the stoichiometric reductant in this reductive coupling process. By this, a C(sp^3^)−Si bond can be formed starting from two electrophilic reactants whereas previous methods rely on the combination of carbon nucleophiles and silicon electrophiles or vice versa.

## Graphical abstract

An activated alkyl electrophile and a silicon electrophile can be reductively cross‐coupled under nickel catalysis with elemental zinc as the stoichiometric reductant. This cross‐electrophile coupling enables for the first time the formation of a C(sp^3^)−Si bond from alkyl electrophiles with an α‐cyano group and chlorosilanes (see Scheme; dtbpy=4,4′‐di‐*tert*‐butyl‐2,2′‐dipyridyl).

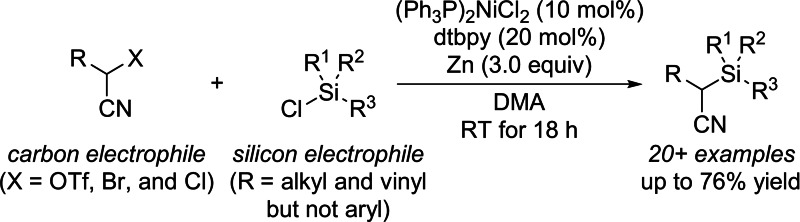

Transition‐metal‐catalyzed C(sp^3^)−Si bond formation^[1]^ by cross‐coupling of either carbon nucleophile/silicon electrophile^[2]^ or carbon electrophile/silicon (pro)nucleophile[^3^, ^4^, ^5^] combinations has seen substantial progress in recent years but several challenges remain (Scheme 1, top). For example, asymmetric versions are currently limited to copper‐catalyzed, S_N_2‐type reactions of activated alkyl electrophiles with boron‐based silicon pronucleophiles^[4]^ or uncatalyzed displacements of unactivated alkyl electrophiles with metalated silicon reagents.^[6]^ Another open task is to combine carbon and silicon electrophiles in reductive coupling reactions not requiring any preceding metalation of either coupling partner. Last year, such a reductive process was described for C(sp^2^)−Si bond formation starting from vinyl and aryl triflates/halides by Shu and co‐workers (Scheme 1, bottom).[^7^, ^8^] Shu's procedures rely on nickel(II) bipyridine complexes as precatalysts and elemental manganese as the stoichiometric reductant. A requirement of this broadly applicable method is that the chlorosilane must be substituted with a vinyl group to enhance its coordination ability to the nickel catalyst. A related C(sp^3^)−Si bond‐forming reaction is not known to date. We disclose here a nickel‐catalyzed cross‐electrophile coupling^[9]^ of an activated alkyl electrophile[^4a^, ^10^] and various chlorosilanes (Scheme 1, bottom).

**Scheme 1:**
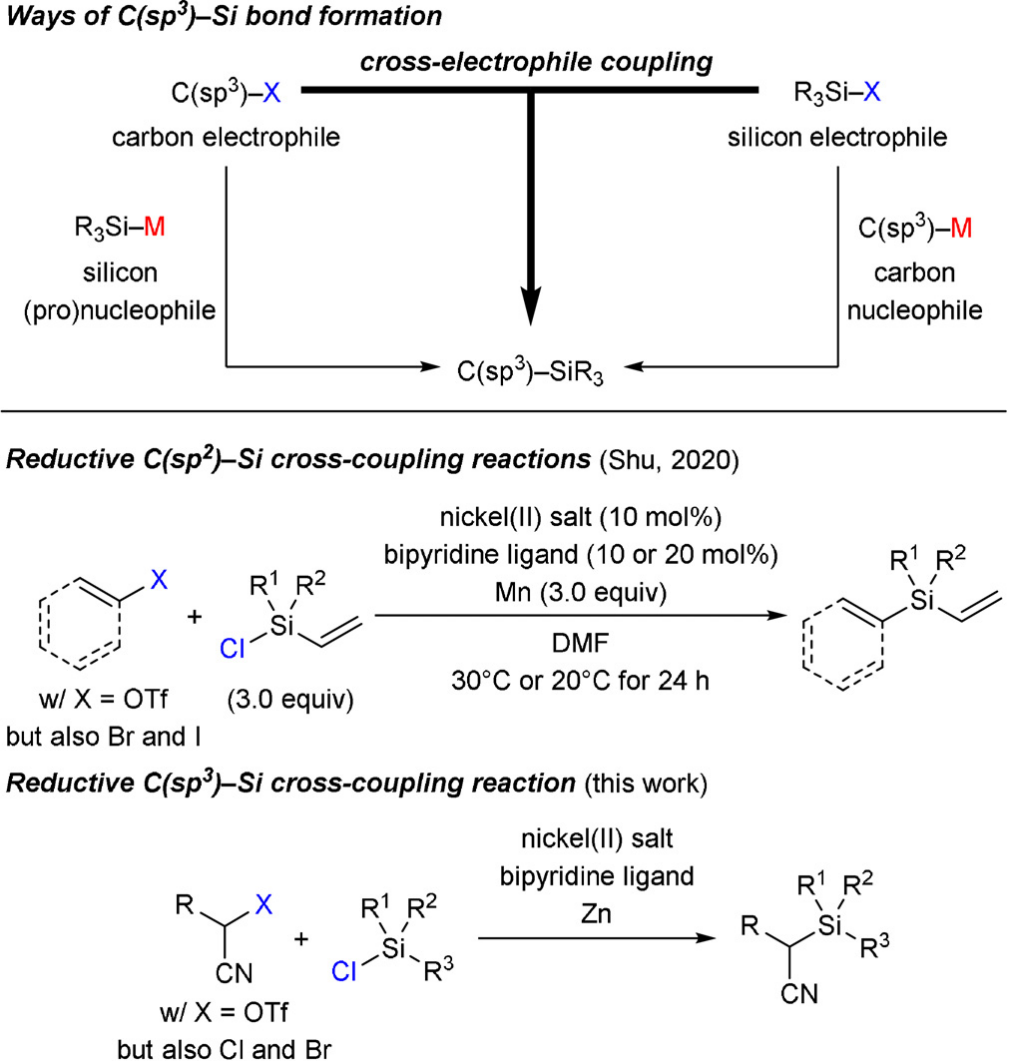
Methods of transition‐metal‐catalyzed C−Si bond formation. DMF=*N*,*N*‐dimethylformamide, Tf=trifluoromethanesulfonyl.

We started with the reaction of α‐triflyloxy nitrile **1 a** and vinyl‐substituted chlorosilane **2 a** (Table 1). Extensive examination of the reaction parameters revealed that the combination of (Ph_3_P)_2_NiCl_2_/**L3** and elemental zinc in DMA is optimal (see Tables S1–S4 in the Supporting Information for details). The α‐silyl nitrile **3 aa** was obtained in 76 % isolated yield at room temperature (entry 1). Control experiments showed that the stoichiometric reductant zinc and the nickel catalyst are needed; with no additional ligand the yield was lower (entries 2–4). Other bipyridine ligands such as **L1** and **L2** as well as terpyridine **L4** did not give any improvement over **L3** (entries 5–7); the yield collapsed when using 1,10‐phenanthroline (**L5**; entry 8). Replacing zinc by manganese resulted in a lower yield (entry 9). Related α‐cyano alkyl electrophiles with chloride and bromide leaving groups afforded **3 aa** also in good yields (entries 10 and 11). The reactions were routinely run at room temperature, and no significant effect was seen at higher or lower reaction temperature (entries 12 and 13). Of note, the attempted reductive coupling of unactivated alkyl electrophiles such as 3‐phenylpropyl trifluoromethanesulfonate and cyclohexyl bromide did not lead to the formation of the desired product (not shown).

**Table 1:** Selected examples of the optimization.^[a]^ 
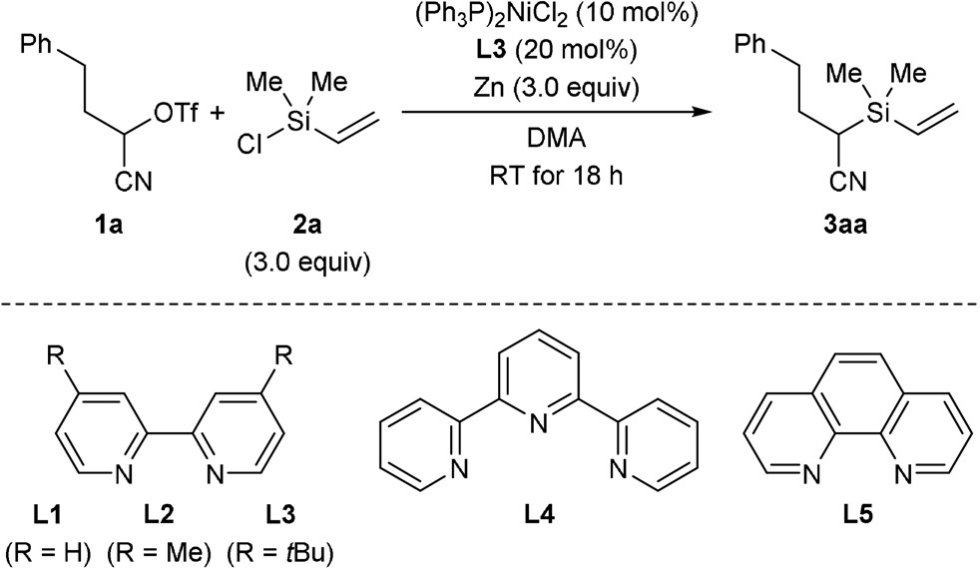

Entry	Variation	Yield [%]^[b]^
1	None	90 (76)^[c]^
2	w/o Zn	0
3	w/o (Ph_3_P)_2_NiCl_2_	0
4	w/o **L3**	70
5	**L1** instead of **L3**	85
6	**L2** instead of **L3**	80
7	**L4** instead of **L3**	76
8	**L5** instead of **L3**	5
9	Mn instead of Zn	54
10	Cl instead of OTf	80
11	Br instead of OTf	75
12	0 °C instead of RT	83
13	40 °C instead of RT	81

[a] All reactions were performed on a 0.20 mmol scale. [b] Determined by GLC analysis with tetracosane as an internal standard. [c] Isolated yield after purification by flash chromatography on silica gel. DMA=*N*,*N*‐dimethylacetamide.

With the optimized setup in hand, we tested other chlorosilanes (Scheme 2). Consistent with Shu's results,^[7]^ trivinylchlorosilane (**2 b**) brought about an isolated yield in the range of that obtained with **2 a**. However, trialkylchlorosilanes **2 c** and **2 d** devoid of the nickel‐coordinating vinyl group also participated in this reductive cross‐coupling; **3 ac** and **3 ad** did form in 33 % and 40 % yield, respectively. This stands in contrast to Shu's report^[7]^ and is remarkable in the sense that especially Me_3_SiCl (**2 d**) is an often‐used additive in nickel‐catalyzed, reductive cross‐coupling reactions but without any C‐Si coupling products being observed.^[11]^ Chlorosilanes bearing a phenyl ring or a *tert*‐butyl group on the silicon atom did not lead to the α‐cyano silane but instead converted into the corresponding disilane and disiloxane (not shown).

**Scheme 2:**
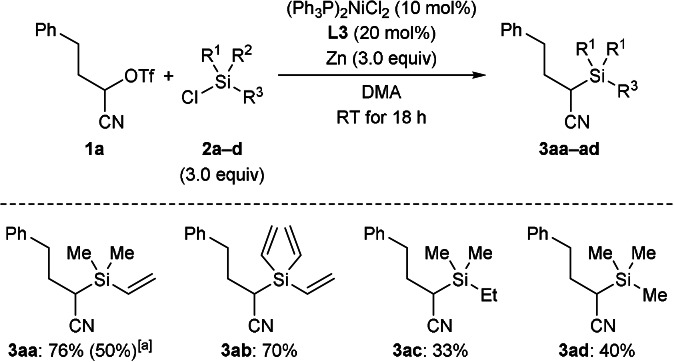
Scope I: Variation of the chlorosilane. All reactions were performed on a 0.20 mmol scale with the isolated yield determined after flash chromatography on silica gel. [a] Value in parentheses for reaction on a 1.0 mmol scale.

Proceeding with vinylchlorosilane **2 a** as the electrophilic silicon reactant, we subjected various α‐triflyloxy nitriles **1 b**–**r** to the general procedure (Scheme 3). Derivatives of model substrate **1 a** with halogenation at the aryl group reacted in good yields (**1 b**,**c**→**3 ba**,**ca**); no cross‐electrophile coupling of the aryl bromide in **1 c** to form a C(sp^2^)−Si bond was observed. Substrate **1 d** containing a furyl unit instead of the aryl group converted equally well into the desired product **3 da**. Substrate **1 e** with a longer alkyl tether than in **1 a**–**d** led to a similar result (**1 e**→**3 ea**). Further substrates with different kinds of functional groups such as a primary alkyl bromide as in **1 f**, various esters as in **1 g**–**i**, an ether as in **1 j**, and unsaturation as in **1 k**,**l** were tolerated in moderate to good yields. Conversely, a linear alkyl residue resulted in a lower yield (**1 m**→**3 ma**) while substrates with 2° and 3° alkyl groups generally displayed better reactivity to the reductive coupling (**1 n**–**r**→**3 na**–**ra**).

**Scheme 3:**
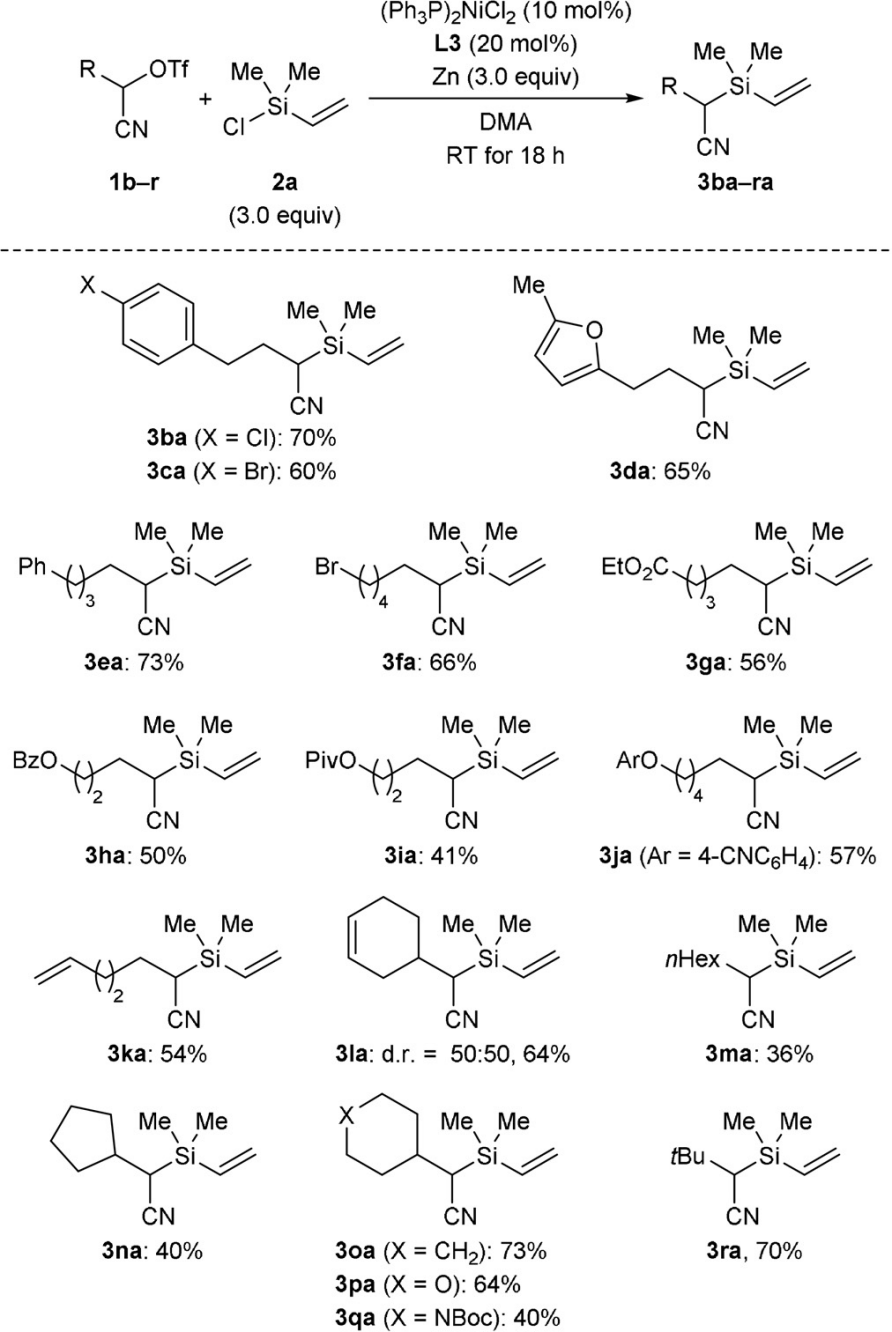
Scope II: Variation of the α‐triflyloxy nitrile. All reactions were performed on a 0.20 mmol scale with the isolated yield determined after flash chromatography on silica gel. Boc=*tert*‐butoxycarbonyl, Bz=benzoyl, Piv=pivaloyl.

To learn whether this reductive coupling proceeds through a radical intermediate, we added excess 2,2,6,6‐tetramethylpiperidin‐1‐oxyl (TEMPO) to the model reaction (**1 a**→**3 aa**; Scheme 4, top); there was hardly any effect on the yield. A radical‐probe experiment was done with cyclopropyl‐substituted **1 s** (middle). While the yield of **3 sa** was low, no ring‐opening product was detected (gray box). The corresponding α‐bromo nitrile led to the same outcome, furnishing **3 sa** in 21 % yield (see the Supporting Information for details). Also, there was no competing radical cyclization seen in the cross‐electrophile coupling of **1 k** and **2 a** (see Scheme 3). In turn, the reaction of (*R*)‐**1 a** (99 % ee)^[4a]^ under the standard reaction conditions led to complete racemization (bottom), proving that the reductive coupling reaction is not stereospecific (cf. Ref. [4a]). The involvement of silyl radicals seems unlikely as no disilane was detected by GC‐MS analysis when using vinyl‐substituted chlorosilane **2 a**. Moreover, trapping of the possible silyl radical with added alkenes was unsuccessful (not shown).

**Scheme 4:**
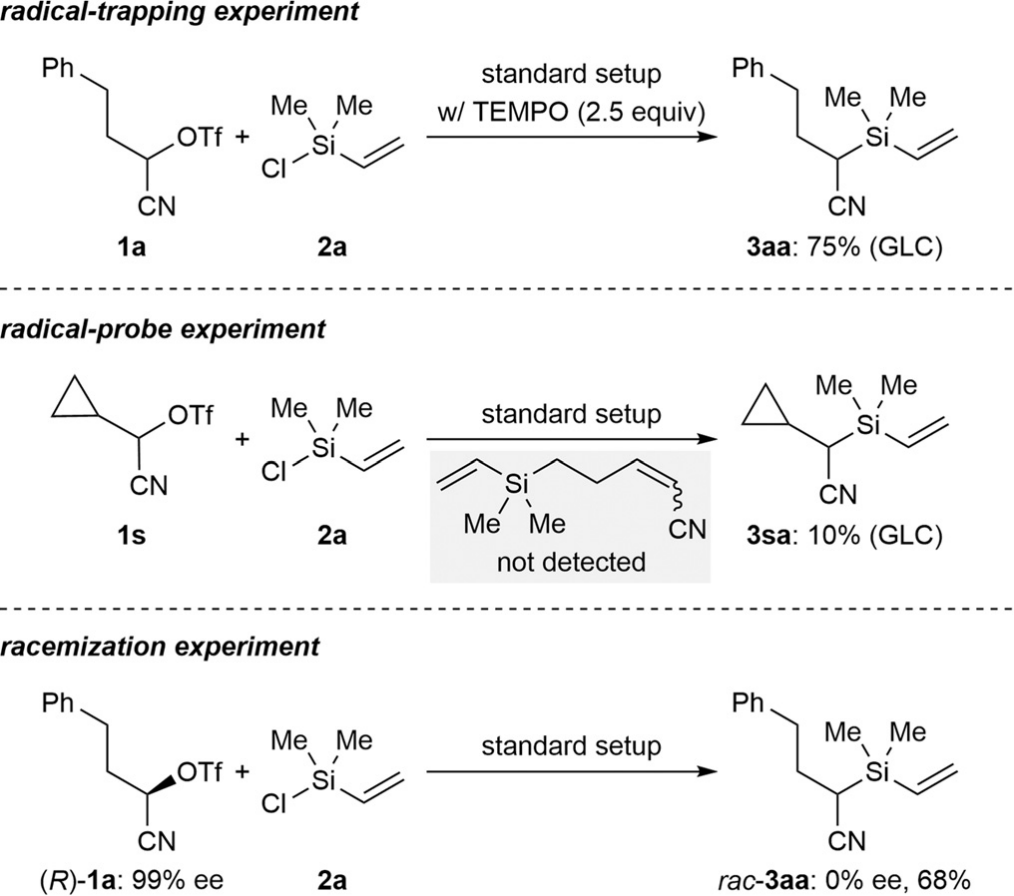
Mechanistic control experiments.

On the basis of the above results and previously reported mechanistic proposals,^[12]^ we envision the Ni^0^→Ni^II^→Ni^I^→Ni^III^→Ni^I^→Ni^0^ catalytic cycle outlined in Scheme 5. Oxidative addition of the C(sp^3^)−X bond in **1** to an in situ‐generated nickel(0) complex results in the formation of an alkylnickel(II) intermediate. This is the step where racemization could occur despite lack of evidence for radical intermediates (cf. Scheme 4).[^11a^, ^12a^] One‐electron reduction by zinc metal gives an alkylnickel(I) intermediate to which the Si−Cl bond of **2** oxidatively adds. The resulting alkyl(silyl)nickel(III) complex undergoes reductive elimination with formation of the C(sp^3^)−Si bond in **3**. The released nickel(I) complex will be eventually reduced to nickel(0) by the zinc reductant. An alternative order of events, that is oxidative addition of the chlorosilane **2** prior to that of the activated alkyl triflate **1**, cannot be ruled out at this stage.

**Scheme 5:**
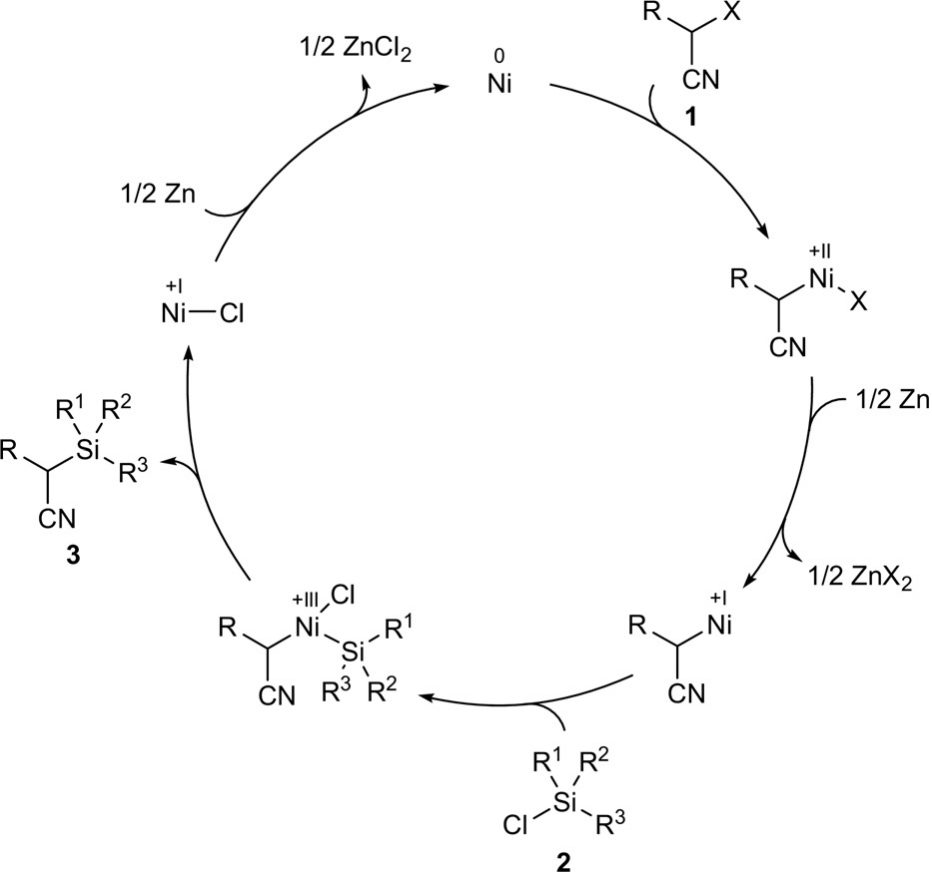
Proposed catalytic cycle (X=OTf as well as Cl and Br).

To summarize, we introduced herein a nickel/zinc‐catalyzed cross‐electrophile coupling of α‐cyano alkyl electrophiles and chlorosilanes to construct C(sp^3^)−Si bonds. It is the first example of a reductive cross‐coupling of an sp^3^‐hybridized carbon and a silicon electrophile. The method provides access to a range of α‐silylated nitriles which can, for example, be further employed in Hiyama cross‐coupling reactions.^[13]^ The extension to unactivated alkyl electrophiles and the development of asymmetric version are currently under investigation in our laboratory.

## Conflict of interest

The authors declare no conflict of interest.

## Supporting information

As a service to our authors and readers, this journal provides supporting information supplied by the authors. Such materials are peer reviewed and may be re‐organized for online delivery, but are not copy‐edited or typeset. Technical support issues arising from supporting information (other than missing files) should be addressed to the authors.

Supporting Information
