# Supplementary material for: Nickel‐Catalyzed, Reductive C(sp3)−Si Cross‐Coupling of α‐Cyano Alkyl Electrophiles and Chlorosilanes
Source: Angew Chem Int Ed Engl. 2021 Jul 16;60(34):18587–90. doi: 10.1002/anie.202107492 (PMC8456968; doi:10.1002/anie.202107492)
Supplement: Supplementary file 1 — Supporting Information [file ANIE-60-18587-s001.pdf]

Angewandte Chemie
Eine Zeitschrift der Gesellschaft Deutscher Chemiker

Supporting Information

Nickel-Catalyzed, Reductive C(sp^3)–Si Cross-Coupling of α-Cyano Alkyl Electrophiles and Chlorosilanes

*Liangliang Zhang and Martin Oestreich**

anie_202107492_sm_miscellaneous_information.pdf

Table of Contents

1. General Information 3

2. Optimization Study..4

3. General Procedures..8

4. Experimental Details for the Preparation of α-Triflyloxy Nitriles. 9

5. Experimental Details for Nickel-Catalyzed Reductive
Cross-Coupling of α-Cyano Alkyl Electrophiles and Chlorosilanes 11

6. Synthesis of **3aa** on 1.0-mmol Scale..21

7. Mechanistic Experiments 22

8. NMR Spectra 24

9. References 105

1. General Information

All reactions were performed in flame-dried glassware using conventional Schlenk techniques under a static pressure of nitrogen unless stated otherwise. Liquids and solutions were transferred with syringes. Solvents (THF, toluene, Et_2O, and CH_2Cl_2) were dried and purified following standard procedures. Dry DMA, DMF, NMP, MeCN, and DMSO were purchased from commercial sources and used as received. Technical grade solvents for extraction or chromatography (cyclohexane, ethyl acetate, *tert*-butyl methyl ether, CH_2Cl_2, Et_2O, and methanol) were distilled prior to use. All nickel catalysts, chlorosilanes, and ligands were purchased from Sigma Aldrich, TCI, ABCR, Fisher, and Strem. **1a**,[S1-S4] **1b**–**c**,[S3] **1f**,[S4] **1g**,[S3] **1h**–**l**,[S4], **1o**,[S1,S3,S4] **1p**,[S3] **1q**,[S3] and **1r**[S1,S3] were synthesized according to the reported procedure. 2-Bromo-4-phenylbutanenitrile,[S5,S6] 2-chloro-4-phenylbutanenitrile,[S7] 2-bromo-2-cyclopropylacetonitrile,[S6] and (*R*)-1-cyano-3-phenylpropyl trifluoromethanesulfonate[S3] were synthesized according to reported procedures. All spectroscopic data were in agreement with those reported. Analytical thin layer chromatography (TLC) was performed on ALUGRAM® Xtra SIL G/UV_{254} TLC-Sheets by Macherey-Nagel. Flash column chromatography was performed on silica gel 60 (40-63 µm, 230-400 mesh, ASTM) by Grace using the indicated solvents. ^1H, ^{13}C, ^{19}F, and ^{29}Si NMR spectra were recorded in $CDCl_3$ on Bruker AV400 or AV500 instruments. Chemical shifts were reported in parts per million (ppm) and were referenced to the residual solvent resonance as the internal standard ($CHCl_3$: δ = 7.26 ppm for ^1H NMR and $CDCl_3$: δ = 77.16 ppm for ^{13}C NMR). All other nuclei (^{19}F and ^{29}Si) were referenced in compliance with the unified scale for NMR chemical shifts as recommended by the IUPAC stating the chemical shift relative to $BF_3{\cdot}Et_2O$, CCl_3F, and Me_4Si. Data were reported as follows: chemical shift, multiplicity (br = broad signal, s = singlet, d = doublet, t = triplet, q = quartet, sept = septet, m = multiplet, m_c = centrosymmetric multiplet), coupling constants (Hz), and integration. Gas liquid chromatography (GLC) was performed on an *Agilent Technologies 7820A* gas chromatograph equipped with a HP-5 capillary column (30 m × 0.32 mm, 0.25 µm film thickness) by *Agilent Technologies/CS-Chromatographie Service* using the following program: N_2 carrier gas, injection temperature 250 °C, detector temperature 300 °C, flow rate: 1.7 mL/min; temperature program: start temperature 40 °C, heating rate 10 °C/min, end temperature 280 °C for 10 min. Infrared (IR) spectra were recorded on an *Agilent Technologies Cary* 630 FT-IR spectrometer equipped with an ATR unit and the signals were reported in wave-numbers (cm^{-1}). Melting points (m.p.) were determined with a Stuart Scientific SMP20 melting point apparatus and were not corrected. Enantiomeric excesses were determined by analytical high performance liquid chromatography (HPLC) analysis on an *Agilent Technologies* 1290 Infinity instrument with a chiral stationary phase using a *Daicel* Chiralcel IC column (*n*-heptane/isopropanol mixtures as solvent). High resolution mass spectra (HRMS) were obtained from the Analytical Facility at the *Institut für Chemie, Technische Universität Berlin* on a Thermo Fisher Scientific LTQ Orbitrap XL apparatus using APCI techniques with a linear ion trap analyzer. Optical rotations were measured on a *Schmidt & Haensch Polartronic* H532 Polarimeter with $[\alpha]^{t}_{\lambda}$ values reported in 10^{-1} (° cm^2 g^{-1}); with the concentration *c* in g/100 mL and λ indicate.

2. Optimization Study

General Procedure for the Optimization Reactions: To an oven-dried 20-mL Schlenk tube equipped with a magnetic stir bar were subsequently added the indicated nickel salt (10 mol%), ligand (20 mol%), and reductant (0.60 mol, 3.0 equiv). The tube was placed under vacuum and backfilled with N_2 (3 times). A solution of substrate **1a** (58.7 mg, 0.20 mmol, 1.0 equiv) and chlorosilane **2a** (72.4 mg, 0.60 mmol, 3.0 equiv) in 1.5 mL of the indicated solvent was then added by syringe, and the resultung mixture was maintained with stirring at room temperature for 18 h. After the indicated reaction time, the mixture was diluted with EtOAc (10 mL) and washed with water. The organic phase was then dried over anhydrous $MgSO_4$, filtered, and concentrated *in vacuo*. The residue was dissolved with 1 mL *tert*-butyl methyl ether and subjected to GLC analysis with tetracosane as the internal standard.

Table S1: Screening of nickel catalysts.[a,b]

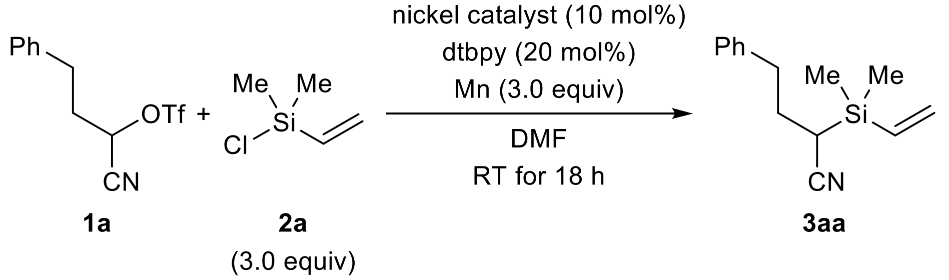

Entry	Nickel Catalyst	Yield(%)
1	$NiCl_2$	41
2	$NiBr_2$	44
3	NiI_2	45
4	$Ni(acac)_2$	43
5	$Ni(OTf)_2$	32
6	$NiOAc\cdot 4H_2O$	43
7	$NiSO_4\cdot 4H_2O$	22
8	$Ni(ClO_4)_2\cdot 6H_2O$	43
9	$NiBr_2\cdot$glyme	45
**10**	**$(Ph_3P)_2NiCl_2$**	**52**
11	$(Ph_3P)_2NiBr_2$	49
12	$Ni(cod)_2$	19
13	$NiBr_2\cdot$diglyme	38
14	$Ni(dppe)Cl_2$	21
15	$Ni(dppp)Cl_2$	46
16	$(Cy_3P)_2NiCl_2$	29

[a] All reactions were performed on a 0.20 mmol scale. [b] Yield was determined by GLC analysis with tetracosane as an internal standard.

Table S2: Screening of solvents and reductants. [a,b]

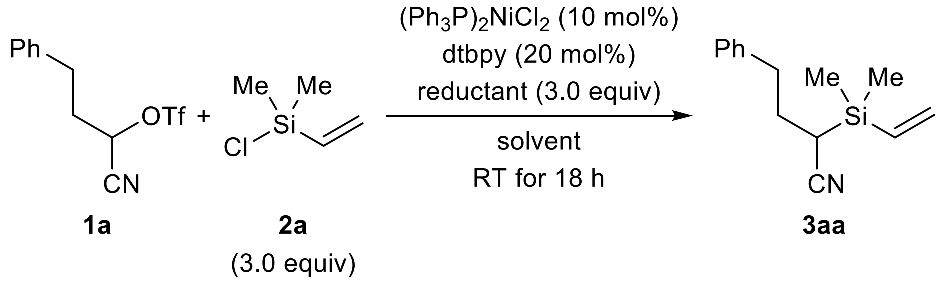

Entry	Solvent	Reductant	Yield(%)
1	DMF	Mn	52
2	Toluene	Mn	0
3	THF	Mn	4
4	CH_3CN	Mn	15
5	DMSO	Mn	0
6	DMI	Mn	11
7	DMPU	Mn	12
8	DMA	Mn	54
9	NMP	Mn	55
10	DMF	Zn	50
11	NMP	Zn	57
**12**	**DMA**	**Zn**	**90 (76)[c]**

[a] All reactions were performed on a 0.20 mmol scale. [b] Yield was determined by GLC analysis with tetracosane as an internal standard. [c] Isolated yield after flash chromatography on silica gel.

Table S3: Screening of ligands.[a,b]

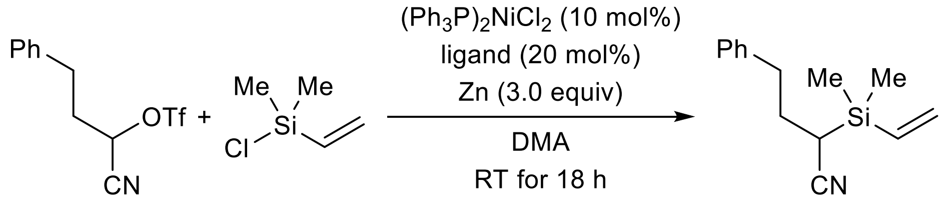

1a **2a** (3.0 equiv) **3aa**

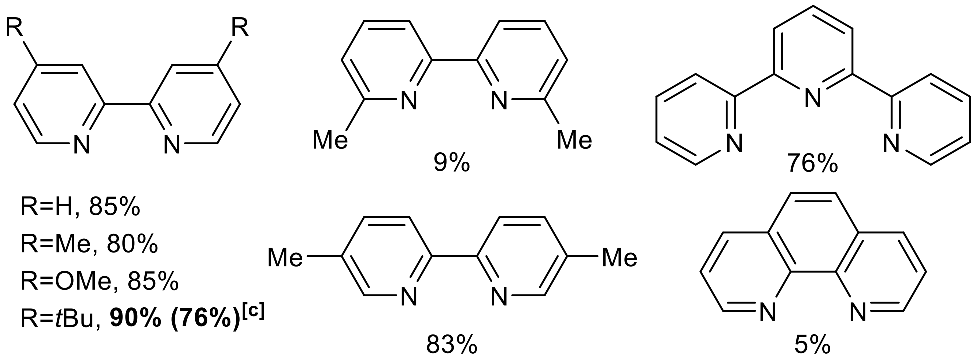

R=H, 85%
R=Me, 80%
R=OMe, 85%
R=*t*Bu, **90% (76%)**[c]

[a] All reactions were performed on a 0.20 mmol scale. [b] Yield was determined by GLC analysis with tetracosane as an internal standard. [c] Isolated yield after flash chromatography on silica gel.

Table S4: Screening of other conditions.[a,b]

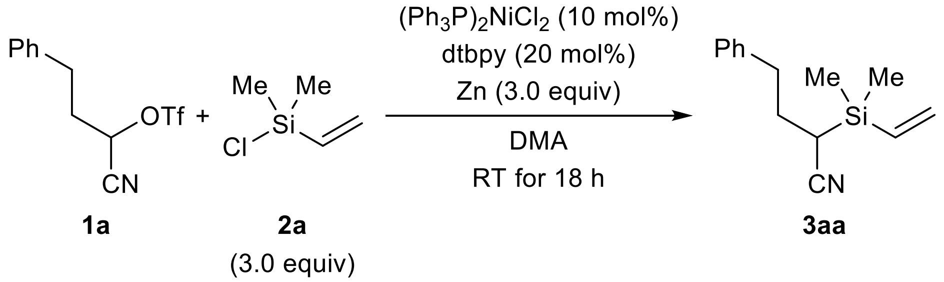

Entry	Variations	Yield(%)
**1**	**none**	**90(76)[c]**
2	Cl instead of OTf	80
2	Br instead of OTf	75
3	0 °C instead of rt	83
4	40 °C instead of rt	81
5	w/o $(Ph_3P)_2NiCl_2$	0
6	w/o Zn	0
7	w/o dtbpy	70
8	3-phenylpropyl trifluoromethanesulfonate instead of **1a**	0
9	bromocyclohexane instead of **1a**	0

[a] All reactions were performed on a 0.20 mmol scale. [b] Yield was determined by GLC analysis with tetracosane as an internal standard. [c] Isolated yield after flash chromatography on silica gel.

3. General Procedures

3.1. General Procedures for the Preparation of α-Triflyloxy Ntriles (GP1)

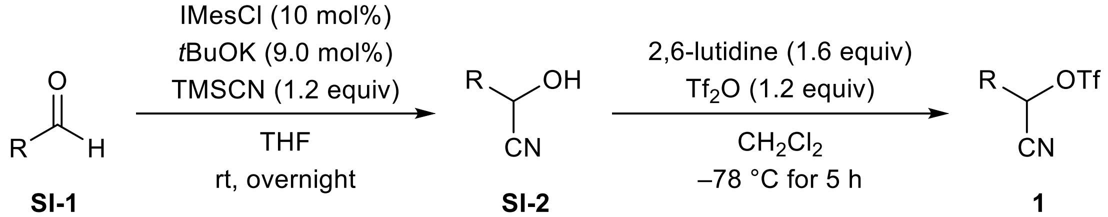

SI-1 **SI-2** **1**

To an oven-dried 50-mL Schlenk flask equipped with a magnetic stir bar is added IMesCl (171 mg, 0.50 mmol, 0.10 equiv). The flask is placed under vacuum and backfilled with N_2 (3 times). A solution of the corresponding aldehyde **SI-1** (5.0 mmol, 1.0 equiv) and TMSCN (595 mg, 6.0 mmol, 1.2 equiv) in THF (10 mL) is then added by syringe, and the mixture is stirred for 5 min. KO*t*Bu (50.5 mg, 0.45 mmol, 0.090 equiv) is added in one portion, and the reaction mixture is maintained with stirring at room temperature overnight. After TLC analysis indicates complete conversion, the mixture is quenched by an aqueous solution of HCl (1M) and extracted with CH_2Cl_2 (3×10 mL). The combined organic layers are dried ($MgSO_4$), filtered, and concentrated *in vacuo* to give cyanohydrins **SI-2** as colorless oils. These are directly used in the next step.

To an oven-dried 50-mL Schlenk flask equipped with a magnetic stir bar are subsequently added the crude cyanohydrins **SI-2** and 2,6-lutidine (857 mg, 8.0 mmol, 1.6 equiv) at –78 °C under an atmosphere of N_2. CH_2Cl_2 (10 mL) is added by syringe. Tf_2O (1.69 g, 6.0 mmol, 1.2 equiv) is then added dropwise, and the reaction mixture is stirred at this temperature for 5 h. After the indicated reaction time, the mixture is quenched with distilled water (10 mL) and extracted with CH_2Cl_2 (3 × 10 mL). The combined organic layers are dried ($MgSO_4$), filtered, and concentrated under reduced pressure. The residue is purified by column chromatography on silica gel, and α-triflyloxy nitriles **1** are obtained as colorless oils.

3.2. General Procedures for the Nickel-Catalyzed Reductive Cross-Coupling of α-Cyano Alkyl Electrophiles and Chlorosilanes (GP2)

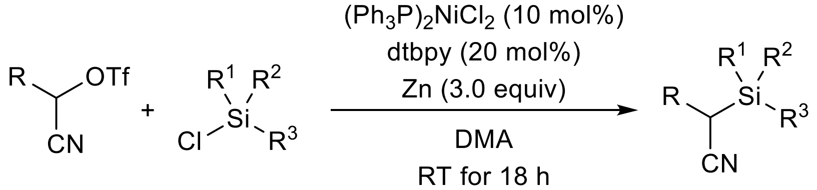

1 **2** **3**

(3.0 equiv)

To an oven-dried 20-mL Schlenk tube equipped with a magnetic stir bar are subsequently added $(Ph_3P)_2NiCl_2$ (13.1 mg, 0.020 mmol, 0.10 equiv), dtbpy (**L3**, 10.7 mg, 0.040 mmol, 0.20 equiv), and Zn (39.2 mg, 0.60 mol, 3.0 equiv). The tube is placed under vacuum and backfilled with N_2 (3 times). A solution of substrate **1** (0.20 mmol, 1.0 equiv) and chlorosilane **2** (0.60 mmol, 3.0 equiv) in DMA (1.5 mL) is added by syringe, and the mixture is maintained with stirring at room temperature for 18 h. After the indicated reaction time, the mixture is diluted with EtOAc (10 mL) and washed with water. The organic phase is then dried over anhydrous $MgSO_4$, filtered, and concentrated under reduced pressure. The crude residue is purified by column chromatography on silica gel.

4. Experimental Details for the Preparation of α-Triflyloxy Nitriles.

4.1. 1-Cyano-3-(5-methylfuran-2-yl)propyl trifluoromethanesulfonate (1d)

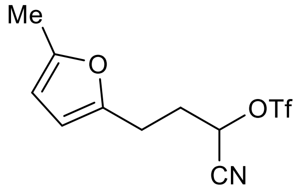

1d

$C_{10}H_{10}F_3NO_4S$

M = 297.25 g/mol

Prepared from 3-(5-methylfuran-2-yl)propanal (691 mg, 5.0 mmol, 1.0 equiv), TMSCN (595 mg, 6.0 mmol, 1.2 equiv), and Tf_2O (1.69 g, 6.0 mmol, 1.2 equiv) following the **GP1**. The product **1d** was obtained as a colorless oil (900 mg, 61% yield).

R$_f$ = 0.50 (10/1 cyclohexane/EtOAc). **IR** (ATR): $\tilde{\nu}$ = 2927, 1570, 1421, 1209, 1136, 1018, 915, 844, 785 cm^{-1}. **^{1}H NMR** (500 MHz, $CDCl_3$): δ 6.00 (d, *J* = 3.0 Hz, 1H), 5.89 (d, *J* = 2.7 Hz, 1H), 5.36 (t, *J* = 6.9 Hz, 1H), 2.87 (t, *J* = 7.3 Hz, 2H), 2.48–2.40 (m, 2H), 2.26 (s, 3H) ppm. **^{13}C{^{1}H} NMR** (125 MHz, CD_3Cl): δ 152.1, 149.0, 118.4 (q, *J* = 320.1 Hz), 113.8, 108.1, 106.4, 71.0, 32.7, 22.9, 13.5 ppm. **^{19}F{^{1}H} NMR** (471 MHz, $CDCl_3$): δ = –74.1 ppm. **HRMS** (APCI) m/z: $[M+H]^+$ Calcd for $C_{10}H_{11}F_3NO_4S^+$ 298.0355; Found 298.0352.

4.2. 1-Cyano-5-phenylpentyl trifluoromethanesulfonate (1e)

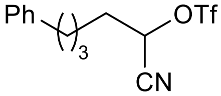

1e

$C_{13}H_{14}F_3NO_3S$

M = 321.31 g/mol

Prepared from 5-phenylpentanal (811 mg, 5.0 mmol, 1.0 equiv), TMSCN (595 mg, 6.0 mmol, 1.2 equiv), and Tf_2O (1.69 g, 6.0 mmol, 1.2 equiv) following the **GP1**. The product **1e** was obtained as a colorless oil (1.22 g, 76% yield).

R$_f$ = 0.50 (10/1 cyclohexane/EtOAc). **IR** (ATR): $\tilde{\nu}$ = 2937, 1495, 1420, 1208, 1136, 1030, 907, 852, 748, 699 cm^{-1}. **^{1}H NMR** (500 MHz, $CDCl_3$): δ 7.30–7.26 (m, 2H), 7.21–7.19 (m, 1H), 7.16–7.14 (m, 2H), 5.27 (t, *J* = 6.7 Hz, 1H), 2.64 (t, *J* = 7.5 Hz, 2H), 2.10–2.04 (m, 2H), 1.74–1.63 (m, 2H), 1.61–1.53 (m, 2H) ppm. **^{13}C{^{1}H} NMR** (125 MHz, CD_3Cl): δ 141.4, 128.6, 128.4, 126.2, 118.4 (q, *J* = 320.0 Hz), 113.9, 72.1, 35.3, 33.6, 30.3, 23.7 ppm. **^{19}F{^{1}H} NMR** (471 MHz, $CDCl_3$): δ = –73.9 ppm. **HRMS** (APCI) m/z: $[M+H]^+$ Calcd for $C_{13}H_{15}F_3NO_3S^+$ 322.0719; Found 322.0715.

4.3. 1-Cyanoheptyl trifluoromethanesulfonate (1m)

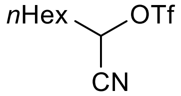

1m

$C_9H_{14}F_3NO_3S$

M = 273.27 g/mol

Prepared from heptanal (571 mg, 5.0 mmol, 1.0 equiv), TMSCN (595 mg, 6.0 mmol, 1.2 equiv), and Tf_2O (1.69 g, 6.0 mmol, 1.2 equiv) following the **GP1**. The product **1m** was obtained as a colorless oil (925 mg, 68% yield).
R$_f$ = 0.60 (10/1 cyclohexane/EtOAc). **IR** (ATR): $\tilde{\nu}$ = 2932, 1422, 1211, 1139, 938, 850, 725 cm^{-1}.**^{1}H NMR** (500 MHz, $CDCl_3$): δ 5.33 (t, *J* = 6.7 Hz, 1H), 2.13–2.07 (m, 2H), 1.60–1.53 (m, 2H), 1.43–1.36 (m, 2H), 1.35–1.28 (m, 4H), 0.92–0.89 (t, *J* = 7.1 Hz, 3H) ppm. **^{13}C{^{1}H} NMR** (125 MHz, CD_3Cl): δ 118.5 (q, *J* = 320.1 Hz), 114.0, 72.2, 33.9, 31.4, 28.3, 24.2, 22.5, 14.0 ppm. **^{19}F{^{1}H} NMR** (471 MHz, $CDCl_3$): δ = –74.2 ppm. **HRMS** (APCI) m/z: [M–OTf]$^+$ Calcd for $C_8H_{14}N^+$ 124.1121; Found 124.1120.

4.4. Cyano(cyclopentyl)methyl trifluoromethanesulfonate (1n)

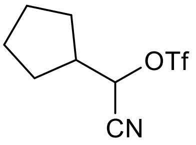

1n

$C_8H_{10}F_3NO_3S$

M = 257.23 g/mol

Prepared from cyclopentanecarbaldehyde (491 mg, 5.0 mmol, 1.0 equiv), TMSCN (595 mg, 6.0 mmol, 1.2 equiv), and Tf_2O (1.69 g, 6.0 mmol, 1.2 equiv) following the **GP1**. The product **1n** was obtained as a colorless oil (913 mg, 71% yield).
R$_f$ = 0.50 (10/1 cyclohexane/EtOAc). **IR** (ATR): $\tilde{\nu}$ = 2964, 2876, 1419, 1206, 1136, 920, 839 cm^{-1}. **^{1}H NMR** (500 MHz, $CDCl_3$): δ 5.24 (d, *J* = 6.9 Hz, 1H), 2.61–2.53(m, 1H), 2.02–1.93 (m, 2H), 1.81–1.72 (m, 2H), 1.71–1.63 (m, 2H), 1.58–1.45 (m, 2H) ppm. **^{13}C{^{1}H} NMR** (125 MHz, CD_3Cl): δ 117.2 (q, *J* = 320.0 Hz), 113.8, 75.6, 43.1, 28.7, 28.3, 25.4, 25.3 ppm. **^{19}F{^{1}H} NMR** (471 MHz, $CDCl_3$): δ = –74.2 ppm. **HRMS** (APCI) m/z: [M–OTf]$^+$ Calcd for $C_7H_{10}N^+$ 108.0808; Found 108.0809.

4.5. Cyano(cyclopropyl)methyl trifluoromethanesulfonate (1s)

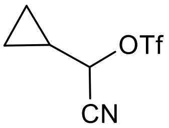

1s

$C_6H_6F_3NO_3S$

M = 229.17 g/mol

Prepared from cyclopentanecarbaldehyde (350 mg, 5.0 mmol, 1.0 equiv), TMSCN (595 mg, 6.0 mmol, 1.2 equiv), and Tf_2O (1.69 g, 6.0 mmol, 1.2 equiv) following the **GP1**. The product **1s** was obtained as a colorless oil (745 mg, 65% yield).
R$_f$ = 0.40 (5/1 cyclohexane/EtOAc). **IR** (ATR): $\tilde{\nu}$ = 2917, 2849, 1414, 1202, 1139, 1059, 960, 912, 874, 792 cm^{-1}. **^{1}H NMR** (500 MHz, $CDCl_3$): δ 5.29 (dd, J_1 = 7.9 Hz, J_2 = 7.5 Hz, 1H), 3.45–3.38 (m, 1H), 2.60–2.53 (m, 1H), 2.47–2.35 (m, 2H), 2.08–1.97 (m, 1H) ppm. **^{13}C{^{1}H} NMR** (125 MHz, CD_3Cl): δ 118.5 (q, *J* = 320.0 Hz), 117.2, 78.9, 31.1, 29.2, 17.9 ppm. **^{19}F{^{1}H} NMR** (471 MHz, $CDCl_3$): δ = –74.9 ppm. **HRMS** (APCI) m/z: [M+H]$^+$ Calcd for $C_6H_7F_3NO_3S^+$ 230.0093; Found 230.0098.

5. Experimental Details for Nickel-Catalyzed Reductive Cross-Coupling of α-Cyano Alkyl Electrophiles and Chlorosilanes

5.1. 2-(Dimethyl(vinyl)silyl)-4-phenylbutanenitrile (3aa)

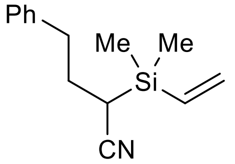

3aa

$C_{14}H_{19}NSi$

M = 229.40 g/mol

Prepared from 1-cyano-3-phenylpropyl trifluoromethanesulfonate (**1a**, 58.7 mg, 0.20 mmol, 1.0 equiv) with chlorodimethyl(vinyl)silane (**2a**, 72.4 mg, 0.60 mmol, 3.0 equiv) at rt in DMA for 18 h according to **GP2**. Purification by flash column chromatography on silica gel (20:1 cyclohexane/EtOAc) afforded **3aa** as a colorless oil (34.8 mg, 76% yield).

R$_f$ = 0.60 (10/1 cyclohexane/EtOAc). **IR** (ATR): $\tilde{\nu}$ = 3026, 2946, 2859, 2220, 1601, 1495, 1453, 1406, 1254, 1107, 1009, 960, 815, 786, 748, 700 cm^{-1}. **^{1}H NMR** (500 MHz, $CDCl_3$): δ 7.29–7.26 (m, 2H), 7.22–7.16 (m, 3H), 6.09–6.03 (m, 2H), 5.80–5.76 (m, 1H), 3.00–2.95 (m, 1H), 2.70–2.65 (m, 1H), 1.86–1.82 (m, 1H), 1.77–1.71 (m, 2H), 0.23 (s, 6H) ppm. **^{13}C{^{1}H} NMR** (125 MHz, CD_3Cl): δ 140.4, 135.6, 134.0, 128.72, 128.66, 126.5, 121.8, 35.8, 28.7, 17.7, –4.8, –5.2 ppm. **^{29}Si{^{1}H} DEPT NMR** (99 MHz, $CDCl_3$): δ –2.0 ppm. **HRMS** (APCI) m/z: [M+H]$^+$ Calcd for $C_{14}H_{20}NSi^+$ 230.1360; Found 230.1357.

5.2. 4-Phenyl-2-(trivinylsilyl)butanenitrile (3ab)

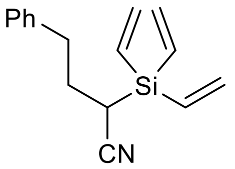

3ab

$C_{16}H_{19}NSi$

M = 253.42 g/mol

Prepared from 1-cyano-3-phenylpropyl trifluoromethanesulfonate (**1a**, 58.7 mg, 0.20 mmol, 1.0 equiv) with chlorotrivinylsilane (**2b**, 86.8 mg, 0.60 mmol, 3.0 equiv) at rt in DMA for 18 h according to **GP2**. Purification by flash column chromatography on silica gel (20:1 cyclohexane/EtOAc) afforded **3ab** as a colorless oil (35.5 mg, 70% yield).

R$_f$ = 0.50 (10/1 cyclohexane/EtOAc). **IR** (ATR): $\tilde{\nu}$ = 3056, 3026, 2947, 2860, 2221, 1592, 1495, 1453, 1404, 1106, 1008, 963, 739, 700 cm^{-1}. **^{1}H NMR** (500 MHz, $CDCl_3$): δ 7.32–7.29 (m, 2H), 7.24–7.19 (m, 3H), 6.28–6.12 (m, 6H), 5.95–5.91 (m, 3H), 3.04–2.98 (m, 1H), 2.79–2.69 (m, 1H), 2.01–1.90 (m, 2H), 1.88–1.80 (m, 1H) ppm. **^{13}C{^{1}H} NMR** (125 MHz, CD_3Cl): δ 140.3, 138.4, 129.9, 128.75, 128.70, 126.5, 121.4, 35.7, 28.8, 16.4 ppm. **^{1}H/^{29}Si HMQC NMR** (500/99 MHz, $CDCl_3$, optimized for *J* = 7.0 Hz): δ 6.20/–20.5, 5.94/–20.5, 1.95/–20.5 ppm. **HRMS** (APCI) m/z: [M+H]$^+$ Calcd for $C_{16}H_{20}NSi^+$ 254.1360; Found 254.1358.

5.3. 2-(Ethyldimethylsilyl)-4-phenylbutanenitrile (3ac)

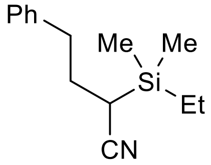

3ac

$C_{14}H_{21}NSi$

M = 231.41 g/mol

Prepared from 1-cyano-3-phenylpropyl trifluoromethanesulfonate (**1a**, 58.7 mg, 0.20 mmol, 1.0 equiv with chloro(ethyl)dimethylsilane (**2c**, 73.6 mg, 0.60 mmol, 3.0 equiv) at rt in DMA for 18 h according to **GP2**. Purification by flash column chromatography on silica gel (20:1 cyclohexane/EtOAc) afforded **3ac** as a colorless oil (15.3 mg, 33% yield).
R$_f$ = 0.60 (10/1 cyclohexane/EtOAc). **IR** (ATR): $\tilde{\nu}$ = 3063, 3027, 2954, 2875, 2219, 1603, 1496, 1454, 1254, 1108, 1009, 840, 814, 748, 700 cm^{-1}. ^{1}**H NMR** (500 MHz, $CDCl_3$): δ 7.33–7.30 (m, 2H), 7.24–7.20 (m, 3H), 3.05–2.99 (m, 1H), 2.74–2.68 (m, 1H), 1.94–1.85 (m, 1H), 1.78–1.72 (m, 2H), 0.95 (t, *J* = 7.9 Hz, 3H), 0.73–0.61 (m, 2H), 0.15 (s, 3H), 0.14 (s, 3H) ppm. ^{13}**C{**^{1}**H} NMR** (125 MHz, CD_3Cl): δ 140.5, 128.8, 128.7, 126.5, 122.2, 35.9, 28.8, 17.1, 7.1, 5.5, –5.17, –5.23 ppm. ^{1}**H/**^{29}**Si HMQC NMR** (500/99 MHz, $CDCl_3$, optimized for *J* = 7.0 Hz): δ 1.78/8.6, 0.95/8.6, 0.15/8.6 ppm. **HRMS** (APCI) m/z: $[M+H]^+$ Calcd for $C_{14}H_{22}NSi^+$ 232.1516; Found 232.1515.

5.4. 4-Phenyl-2-(trimethylsilyl)butanenitrile (3ad)

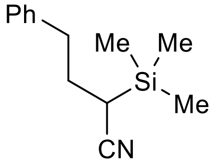

3ad

$C_{13}H_{19}NSi$

M = 217.39 g/mol

Prepared from 1-cyano-3-phenylpropyl trifluoromethanesulfonate (**1a**, 58.7 mg, 0.20 mmol, 1.0 equiv) with chlorotrimethylsilane (**2d**, 65.2 mg, 0.60 mmol, 3.0 equiv) at rt in DMA for 18 h according to **GP2**. Purification by flash column chromatography on silica gel (20:1 cyclohexane/EtOAc) afforded **3ad** as a colorless oil (17.4 mg, 40% yield).
R$_f$ = 0.60 (10/1 cyclohexane/EtOAc). **IR** (ATR): $\tilde{\nu}$ = 3062, 3027, 2954, 2219, 1603, 1496, 1453, 1253, 1107, 844, 748, 700 cm^{-1}. ^{1}**H NMR** (500 MHz, $CDCl_3$): δ 7.33–7.30 (m, 2H), 7.24–7.21 (m, 3H), 3.05–2.99 (m, 1H), 2.75–2.69 (m, 1H), 1.93–1.85 (m, 1H), 1.80–1.73 (m, 2H), 0.18 (s, 9H) ppm. ^{13}**C{**^{1}**H} NMR** (125 MHz, CD_3Cl): δ 140.5, 128.8, 128.7, 126.5, 122.1, 35.9, 28.7, 18.4, –3.1 ppm. ^{1}**H/**^{29}**Si HMQC NMR** (500/99 MHz, $CDCl_3$, optimized for *J* = 7.0 Hz): δ 1.75/6.8, 0.18/6.8 ppm. **HRMS** (APCI) m/z: $[M+H]^+$ Calcd for $C_{13}H_{20}NSi^+$ 218.1360; Found 218.1361.

5.5. 4-(4-Chlorophenyl)-2-(dimethyl(vinyl)silyl)butanenitrile (3ba)

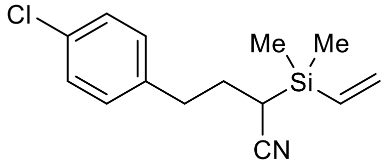

3ba

$C_{14}H_{18}ClNSi$

M = 263.84 g/mol

Prepared from 3-(4-chlorophenyl)-1-cyanopropyl trifluoromethanesulfonate (**1b**, 65.5 mg, 0.20 mmol, 1.0 equiv) with chlorodimethyl(vinyl)silane (**2a**, 72.4 mg, 0.60 mmol, 3.0 equiv) at rt in DMA for 18 h according to **GP2**. Purification by flash column chromatography on silica gel (20:1 cyclohexane/EtOAc) afforded **3ba** as a colorless oil (36.9 mg, 70% yield).

R$_f$ = 0.55 (10/1 cyclohexane/EtOAc). **IR** (ATR): $\tilde{\nu}$ = 3052, 2949, 2861, 2221, 1594, 1491, 1407, 1254, 1092, 1013, 960, 842, 816, 786, 709 cm^{-1}. **^{1}H NMR** (500 MHz, $CDCl_3$): δ 7.27 (d, *J* = 8.3 Hz, 2H), 7.12 (d, *J* = 8.3 Hz, 2H), 6.14–6.06 (m, 2H), 5.86–5.78 (m, 1H), 3.00–2.93 (m, 1H), 2.71–2.65 (m, 1H), 1.90–1.82 (m, 1H), 1.78–1.69 (m, 2H), 0.263 (s, 3H), 0.257 (s, 3H) ppm. **^{13}C{^{1}H} NMR** (125 MHz, CD_3Cl): δ 138.8, 135.7, 133.9, 132.3, 130.0, 128.9, 121.7, 35.1, 28.6, 17.7, –4.8, –5.2 ppm. **^{1}H/^{29}Si HMQC NMR** (500/99 MHz, $CDCl_3$, optimized for *J* = 7.0 Hz): δ 5.82/–2.2, 1.75/–2.2, 0.26/–2.2 ppm. **HRMS** (APCI) m/z: [M+H]$^+$ Calcd for $C_{14}H_{19}ClNSi^+$ 264.0970; Found 264.0968.

5.6. 4-(4-Bromophenyl)-2-(dimethyl(vinyl)silyl)butanenitrile (3ca)

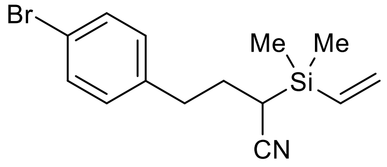

3ca

$C_{14}H_{18}BrNSi$

M = 308.29 g/mol

Prepared from 3-(4-bromophenyl)-1-cyanopropyl trifluoromethanesulfonate (**1c**, 74.4 mg, 0.20 mmol, 1.0 equiv) with chlorodimethyl(vinyl)silane (**2a**, 72.4 mg, 0.60 mmol, 3.0 equiv) at rt in DMA for 18 h according to **GP2**. Purification by flash column chromatography on silica gel (20:1 cyclohexane/EtOAc) afforded **3ca** as a colorless oil (36.8 mg, 60% yield).

R$_f$ = 0.55 (10/1 cyclohexane/EtOAc). **IR** (ATR): $\tilde{\nu}$ = 3052, 2948, 2861, 2220, 1592, 1487, 1405, 1254, 1101, 1072, 1010, 960, 816, 709 cm^{-1}. **^{1}H NMR** (500 MHz, $CDCl_3$): δ 7.42 (d, *J* = 8.3 Hz, 2H), 7.07 (d, *J* = 8.2 Hz, 2H), 6.14–6.06 (m, 2H), 5.86–5.77 (m, 1H), 2.98–2.92 (m, 1H), 2.70–2.64 (m, 1H), 1.90–1.82 (m, 1H), 1.77–1.68 (m, 2H), 0.262 (s, 3H), 0.256 (s, 3H) ppm. **^{13}C{^{1}H} NMR** (125 MHz, CD_3Cl): δ 139.3, 135.7, 133.9, 131.9, 130.4, 121.6, 120.4, 35.1, 28.6, 17.7, –4.8, –5.2 ppm. **^{1}H/^{29}Si HMQC NMR** (500/99 MHz, $CDCl_3$, optimized for *J* = 7.0 Hz): δ 5.82/–2.2, 1.75/–2.2, 0.26/–2.2 ppm. **HRMS** (APCI) m/z: [M+H]$^+$ Calcd for $C_{14}H_{19}BrNSi^+$ 308.0465; Found 308.0460.

5.7. 2-(Dimethyl(vinyl)silyl)-4-(5-methylfuran-2-yl)butanenitrile (3da)

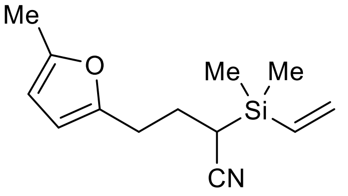

3da

$C_{13}H_{19}NOSi$

M = 233.39 g/mol

Prepared from 1-cyano-3-(5-methylfuran-2-yl)propyl trifluoromethanesulfonate (**1d**, 59.4 mg, 0.20 mmol, 1.0 equiv) with chlorodimethyl(vinyl)silane (**2a**, 72.4 mg, 0.60 mmol, 3.0 equiv) at rt in DMA for 18 h according to **GP2**. Purification by flash column chromatography on silica gel (20:1 cyclohexane/EtOAc) afforded **3da** as a colorless oil (30.2 mg, 65% yield).

R$_f$ = 0.40 (10/1 cyclohexane/EtOAc). **IR** (ATR): $\tilde{\nu}$ = 3054, 2953, 2221, 1569, 1452, 1406, 1255, 1218, 1105, 1018, 961, 841, 816, 783, 710 cm^{-1}. **^{1}H NMR** (500 MHz, $CDCl_3$): δ 6.17–6.09 (m, 2H), 5.93 (d, *J* = 3.0 Hz, 1H), 5.87–5.80 (m, 2H), 2.94–2.89 (m, 1H), 2.75–2.68 (m, 1H), 2.25 (s, 3H), 1.87–1.80 (m, 3H), 0.28 (s, 3H), 0.27 (s, 3H) ppm. **^{13}C{^{1}H} NMR** (125 MHz, CD_3Cl): δ 152.0, 151.1, 135.6, 134.0, 121.7, 106.9, 106.1, 28.2, 25.8, 17.7, 13.6, –4.8, –5.1 ppm. **^{29}Si{^{1}H} DEPT NMR** (99 MHz, $CDCl_3$): δ –2.0 ppm. **HRMS** (APCI) m/z: [M+H]$^+$ Calcd for $C_{13}H_{20}NOSi^+$ 234.1309; Found 234.1305.

5.8. 2-(Dimethyl(vinyl)silyl)-6-phenylhexanenitrile (3ea)

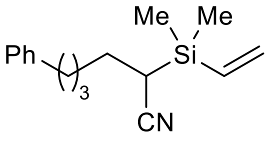

3ea

$C_{16}H_{23}NSi$

M = 257.45 g/mol

Prepared from 1-cyano-5-phenylpentyl trifluoromethanesulfonate (**1e**, 74.4 mg, 0.20 mmol, 1.0 equiv) with chlorodimethyl(vinyl)silane (**2a**, 72.4 mg, 0.60 mmol, 3.0 equiv) at rt in DMA for 18 h according to **GP2**. Purification by flash column chromatography on silica gel (20:1 cyclohexane/EtOAc) afforded **3ea** as a colorless oil (37.6 mg, 73% yield).

R$_f$ = 0.50 (10/1 cyclohexane/EtOAc). **IR** (ATR): $\tilde{\nu}$ = 3025, 2935, 2858, 2218, 1602, 1495, 1453, 1406, 1254, 1113, 1009, 959, 842, 785, 748, 700 cm^{-1}. **^{1}H NMR** (500 MHz, $CDCl_3$): δ 7.36–7.33 (m, 2H), 7.26–7.23 (m, 3H), 6.20–6.14 (m, 2H), 5.92–5.87 (m, 1H), 2.74–2.69 (m, 2H), 1.87–1.84 (m, 1H), 1.76–1.53 (m, 6H), 0.32 (s, 6H) ppm. **^{13}C{^{1}H} NMR** (125 MHz, CD_3Cl): δ 142.3, 135.4, 134.2, 128.49, 128.47, 125.9, 122.1, 35.8, 30.9, 29.7, 26.8, 18.4, –4.8, –5.1 ppm. **^{29}Si{^{1}H} DEPT NMR** (99 MHz, $CDCl_3$): δ –2.1 ppm. **HRMS** (APCI) m/z: [M+H]$^+$ Calcd for $C_{16}H_{24}NSi^+$ 258.1673; Found 258.1671.

5.9. 7-Bromo-2-(dimethyl(vinyl)silyl)heptanenitrile (3fa)

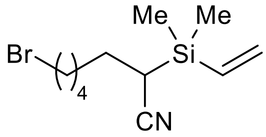

3fa

$C_{11}H_{20}BrNSi$

M = 274.28 g/mol

Prepared from 6-bromo-1-cyanohexyl trifluoromethanesulfonate (**1f**, 67.6 mg, 0.20 mmol, 1.0 equiv) with chlorodimethyl(vinyl)silane (**2a**, 72.4 mg, 0.60 mmol, 3.0 equiv) at rt in DMA for 18 h according to **GP2**. Purification by flash column chromatography on silica gel (20:1 cyclohexane/EtOAc) afforded **3fa** as a colorless oil (36.0 mg, 66% yield).

R$_f$ = 0.60 (10/1 cyclohexane/EtOAc). **IR** (ATR): $\tilde{\nu}$ = 3052, 2937, 2859, 2219, 1593, 1460, 1406, 1254, 1106, 1009, 959, 842, 785, 711 cm^{-1}. **^{1}H NMR** (500 MHz, $CDCl_3$): δ 6.18–6.09 (m, 2H), 5.88–5.81 (m, 1H), 3.41 (t, J = 6.8 Hz, 2H), 1.90–1.84 (m, 2H), 1.83–1.80 (m, 1H), 1.72–1.65 (m, 1H), 1.58–1.44 (m, 5H), 0.27 (s, 6H) ppm. **^{13}C{^{1}H} NMR** (125 MHz, CD_3Cl): δ 135.5, 134.1, 122.0, 33.7, 32.5, 29.2, 27.6, 26.7, 18.4, –4.7, –5.1 ppm. **^{29}Si{^{1}H} DEPT NMR** (99 MHz, $CDCl_3$): δ –2.1 ppm. **HRMS** (APCI) m/z: [M+H]$^+$ Calcd for $C_{11}H_{21}BrNSi^+$ 274.0621; Found 274.0622.

5.10. Ethyl 6-cyano-6-(dimethyl(vinyl)silyl)hexanoate (3ga)

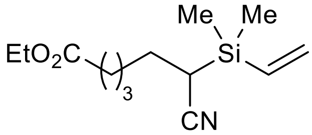

3ga

$C_{13}H_{23}NO_2Si$

M = 253.42g/mol

Prepared from ethyl 6-cyano-6-(((trifluoromethyl)sulfonyl)oxy)hexanoate (**1g**, 63.4 mg, 0.20 mmol, 1.0 equiv) with chlorodimethyl(vinyl)silane (**2a**, 72.4 mg, 0.60 mmol, 3.0 equiv) at rt in DMA for 18 h according to **GP2**. Purification by flash column chromatography on silica gel (10:1 cyclohexane/EtOAc) afforded **3ga** as a colorless oil (28.3 mg, 56% yield).

R$_f$ = 0.40 (5/1 cyclohexane/EtOAc). **IR** (ATR): $\tilde{\nu}$ = 2943, 2220, 1732, 1594, 1460, 1406, 1373, 1254, 1184, 1115, 1029, 961, 843, 785, 709 cm^{-1}. **^{1}H NMR** (500 MHz, $CDCl_3$): δ 6.15–6.08 (m, 2H), 5.87–5.80 (m, 1H), 4.12 (q, J = 7.2 Hz, 2H), 2.31 (t, $^3J_{5,4}$ = 7.2 Hz, 2H), 1.83–1.80 (m, 1H), 1.71–1.42 (m, 6H), 1.25 (t, J = 7.1 Hz, 3H), 0.27 (s, 6H) ppm. **^{13}C{^{1}H} NMR** (125 MHz, CD_3Cl): δ 173.5, 135.5, 134.1, 121.9, 60.4, 34.1, 29.5, 26.6, 24.4, 18.4, 14.4, –4.8, –5.1 ppm. **^{1}H/^{29}Si HMQC NMR** (500/99 MHz, $CDCl_3$, optimized for J = 7.0 Hz): δ 6.12/–2.3, 5.84/–2.3, 1.82/–2.3, 0.27/–2.3 ppm. **HRMS** (APCI) m/z: [M+H]$^+$ Calcd for $C_{13}H_{24}NO_2Si^+$ 254.1571; Found 254.1565.

5.11. 4-Cyano-4-(dimethyl(vinyl)silyl)butyl benzoate (3ha)

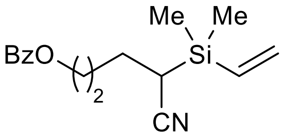

3ha

$C_{16}H_{21}NO_2Si$

M = 287.43 g/mol

Prepared from 4-cyano-4-(((trifluoromethyl)sulfonyl)oxy)butyl benzoate (**1h**, 70.2 mg, 0.20 mmol, 1.0 equiv) with chlorodimethyl(vinyl)silane (**2a**, 72.4 mg, 0.60 mmol, 3.0 equiv) at rt in DMA for 18 h according to **GP2**. Purification by flash column chromatography on silica gel (10:1 cyclohexane/EtOAc) afforded **3ha** as a colorless oil (28.7 mg, 50% yield).
R$_f$ = 0.40 (5/1 cyclohexane/EtOAc). **IR** (ATR): $\tilde{\nu}$ = 3054, 2955, 2219, 1716, 1600, 1451, 1405, 1272, 1114, 1027, 960, 842, 786, 712 cm^{-1}. **^{1}H NMR** (500 MHz, $CDCl_3$): δ 8.02 (d, *J* = 7.7 Hz, 2H), 7.58–7.55 (m, 1H), 7.46–7.45 (m, 2H), 6.17–6.09 (m, 2H), 5.88–5.81 (m, 1H), 4.41–4.31 (m, 2H), 2.19–2.11 (m, 1H), 1.95–1.87 (m, 2H), 1.72–1.67 (m, 2H), 0.29 (s, 6H) ppm. **^{13}C{^{1}H} NMR** (125 MHz, CD_3Cl): δ 166.6, 135.7, 133.9, 133.2, 130.3, 129.7, 128.5, 121.7, 63.8, 29.0, 23.6, 18.1, –4.8, –5.1 ppm. **^{29}Si{^{1}H} DEPT NMR** (99 MHz, $CDCl_3$): δ –1.9 ppm. **HRMS** (APCI) m/z: $[M+H]^+$ Calcd for $C_{16}H_{22}NO_2Si^+$ 288.1414; Found 288.1410.

5.12. 4-Cyano-4-(dimethyl(vinyl)silyl)butyl pivalate (3ia)

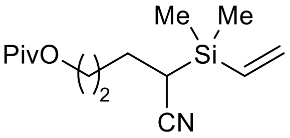

3ia

$C_{14}H_{25}NO_2Si$

M = 267.44 g/mol

Prepared from 4-cyano-4-(((trifluoromethyl)sulfonyl)oxy)butyl pivalate (**1i**, 66.2 mg, 0.20 mmol, 1.0 equiv) with chlorodimethyl(vinyl)silane (**2a**, 72.4 mg, 0.60 mmol, 3.0 equiv) at rt in DMA for 18 h according to **GP2**. Purification by flash column chromatography on silica gel (10:1 cyclohexane/EtOAc) afforded **3ia** as a colorless oil (21.9 mg, 41% yield).
R$_f$ = 0.40 (5/1 cyclohexane/EtOAc). **IR** (ATR): $\tilde{\nu}$ = 2960, 2220, 1726, 1479, 1401, 1284, 1156, 1039, 1010, 960, 842, 785, 710 cm^{-1}. **^{1}H NMR** (500 MHz, $CDCl_3$): δ 6.15–6.08 (m, 2H), 5.88–5.80 (m, 1H), 4.15–4.05 (m, 2H), 2.04–1.95 (m, 1H), 1.88–1.85 (m, 1H), 1.81–1.72 (m, 1H), 1.62–1.57 (m, 2H), 1.19 (s, 9H), 0.28 (s, 6H) ppm. **^{13}C{^{1}H} NMR** (125 MHz, CD_3Cl): δ 178.6, 135.7, 133.9, 121.7, 63.1, 38.9, 28.9, 27.3, 23.5, 18.0, –4.8, –5.2 ppm. **^{29}Si{^{1}H} DEPT NMR** (99 MHz, $CDCl_3$): δ –1.9 ppm. **HRMS** (APCI) m/z: $[M+H]^+$ Calcd for $C_{14}H_{26}NO_2Si^+$ 268.1727; Found 268.1723.

5.13. 2-(Dimethyl(vinyl)silyl)hept-6-enenitrile (3ja)

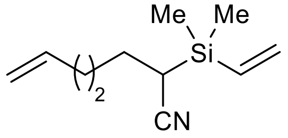

3ja

$C_{11}H_{19}NSi$

M = 193.37 g/mol

Prepared from 1-cyanohex-5-en-1-yl trifluoromethanesulfonate (**1j**, 51.4 mg, 0.20 mmol, 1.0 equiv) with chlorodimethyl(vinyl)silane (**2a**, 72.4 mg, 0.60 mmol, 3.0 equiv) at rt in DMA for 18 h according to **GP2**. Purification by flash column chromatography on silica gel (20:1 cyclohexane/EtOAc) afforded **3ja** as a colorless oil (20.9 mg, 54% yield).
R$_f$ = 0.60 (10/1 cyclohexane/EtOAc). **IR** (ATR): $\tilde{\nu}$ = 3054, 2940, 2860, 2219, 1640, 1594, 1457, 1406, 1254, 1009, 959, 913, 841, 785, 709 cm^{-1}. **^{1}H NMR** (500 MHz, $CDCl_3$): δ 6.15–6.12 (m, 2H), 5.87–5.73 (m, 2H), 5.04–4.97 (m, 2H), 2.15–2.03 (m, 2H), 1.83–1.73 (m, 2H), 1.59–1.49 (m, 3H), 0.27 (s, 6H) ppm. **^{13}C{^{1}H} NMR** (125 MHz,

CD_3Cl): δ 137.9, 135.7, 134.2, 122.1, 115.3, 33.0, 29.1, 26.2, 18.3, –4.7, –5.1 ppm. **$^1H/^{29}Si$ HMQC NMR** (500/99 MHz, $CDCl_3$, optimized for *J* = 7.0 Hz): δ 6.14/–2.4, 5.85/–2.4, 1.81/–2.4, 0.27/–2.4 ppm. **HRMS** (APCI) m/z: $[M+H]^+$ Calcd for $C_{11}H_{20}NSi^+$ 194.1360; Found 194.1360.

5.14. 4-((6-Cyano-6-(dimethyl(vinyl)silyl)hexyl)oxy)benzonitrile (3ka)

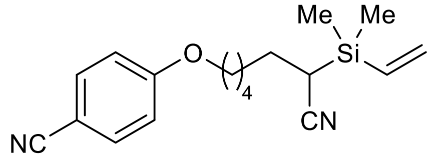

3ka

$C_{18}H_{24}N_2OSi$

M = 312.49 g/mol

Prepared from 1-cyano-6-(4-cyanophenoxy)hexyl trifluoromethanesulfonate (**1k**, 75.2 mg, 0.20 mmol, 1.0 equiv) with chlorodimethyl(vinyl)silane (**2a**, 72.4 mg, 0.60 mmol, 3.0 equiv) at rt in DMA for 18 h according to **GP2**. Purification by flash column chromatography on silica gel (10:1 cyclohexane/EtOAc) afforded **3ka** as a colorless oil (35.6 mg, 57% yield).

R_f = 0.40 (5/1 cyclohexane/EtOAc). **IR** (ATR): $\tilde{\nu}$ = 3051, 2940, 2860, 2222, 1605, 1508, 1470, 1405, 1301, 1257, 1171, 1009, 961, 836, 785, 706 cm^{-1}. **^1H NMR** (500 MHz, $CDCl_3$): δ 7.57 (d, *J* = 8.8 Hz, 2H), 6.92 (d, *J* = 8.8 Hz, 2H), 6.17–6.09 (m, 2H), 5.87–5.81 (m, 1H), 4.00 (t, *J* = 6.3 Hz, 2H), 1.85–1.80 (m, 3H), 1.77–1.69 (m, 1H), 1.63–1.44 (m, 5H), 0.28 (s, 6H) ppm. **$^{13}C\{^1H\}$ NMR** (125 MHz, CD_3Cl): δ 162.5, 135.7, 134.1 (2C), 122.0, 119.4, 115.3, 104.0, 68.2, 29.8, 28.9, 26.8, 25.6, 18.5, –4.7, –5.1 ppm. **$^1H/^{29}Si$ HMQC NMR** (500/99 MHz, $CDCl_3$, optimized for *J* = 7.0 Hz): δ 6.13/–2.4, 5.84/–2.4, 1.82/–2.4, 0.28/–2.4 ppm. **HRMS** (APCI) m/z: $[M+H]^+$ Calcd for $C_{18}H_{25}N_2OSi^+$ 313.1731; Found 313.1729.

5.15. 2-(Cyclohex-2-en-1-yl)-2-(dimethyl(vinyl)silyl)acetonitrile (3la)

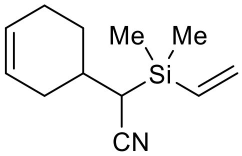

3la

$C_{12}H_{19}NSi$

M = 205.38 g/mol

Prepared from cyano(cyclohex-2-en-1-yl)methyl trifluoromethanesulfonate (**1l**, 53.8 mg, 0.20 mmol, 1.0 equiv) with chlorodimethyl(vinyl)silane (**2a**, 72.4 mg, 0.60 mmol, 3.0 equiv) at rt in DMA for 18 h according to **GP2**. Purification by flash column chromatography on silica gel (20:1 cyclohexane/EtOAc) afforded **3la** as a colorless oil (26.3 mg, 64% yield, d.r.=1:1).

R_f = 0.50 (10/1 cyclohexane/EtOAc). **IR** (ATR): $\tilde{\nu}$ = 3025, 2919, 2839, 2218, 1693, 1593, 1436, 1406, 1254, 1129, 1066, 1046, 1009, 958, 897, 841, 785, 709, 685 cm^{-1}. **^1H NMR** (500 MHz, $CDCl_3$): δ 6.22–6.10 (m, 2H), 5.87–5.82 (m, 1H), 5.73–5.60 (m, 2H), 2.17–2.01 (m, 4H), 1.93–1.85 (m, 2H), 1.70–1.50 (m, 2H), 0.32–0.31 (m, 6H) ppm. **$^{13}C\{^1H\}$ NMR** (125 MHz, CD_3Cl): δ 135.1, 135.0, 127.1, 126.7, 125.7, 125.6, 120.9, 120.7, 32.9, 32.44, 32.35, 30.4, 30.3, 27.8, 26.0, 25.4, 25.3, –3.5, –3.6, –3.8, –3.9 ppm. **$^{29}Si\{^1H\}$ DEPT NMR** (99 MHz, $CDCl_3$): δ –3.1 ppm. **HRMS** (APCI) m/z: $[M+H]^+$ Calcd for $C_{12}H_{20}NSi^+$ 206.1360; Found 206.1357.

5.16. 2-(Dimethyl(vinyl)silyl)octanenitrile (3ma)

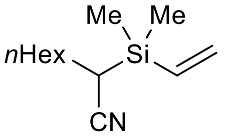

3ma

$C_{12}H_{23}NSi$

M = 209.41 g/mol

Prepared from 1-cyanoheptyl trifluoromethanesulfonate (**1m**, 75.2 mg, 0.20 mmol, 1.0 equiv) with chlorodimethyl(vinyl)silane (**2a**, 72.4 mg, 0.60 mmol, 3.0 equiv) at rt in DMA for 18 h according to **GP2**. Purification by flash column chromatography on silica gel (20:1 cyclohexane/EtOAc) afforded **3ma** as a colorless oil (15.0 mg, 36% yield).

R$_f$ = 0.60 (10/1 cyclohexane/EtOAc). **IR** (ATR): $\tilde{\nu}$ = 2930, 2857, 2220, 1458, 1404, 1256, 1088, 1011, 960, 818, 711 cm^{-1}. **^{1}H NMR** (500 MHz, $CDCl_3$): δ 6.18–6.11 (m, 2H), 5.87–5.80 (m, 1H), 1.82–1.79 (m, 1H), 1.68–1.60 (m, 1H), 1.53–1.25 (m, 9H), 0.89 (t, *J* = 7.1 Hz, 3H), 0.270 (s, 3H), 0.269 (s, 3H) ppm. **^{13}C{^{1}H} NMR** (125 MHz, CD_3Cl): δ 135.4, 134.3, 122.2, 31.7, 30.0, 28.8, 26.9, 22.7, 18.4, 14.2, –4.7, –5.1 ppm. **^{1}H/^{29}Si HMQC NMR** (500/99 MHz, $CDCl_3$, optimized for *J* = 7.0 Hz): δ 6.14/–2.5, 5.84/–2.5, 1.80/–2.5, 0.27/–2.5 ppm. **HRMS** (APCI) m/z: [M+H]$^+$ Calcd for $C_{12}H_{24}NSi^+$ 210.1673; Found 210.1669.

5.17. 2-Cyclopentyl-2-(dimethyl(vinyl)silyl)acetonitrile (3na)

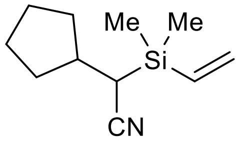

3na

$C_{11}H_{19}NSi$

M = 193.37 g/mol

Prepared from cyano(cyclopentyl)methyl trifluoromethanesulfonate (**1n**, 51.4 mg, 0.20 mmol, 1.0 equiv) with chlorodimethyl(vinyl)silane (**2a**, 72.4 mg, 0.60 mmol, 3.0 equiv) at rt in DMA for 18 h according to **GP2**. Purification by flash column chromatography on silica gel (20:1 cyclohexane/EtOAc) afforded **3na** as a colorless oil (15.5 mg, 40% yield).

R$_f$ = 0.60 (10/1 cyclohexane/EtOAc). **IR** (ATR): $\tilde{\nu}$ = 2954, 2869, 2220, 1736, 1452, 1406, 1254, 1075, 1009, 958, 841, 785, 702 cm^{-1}. **^{1}H NMR** (500 MHz, $CDCl_3$): δ 6.20–6.09 (m, 2H), 5.85–5.80 (m, 1H), 2.06–2.02 (m, 1H), 1.98 (d, *J* = 5.5 Hz, 1H), 1.89–1.78 (m, 2H), 1.76–1.66 (m, 2H), 1.61–1.49 (m, 2H), 1.44–1.33 (m, 2H), 0.29 (s, 6H) ppm. **^{13}C{^{1}H} NMR** (125 MHz, CD_3Cl): δ 135.1, 134.9, 121.3, 37.9, 34.1, 31.8, 25.3, 25.2, 24.2, –3.8, –4.1 ppm. **^{1}H/^{29}Si HMQC NMR** (500/99 MHz, $CDCl_3$, optimized for *J* = 7.0 Hz): δ 6.17/–3.1, 5.83/–3.1, 1.98/–3.1, 0.29/–3.1 ppm. **HRMS** (APCI) m/z: [M+H]$^+$ Calcd for $C_{11}H_{20}NSi^+$ 194.1360; Found 194.1358.

5.18. 2-Cyclohexyl-2-(dimethyl(vinyl)silyl)acetonitrile (3oa)

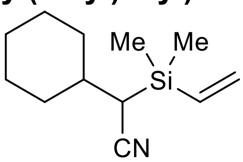

3oa

$C_{12}H_{21}NSi$

M = 207.39 g/mol

Prepared from cyano(cyclohexyl)methyl trifluoromethanesulfonate (**1o**, 54.2 mg, 0.20 mmol, 1.0 equiv) with chlorodimethyl(vinyl)silane (**2a**, 72.4 mg, 0.60 mmol, 3.0 equiv) at rt in DMA for 18 h according to **GP2**. Purification by flash column chromatography on silica gel (20:1 cyclohexane/EtOAc) afforded **3oa** as a colorless oil (30.3 mg, 73% yield).

R$_f$ = 0.60 (10/1 cyclohexane/EtOAc). **IR** (ATR): $\tilde{\nu}$ = 2926, 2853, 2217, 1449, 1406, 1254, 1006, 956, 892, 820, 788, 699 cm^{-1}. **^{1}H NMR** (500 MHz, $CDCl_3$): δ 6.20–6.08 (m, 2H), 5.85–5.80 (m, 1H), 1.82–1.80 (m, 2H), 1.77–1.71 (m, 2H), 1.66–1.63 (m, 2H), 1.61–1.54 (m, 1H), 1.36–1.11 (m, 5H), 0.29 (s, 3H), 0.28 (s, 3H) ppm. **^{13}C{^{1}H} NMR** (125 MHz, CD_3Cl): δ 135.2, 134.8, 121.0, 36.2, 34.6, 31.7, 26.43, 26.38, 26.3, 25.7, –3.5, –3.8 ppm. **^{29}Si{^{1}H} DEPT NMR** (99 MHz, $CDCl_3$): δ –3.2 ppm. **HRMS** (APCI) m/z: $[M+H]^+$ Calcd for $C_{12}H_{22}NSi^+$ 208.1516; Found 208.1513.

5.19. 2-(Dimethyl(vinyl)silyl)-2-(tetrahydro-2*H*-pyran-4-yl)acetonitrile (3pa)

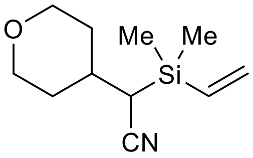

3pa

$C_{11}H_{19}NOSi$

M = 209.36 g/mol

Prepared from cyano(tetrahydro-2*H*-pyran-4-yl)methyl trifluoromethanesulfonate (**1p**, 54.6 mg, 0.20 mmol, 1.0 equiv) with chlorodimethyl(vinyl)silane (**2a**, 72.4 mg, 0.60 mmol, 3.0 equiv) at rt in DMA for 18 h according to **GP2**. Purification by flash column chromatography on silica gel (10:1 cyclohexane/EtOAc) afforded **3pa** as a colorless oil (26.7 mg, 64% yield).

R$_f$ = 0.30 (10/1 cyclohexane/EtOAc). **IR** (ATR): $\tilde{\nu}$ = 2952, 2843, 2218, 1593, 1443, 1406, 1253, 1156, 1100, 1012, 959, 822, 785, 705 cm^{-1}. **^{1}H NMR** (500 MHz, $CDCl_3$): δ 6.20–6.10 (m, 2H), 5.85–5.80 (m, 1H), 3.97 (td, J_1 = 12.8 Hz, J_2 = 4.3 Hz, 2H), 3.35 (td, J_1 = 11.9 Hz, J_2 = 2.2 Hz, 2H), 1.86–1.78 (m, 2H), 1.74–1.71 (m, 1H), 1.67–1.59 (m, 2H), 1.56–1.53 (m, 1H), 0.32 (s, 3H), 0.31 (s, 3H) ppm. **^{13}C{^{1}H} NMR** (125 MHz, CD_3Cl): δ 135.3, 134.8, 120.3, 67.74, 67.71, 33.9, 33.8, 32.0, 26.0, –3.5, –3.9 ppm. **^{29}Si{^{1}H} DEPT NMR** (99 MHz, $CDCl_3$): δ –3.2 ppm. **HRMS** (APCI) m/z: $[M+H]^+$ Calcd for $C_{11}H_{20}NOSi^+$ 210.1309; Found 210.1308.

5.20. *tert*-Butyl 4-(cyano(dimethyl(vinyl)silyl)methyl)piperidine-1-carboxylate (3qa)

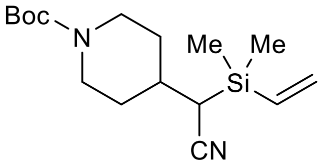

3qa

$C_{16}H_{28}N_2O_2Si$

M = 308.50 g/mol

Prepared from *tert*-butyl 4-(cyano(((trifluoromethyl)sulfonyl)oxy)methyl)piperidine-1-carboxylate (**1q**, 74.4 mg, 0.20 mmol, 1.0 equiv) with chlorodimethyl(vinyl)silane (**2a**, 72.4 mg, 0.60 mmol, 3.0 equiv) at rt in DMA for 18 h according to **GP2**. Purification by flash column chromatography on silica gel (10:1 cyclohexane/EtOAc) afforded **3qa** as a colorless oil (24.3 mg, 40% yield).

R$_f$ = 0.40 (5/1 cyclohexane/EtOAc). **IR** (ATR): $\tilde{\nu}$ = 2972, 2933, 2855, 2218, 1690, 1422, 1365, 1252, 1170, 1130, 1084, 1009, 976, 841, 784, 705 cm^{-1}. **^{1}H NMR** (500 MHz,

$CDCl_3$): δ 6.20–6.10 (m, 2H), 5.89–5.80 (m, 1H), 4.14 (br s, 2H), 2.65 (br s, 2H), 1.85 (d, J = 4.9 Hz, 1H), 1.80–1.75 (m, 1H), 1.74–1.68 (m, 1H), 1.61–1.57 (m, 1H), 1.50–1.47 (m, 1H), 1.45 (s, 9H), 1.43–1.38 (m, 1H), 0.31 (s, 3H), 0.30 (s, 3H) ppm. **^{13}C{^{1}H} NMR** (125 MHz, CD_3Cl): δ 154.7, 135.4, 134.7, 120.3, 79.8, 43.6, 34.8, 33.2, 31.0, 28.6, 25.7, –3.5, –3.8 ppm. **^{29}Si{^{1}H} DEPT NMR** (99 MHz, $CDCl_3$): δ –3.0 ppm. **HRMS** (APCI) m/z: [M+H]$^+$ Calcd for $C_{16}H_{29}N_2O_2Si^+$ 309.1993; Found 309.1987.

5.21. 2-(Dimethyl(vinyl)silyl)-3,3-dimethylbutanenitrile (3ra)

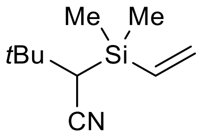

3ra

$C_{10}H_{19}NSi$

M =181.35 g/mol

Prepared from 1-cyano-2,2-dimethylpropyl trifluoromethanesulfonate (**1r**, 49.0 mg, 0.20 mmol, 1.0 equiv) with chlorodimethyl(vinyl)silane (**2a**, 72.4 mg, 0.60 mmol, 3.0 equiv) at rt in DMA for 18 h according to **GP2**. Purification by flash column chromatography on silica gel (20:1 cyclohexane/EtOAc) afforded **3ra** as a colorless oil (25.3 mg, 70% yield).

R_f = 0.60 (10/1 cyclohexane/EtOAc). **IR** (ATR): $\tilde{\nu}$ = 2961, 2925, 2853, 2226, 1542, 1460, 1260, 1094, 1027, 800 cm^{-1}. **^{1}H NMR** (500 MHz, $CDCl_3$): δ 6.27–6.20 (m, 1H), 6.10–6.06 (m, 1H), 5.84–5.80 (m, 1H), 1.87 (s, 1H), 1.12 (s, 9H), 0.35 (s, 3H), 0.33 (s, 3H) ppm. **^{13}C{^{1}H} NMR** (125 MHz, CD_3Cl): δ 136.4, 134.2, 121.5, 33.9, 33.4, 30.6, –2.0, –2.3 ppm. **^{1}H/^{29}Si HMQC NMR** (500/99 MHz, $CDCl_3$, optimized for J = 7.0 Hz): δ 6.23/–4.7, 5.82/–4.7, 1.87/–4.7, 0.34/–4.7 ppm. **HRMS** (APCI) m/z: [M+H]$^+$ Calcd for $C_{10}H_{20}NSi^+$ 182.1360; Found 182.1360.

6. Synthesis of 3aa on 1.0-mmol Scale

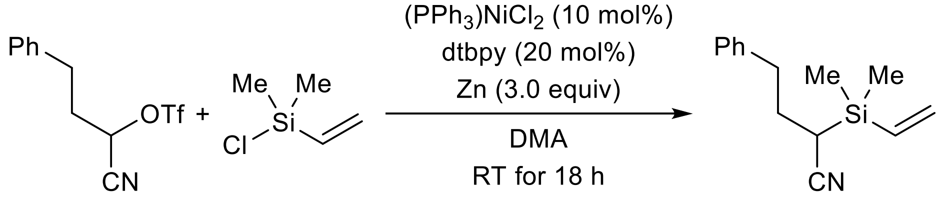

1a **2a** (3.0 equiv) **3aa**

To an oven-dried 50-mL Schlenk flask equipped with a magnetic stir bar were subsequently added $(Ph_3P)_2NiCl_2$ (65.5 mg, 0.10 mmol, 0.10 equiv), dtbpy (53.5 mg, 0.20 mmol, 0.20 equiv), and Zn (196 mg, 3.0 mol, 3.0 equiv). The tube was placed under vacuum and backfilled with N_2 (3 times). A solution of substrate **1a** (293 mg, 1.0 mmol, 1.0 equiv) and chlorosilane **2a** (362 mg, 3.0 mmol, 3.0 equiv) in DMA (15 mL) was added by syringe, and the mixture was maintained with stirring at room temperature for 18 h. After the indicated reaction time, the mixture was diluted with EtOAc (100 mL) and washed with water. The organic phase was then dried over anhydrous $MgSO_4$, filtered, and concentrated *in vacuo*. The crude residue was purified by column chromatography on silica gel (20/1 cyclohexane/EtOAc), and product **3aa** was obtained as a colorless oil (114 mg, 50%).

7. Mechanistic Experiments

radical-trapping experiment

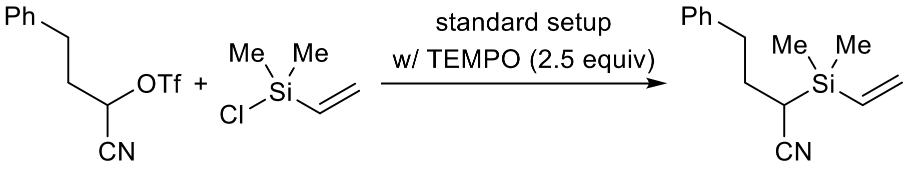

1a **2a** **3aa**: 75% (GLC)

To an oven-dried 20-mL Schlenk tube equipped with a magnetic stir bar were subsequently added $(Ph_3P)_2NiCl_2$ (13.1 mg, 0.020 mmol, 0.10 equiv), dtbpy (10.7 mg, 0.040 mmol, 0.20 equiv), Zn (39.2 mg, 0.60 mol, 3.0 equiv), and TEMPO (78.1 mg, 0.50 mmol, 2.5 equiv). The tube was placed under vacuum and backfilled with N_2 (3 times). A solution of substrate **1a** (58.7 mg, 0.20 mmol, 1.0 equiv) and chlorosilane **2a** (72.4 mg, 0.60 mmol, 3.0 equiv) in DMA (1.5 mL) was then added by syringe, and the mixture was maintained with stirring at room temperature for 18 h. After the indicated reaction time, the mixture was diluted with EtOAc (10 mL) and washed with water. The organic phase was then dried over anhydrous $MgSO_4$, filtered, and concentrated *in vacuo*. The residue was dissolved in *tert*-butyl methyl ether (1 mL) and subjected to GLC analysis with tetracosane as the internal standard. The product **3aa** was obtained in 75% GLC yield.

radical-probe experiment

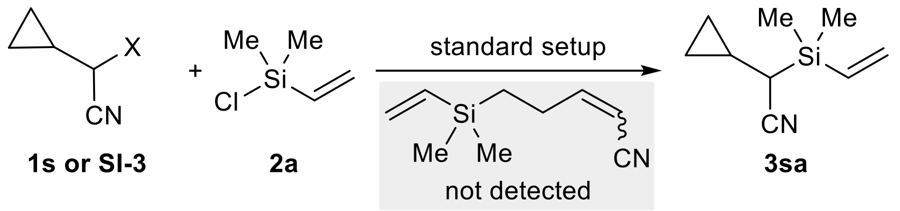

1s or SI-3 **2a** **3sa**

To an oven-dried 20-mL Schlenk tube equipped with a magnetic stir bar were subsequently added $(Ph_3P)_2NiCl_2$ (13.1 mg, 0.020 mmol, 0.10 equiv), dtbpy (10.7 mg, 0.040 mmol, 0.20 equiv), and Zn (39.2 mg, 0.60 mol, 3.0 equiv). The tube was placed under vacuum and backfilled with N_2 (3 times). A solution of substrate **1s** (45.8 mg, 0.20 mmol, 1.0 equiv) or 2-bromo-2-cyclopropylacetonitrile **SI-3** (32.0 mg, 0.20 mmol, 1.0 equiv) and chlorosilane **2a** (72.4 mg, 0.60 mmol, 3.0 equiv) in DMA (1.5 mL) was added by syringe, and the mixture was maintained with stirring at room temperature for 18 h. After the indicated reaction time, the mixture was diluted with EtOAc (10 mL) and washed with water. The organic phase was then dried over anhydrous $MgSO_4$, filtered, and concentrated *in vacuo*. The residue was dissolved in *tert*-butyl methyl ether (1 mL) and subjected to GLC analysis with tetracosane as the internal standard. Product **3sa** was obtained in 10% GLC yield from **1s** and 21% isolated yield from **SI-3**.

7.1. 2-Cyclopropyl-2-(dimethyl(vinyl)silyl)acetonitrile (3sa)

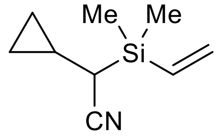

3sa

$C_9H_{15}NSi$

M = 165.31 g/mol

Prepared from 2-bromo-2-cyclopropylacetonitrile (**SI-3**, 32.0 mg, 0.20 mmol, 1.0 equiv) with chlorodimethyl(vinyl)silane (**2a**, 72.4 mg, 0.60 mmol, 3.0 equiv) at rt in DMA for 18 h according to **GP2**. Purification by flash column chromatography on silica gel (20:1 cyclohexane/EtOAc) afforded **3sa** as a colorless oil (6.9 mg, 21% yield).

R$_f$ = 0.50 (10/1 cyclohexane/EtOAc). **IR** (ATR): $\tilde{\nu}$ = 2924, 2854, 2360, 2221, 1541, 1457, 1258, 1050, 799, 720 cm^{-1}. **^{1}H NMR** (500 MHz, $CDCl_3$): δ 6.24–6.08 (m, 2H), 5.89–5.84 (m, 1H), 1.63 (d, J = 7.5 Hz, 1H), 0.95–0.85 (m, 1H), 0.65–0.58 (m, 2H), 0.44–0.38 (m, 1H), 0.32 (s, 6H), 0.30–0.26 (m, 1H) ppm. **^{13}C{^{1}H} NMR** (125 MHz, CD_3Cl): δ 135.2, 134.5, 120.8, 22.8, 8.0, 5.6, 4.6, –4.6, –4.7 ppm. **^{1}H/^{29}Si HMQC NMR** (500/99 MHz, $CDCl_3$, optimized for J = 7.0 Hz): δ 6.16/–2.1, 5.86/–2.1, 1.63/–2.1, 0.32/–2.1 ppm. **HRMS** (APCI) m/z: [M+H]$^+$ Calcd for $C_9H_{16}NSi^+$ 166.1047; Found 166.1043.

racemization experiment

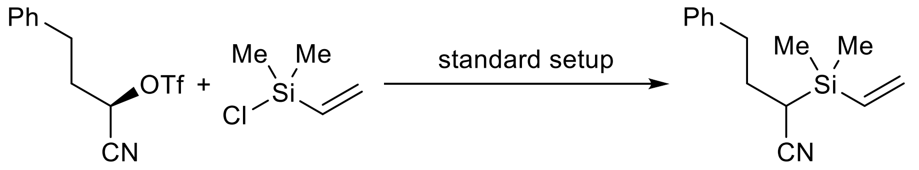

(*R*)-**1a**: 99% ee **2a** *rac*-**3aa**: 0% ee, 68%

To an oven-dried 20-mL Schlenk tube equipped with a magnetic stir bar were subsequently added $(Ph_3P)_2NiCl_2$ (13.1 mg, 0.020 mmol, 0.10 equiv), dtbpy (10.7 mg, 0.040 mmol, 0.20 equiv), Zn (39.2 mg, 0.60 mol, 3.0 equiv). The tube was placed under vacuum and backfilled with N_2 (3 times). A solution of substrate (*R*)-**1a** (58.7 mg, 0.20 mmol, 1.0 equiv, 99% ee) and chlorosilane **2a** (72.4 mg, 0.60 mmol, 3.0 equiv) in DMA (1.5 mL) was added by syringe, and the mixture was maintained with stirring at room temperature for 18 h. After the indicated reaction time, the mixture was diluted with EtOAc (10 mL) and washed with water. The organic phase was then dried over anhydrous $MgSO_4$, filtered, and concentrated *in vacuo*. The crude residue was purified by column chromatography on silica gel (20/1 cyclohexane/EtOAc), and product **3aa** was obtained as a colorless oil (31.2 mg, 68%, 0% ee). The enantiomeric excess of **3aa** was determined by HPLC analysis on a chiral stationary phase (Daicel Chiralcel IC column, column temperature 20 °C, solvent *n*-heptane:*i*PrOH = 99:1, flow rate 0.8 mL/min): t_1 = 16.2 min, t_2 = 17.0 min.

8. NMR Spectra

Figure S1. ^{1}**H NMR** (500 MHz, $CDCl_3$, 298K) of **1-Cyano-3-(5-methylfuran-2-yl)propyl trifluoromethanesulfonate (1d)**

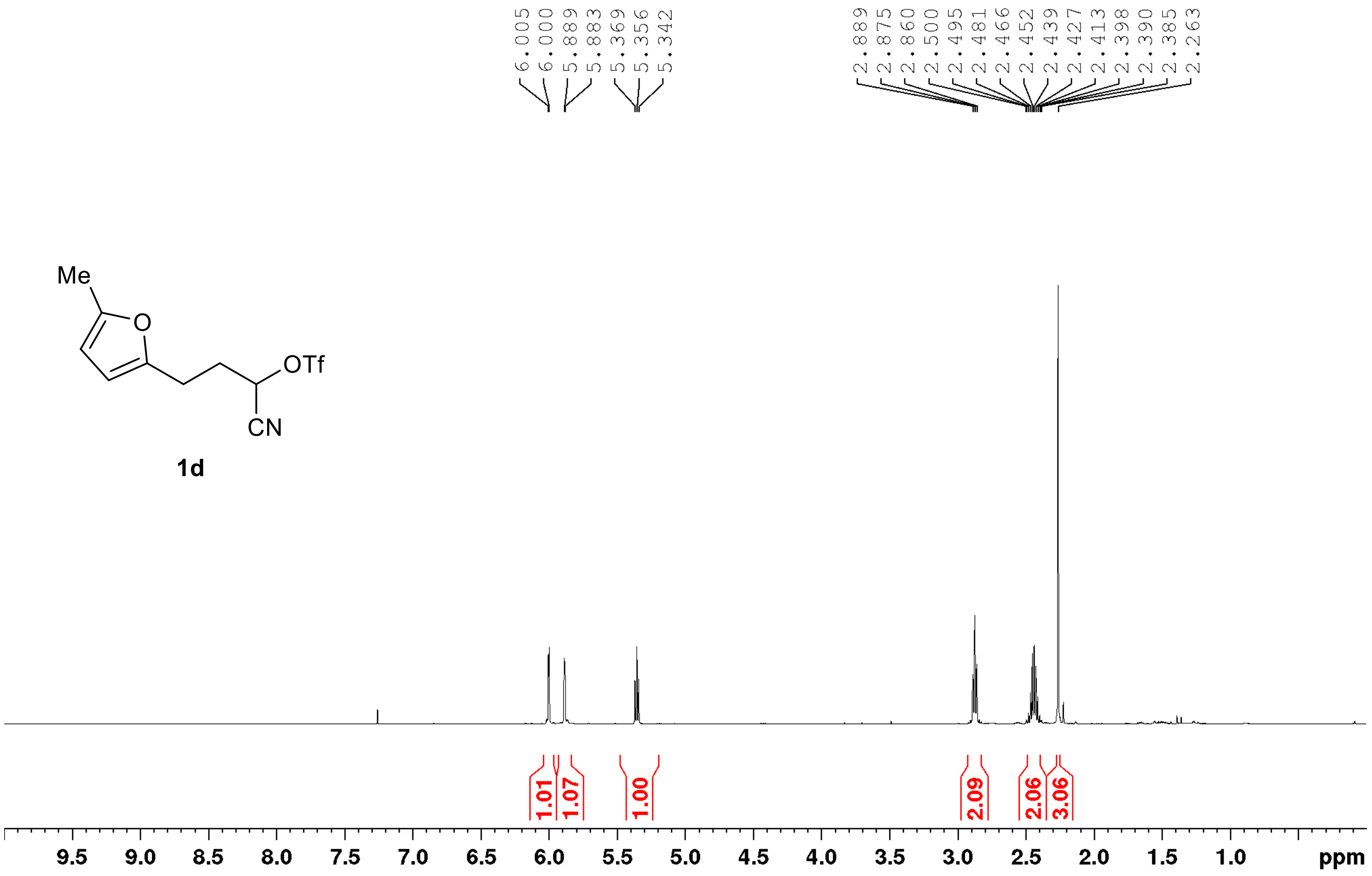

Figure S2. $^{13}C\{^{1}H\}$ **NMR** (125 MHz, $CDCl_3$, 298K) of **1-Cyano-3-(5-methylfuran-2-yl)propyl trifluoromethanesulfonate (1d)**

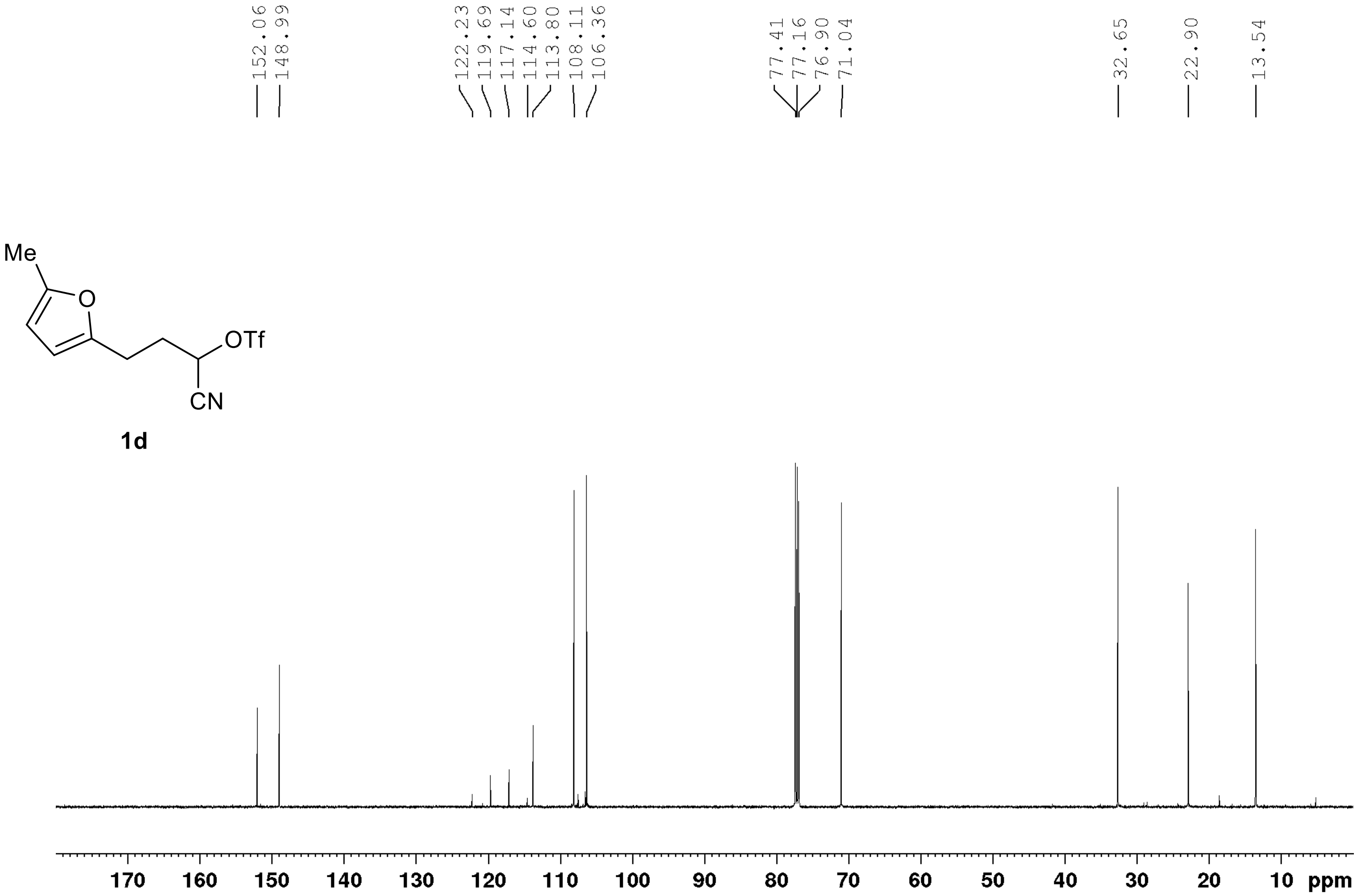

1d

Figure S3. $^{19}F\{^{1}H\}$ NMR (471 MHz, $CDCl_3$) of **1-Cyano-3-(5-methylfuran-2-yl)propyl trifluoromethanesulfonate (1d)**

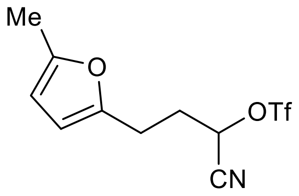

1d

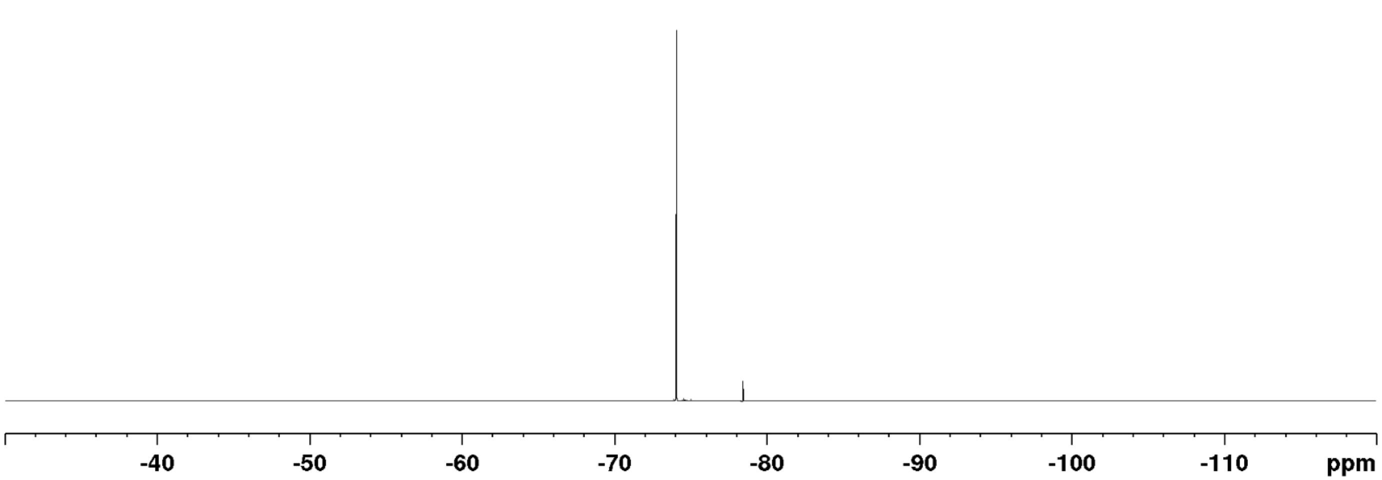

Figure S4. **^{1}H NMR** (500 MHz, $CDCl_3$, 298K) of **1-Cyano-5-phenylpentyl trifluoromethanesulfonate (1e)**

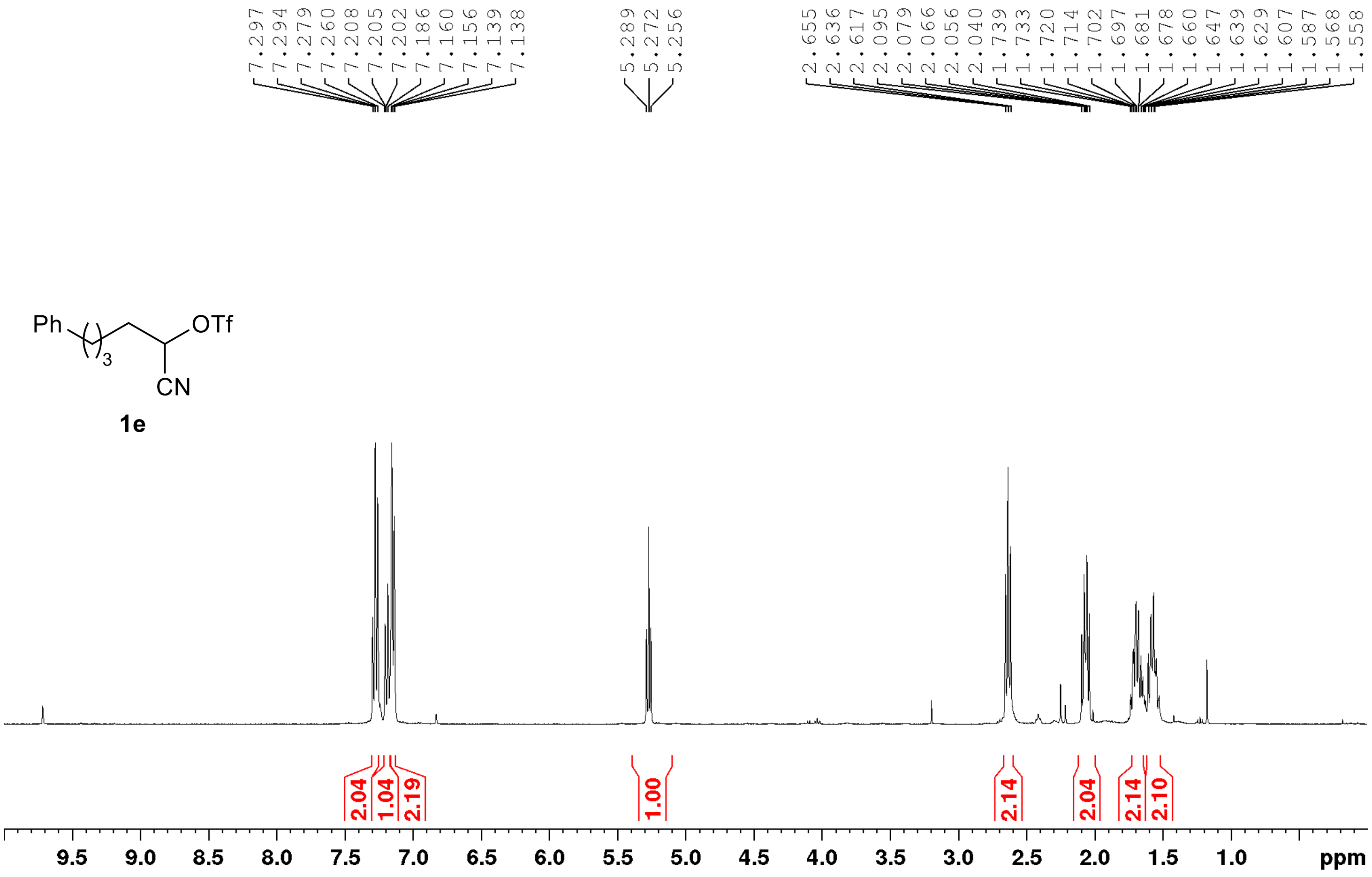

Figure S5. **$^{13}C\{^{1}H\}$ NMR** (125 MHz, $CDCl_3$, 298K) of **1-Cyano-5-phenylpentyl trifluoromethanesulfonate (1e)**

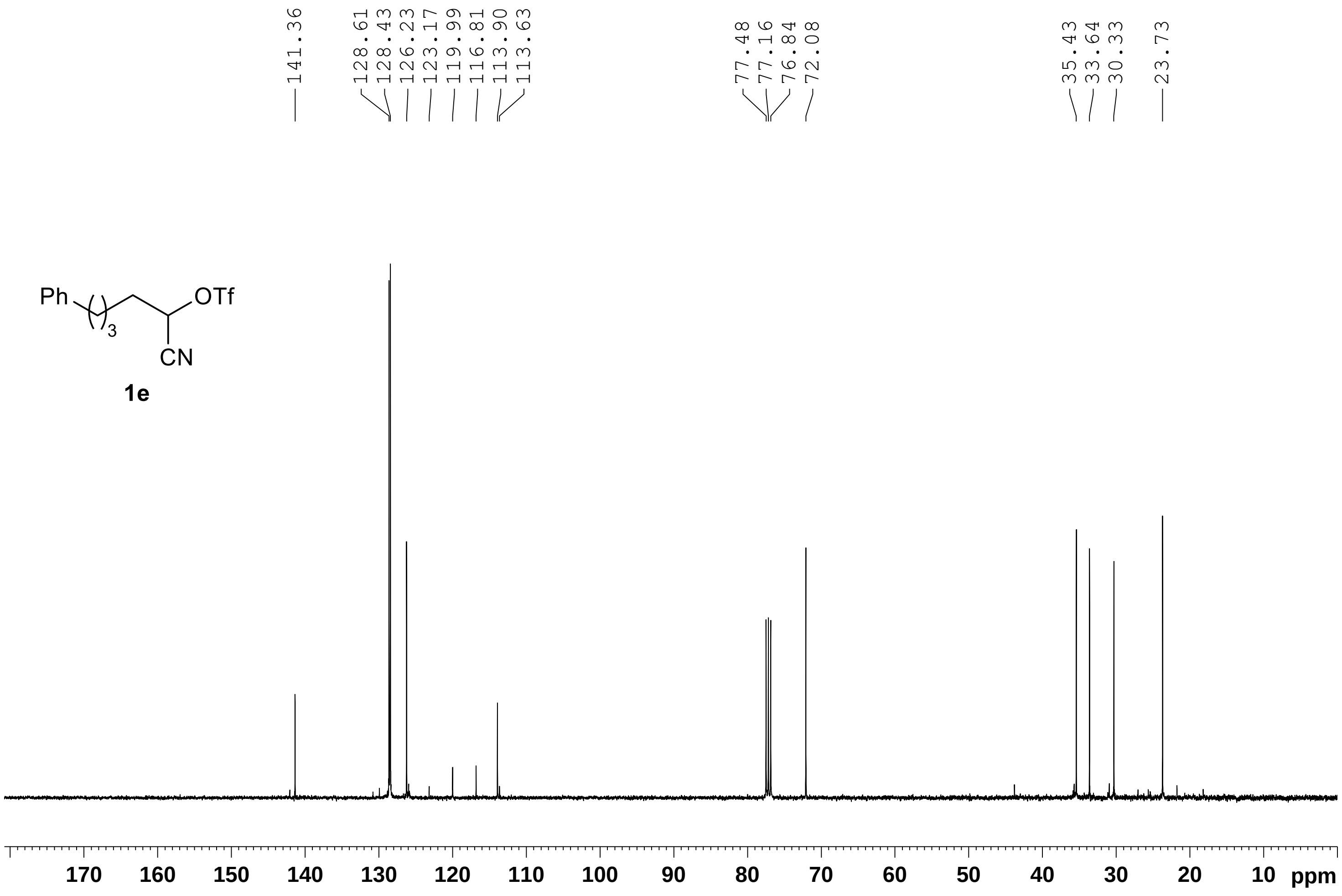

1e

Figure S6. ^{19}F{^{1}H} NMR (471 MHz, $CDCl_3$) of **1-Cyano-5-phenylpentyl trifluoromethanesulfonate (1e)**

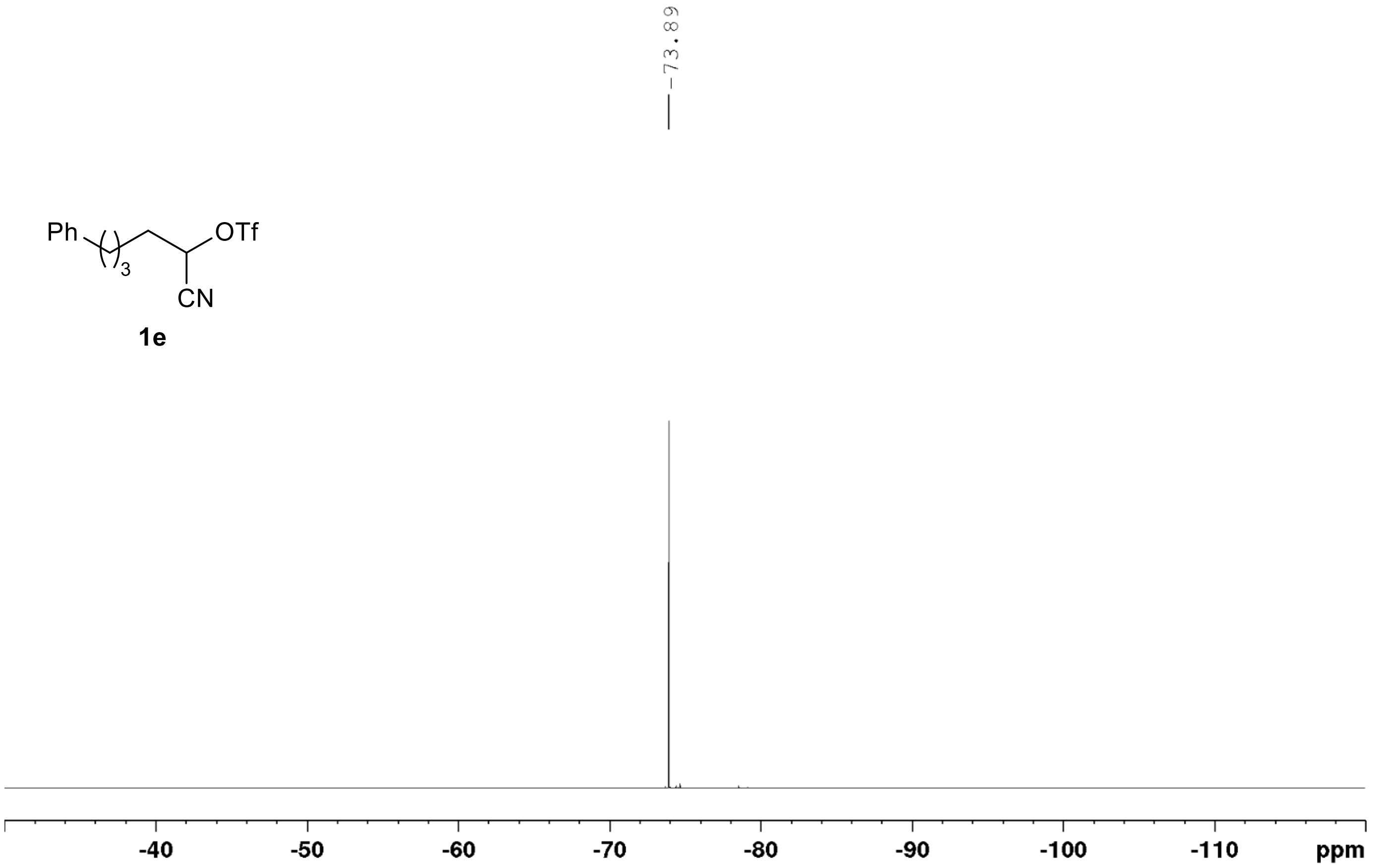

Figure S7. ^{1}H NMR (500 MHz, $CDCl_3$, 298K) of **1-Cyanoheptyl trifluoromethanesulfonate (1m)**

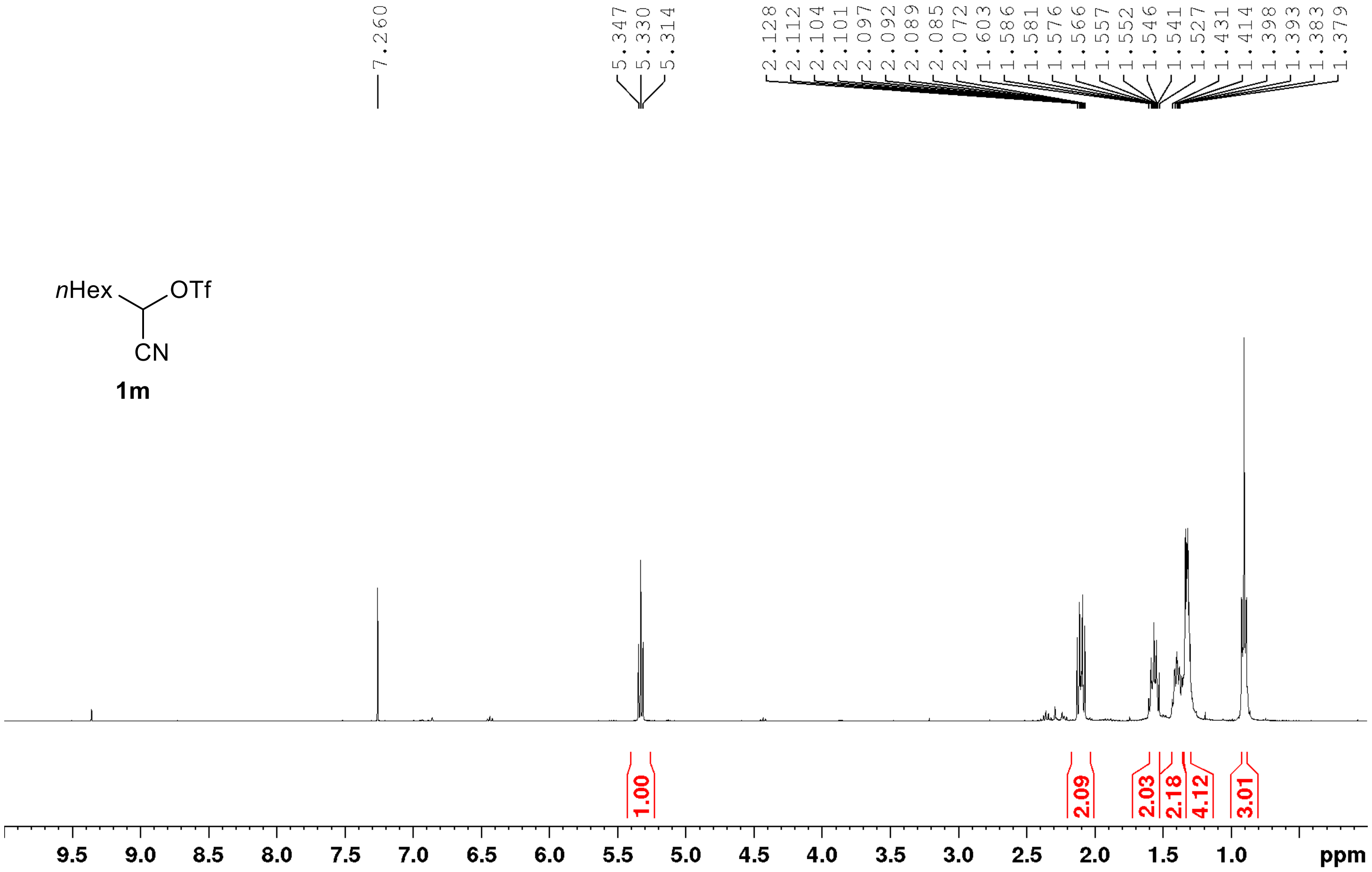

Figure S8. $^{13}C\{^1H\}$ **NMR** (125 MHz, $CDCl_3$, 298K) of **1-Cyanoheptyl trifluoromethanesulfonate (1m)**

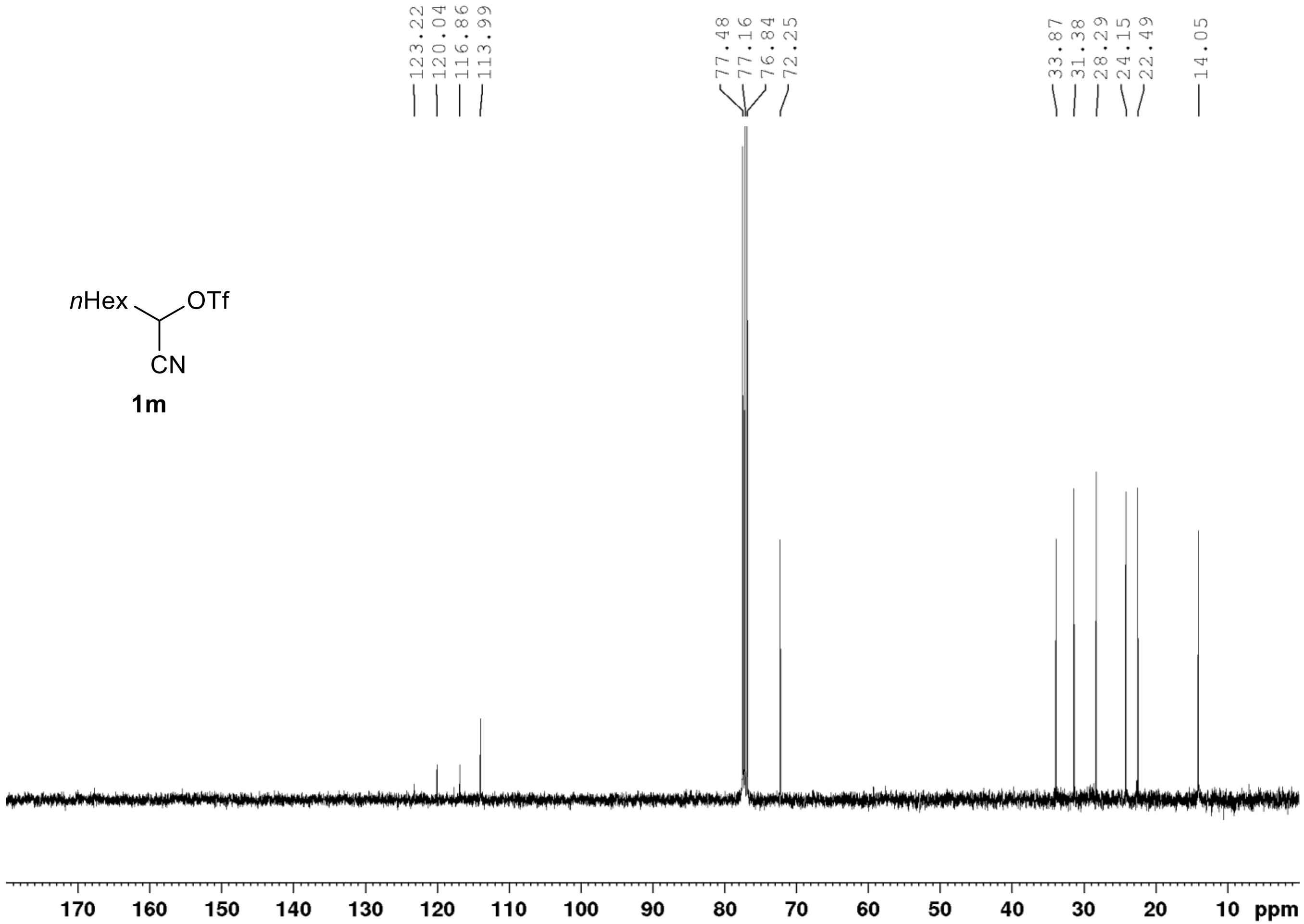

Figure S9. $^{19}F\{^{1}H\}$ NMR (471 MHz, $CDCl_3$) of of **1-Cyanoheptyl trifluoromethanesulfonate (1m)**

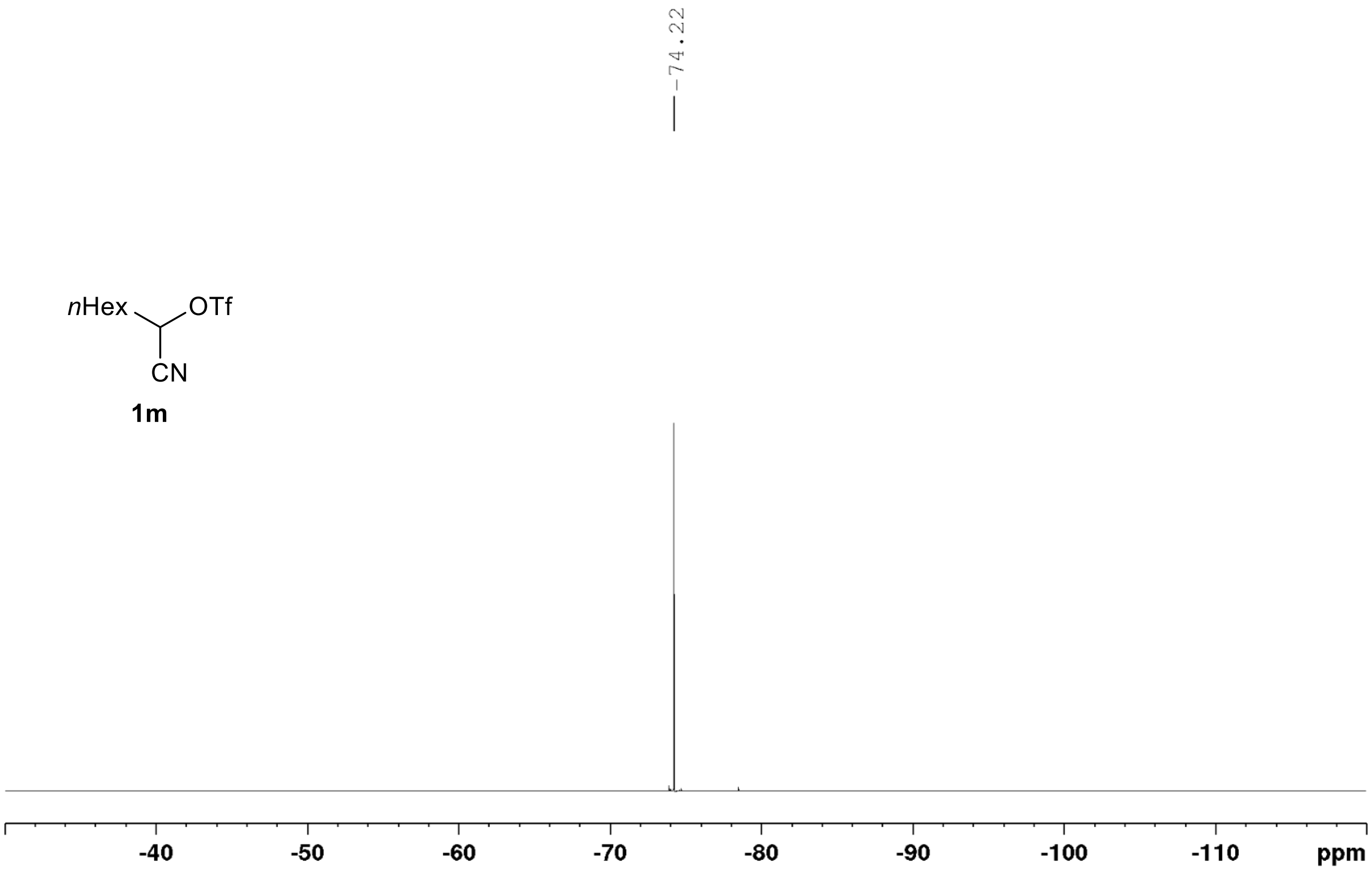

Figure S10. ^{1}H NMR (500 MHz, $CDCl_3$, 298K) of **Cyano(cyclopentyl)methyl trifluoromethanesulfonate (1n)**

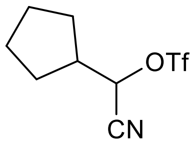

1n

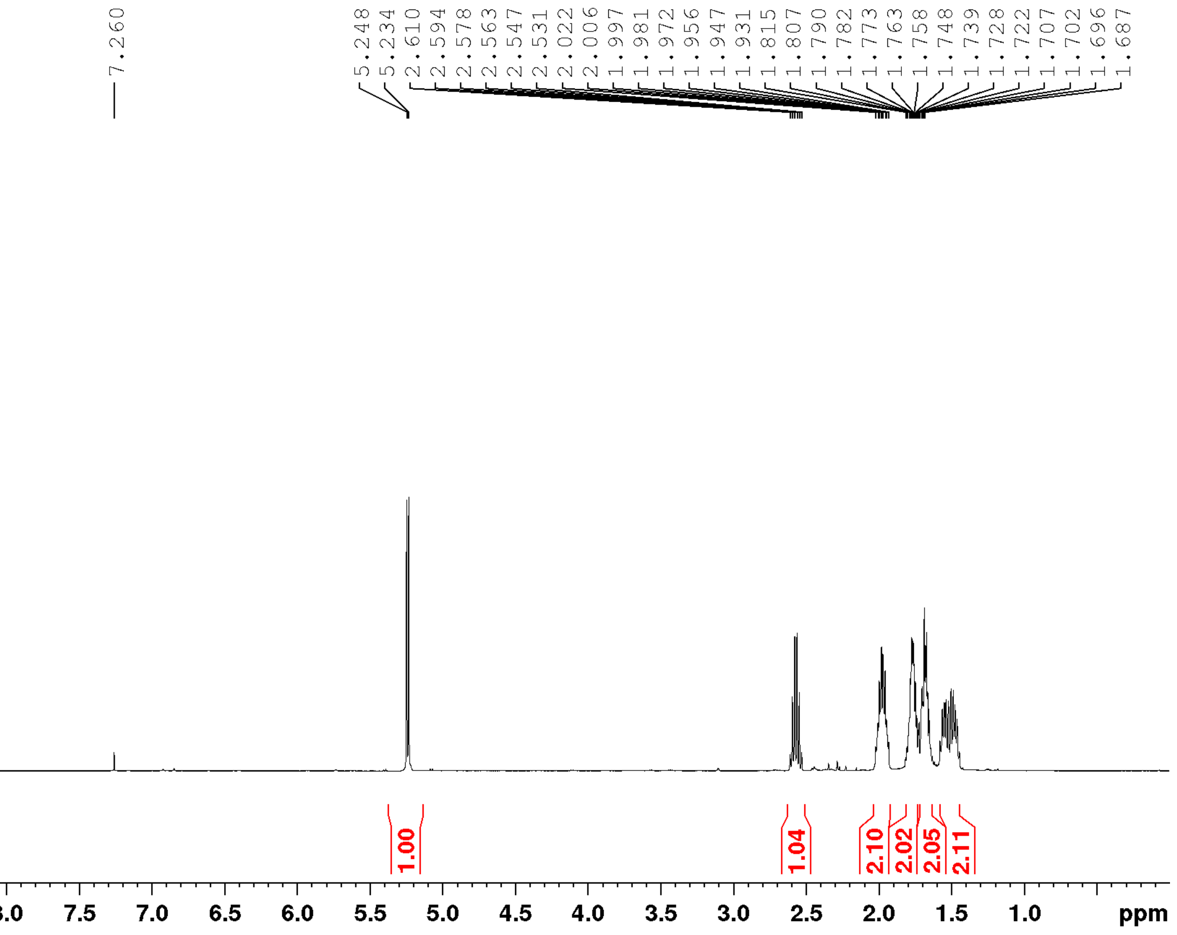

Figure S11. ^{13}**C{**^{1}**H} NMR** (125 MHz, $CDCl_3$, 298K) of **Cyano(cyclopentyl)methyl trifluoromethanesulfonate (1n)**

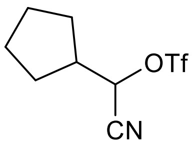

1n

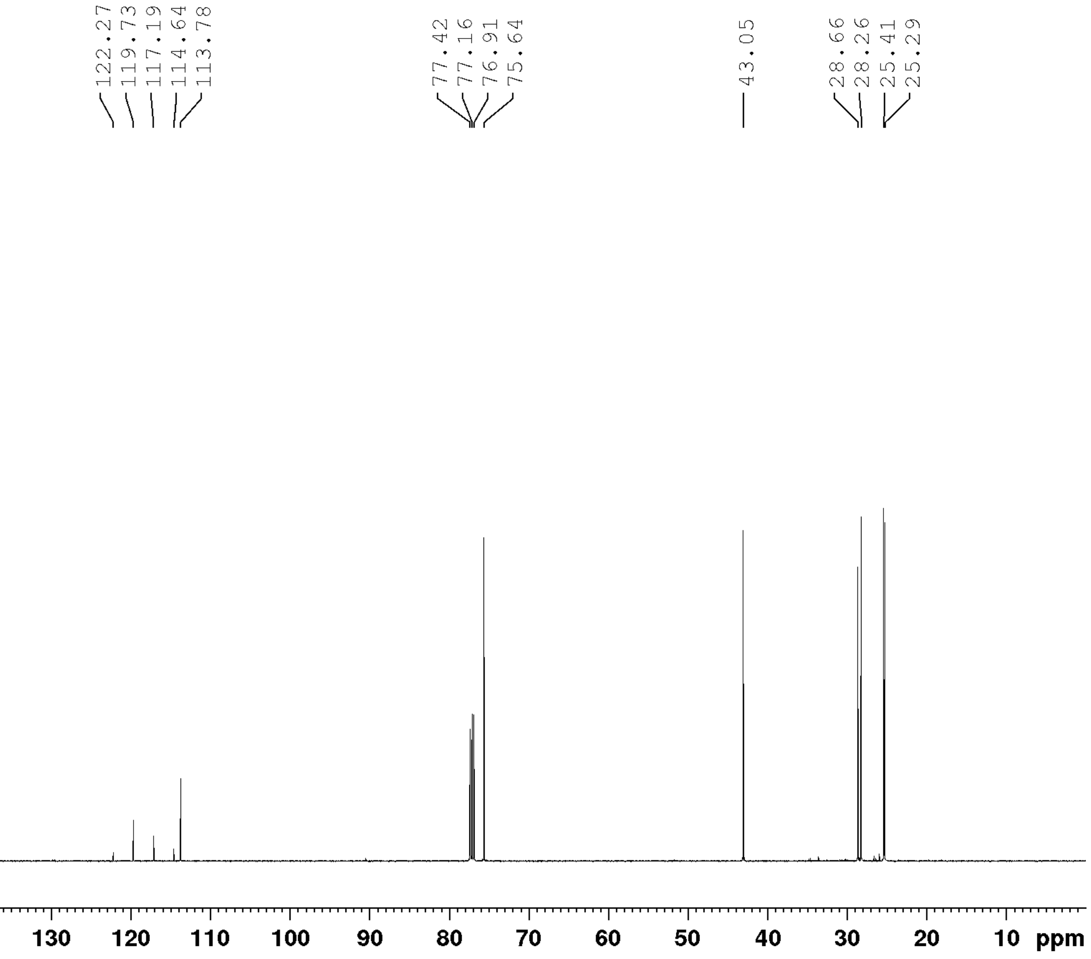

Figure S12. ^{19}F{^{1}H} NMR (471 MHz, $CDCl_3$) of of **Cyano(cyclopentyl)methyl trifluoromethanesulfonate (1n)**

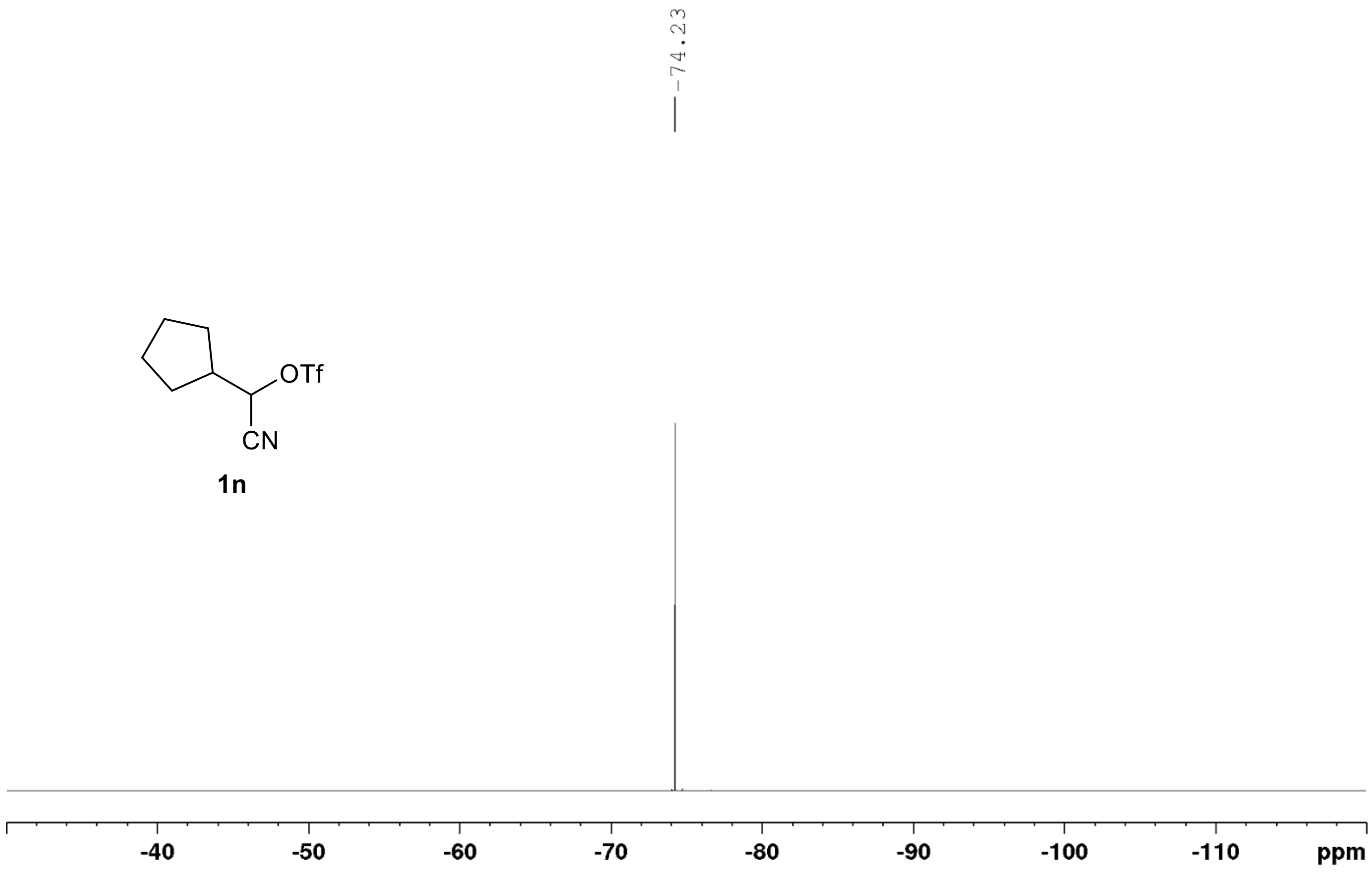

1n

Figure S13. ^{1}H NMR (500 MHz, $CDCl_3$, 298K) of **Cyano(cyclopropyl)methyl trifluoromethanesulfonate (1s)**

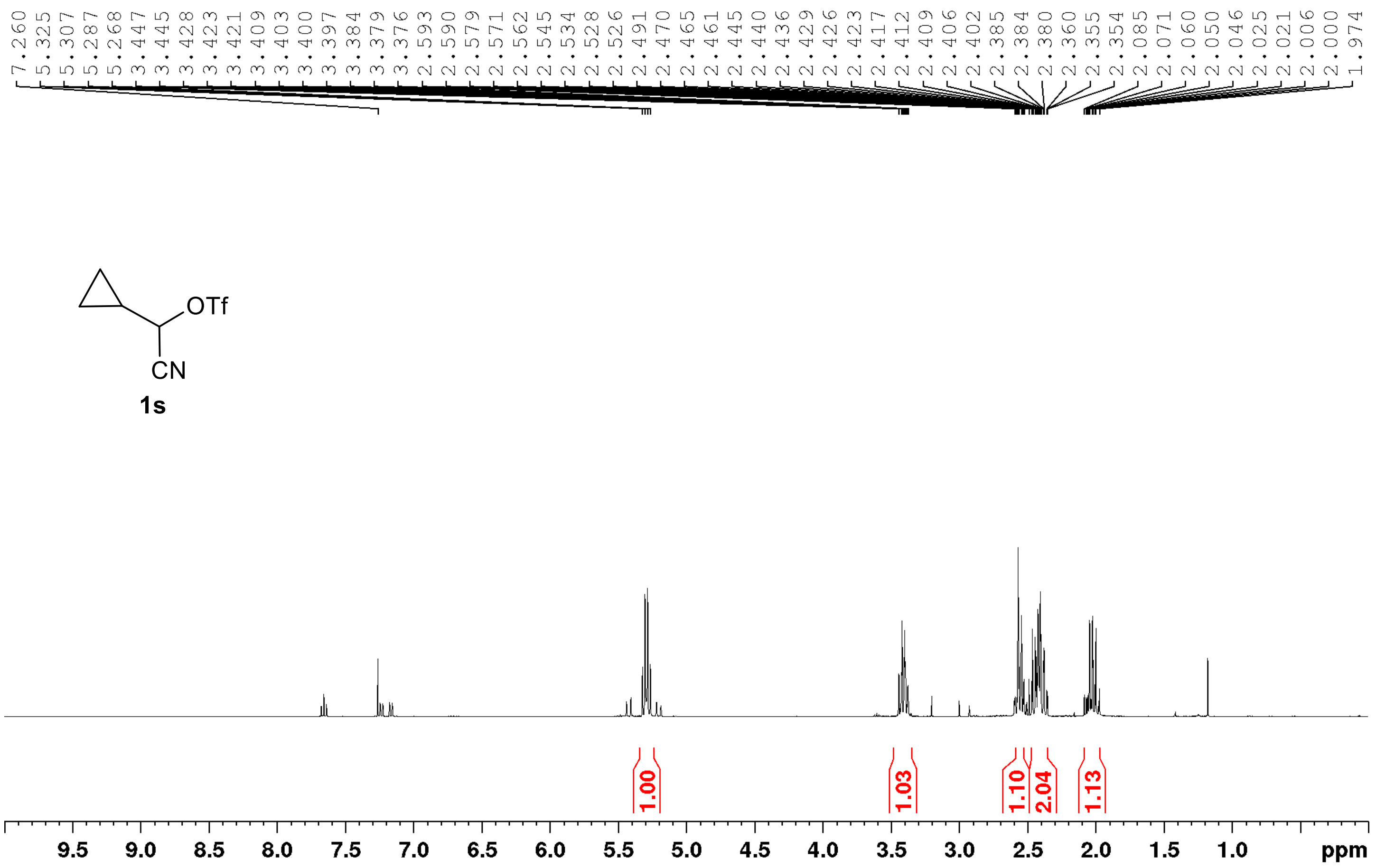

Figure S14. $^{13}C\{^{1}H\}$ **NMR** (125 MHz, $CDCl_3$, 298K) of **Cyano(cyclopropyl)methyl trifluoromethanesulfonate (1s)**

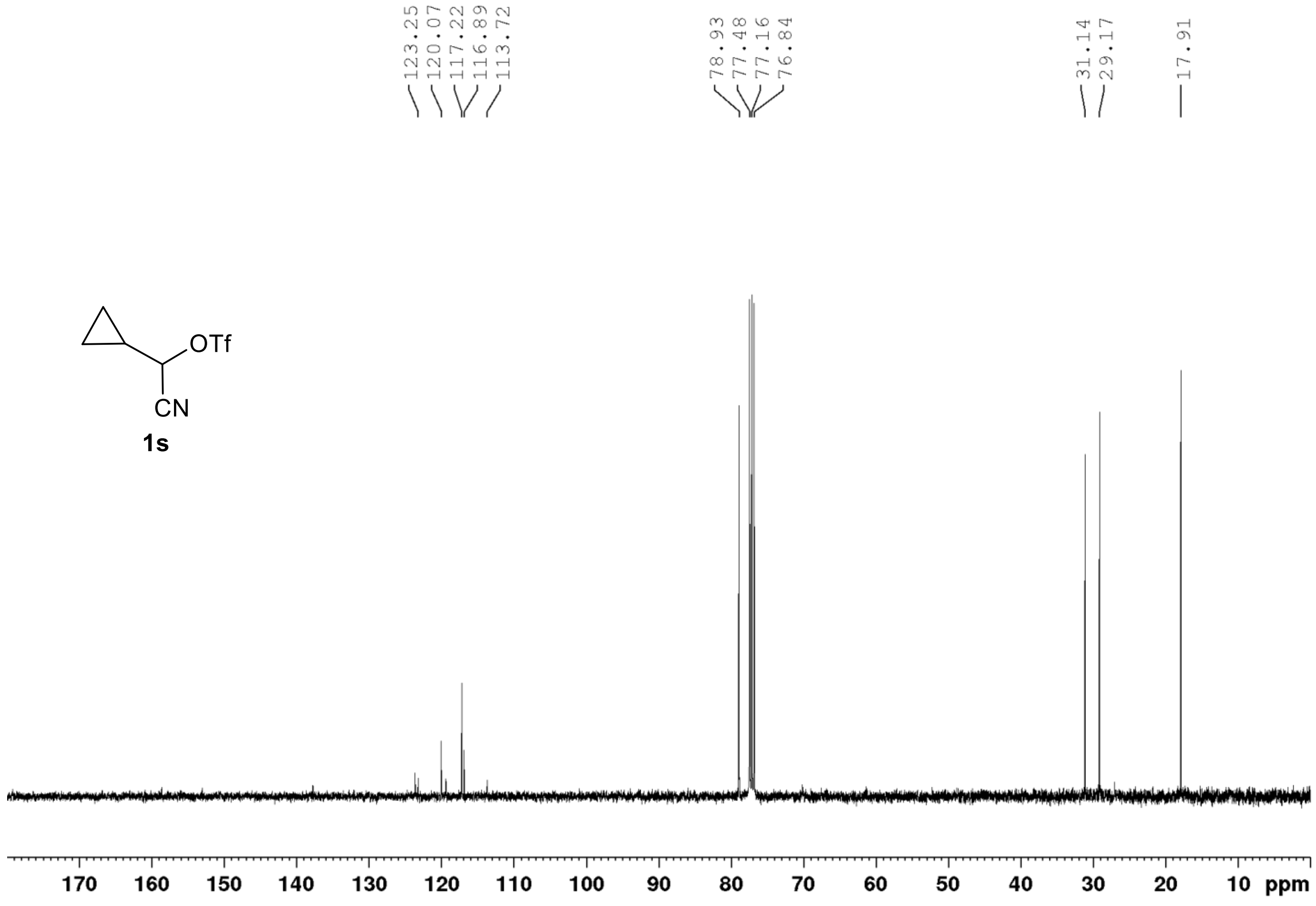

Figure S15. ^{19}F{^{1}H} NMR (471 MHz, $CDCl_3$) of of **Cyano(cyclopropyl)methyl trifluoromethanesulfonate (1s)**

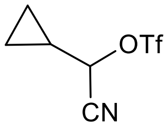

1s

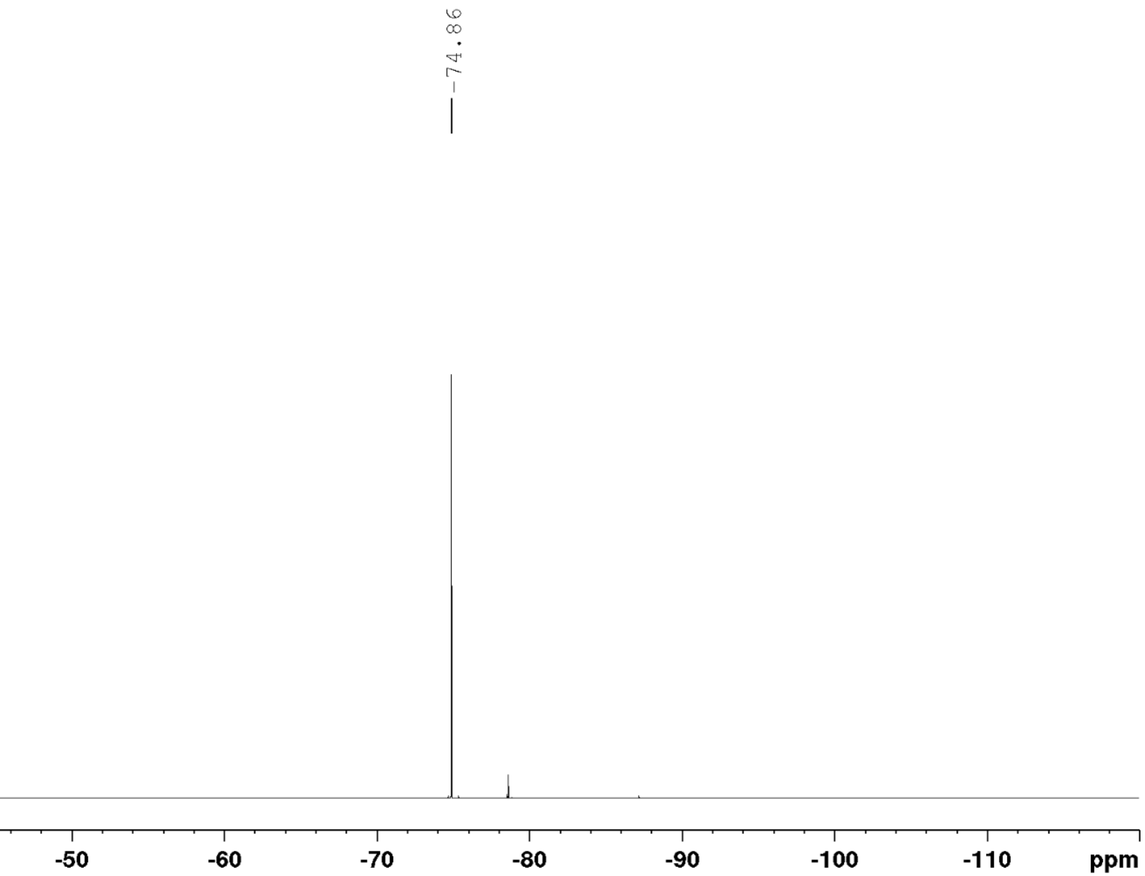

Figure S16. ^{1}**H NMR** (500 MHz, $CDCl_3$, 298K) of **2-(Dimethyl(vinyl)silyl)-4-phenylbutanenitrile (3aa)**

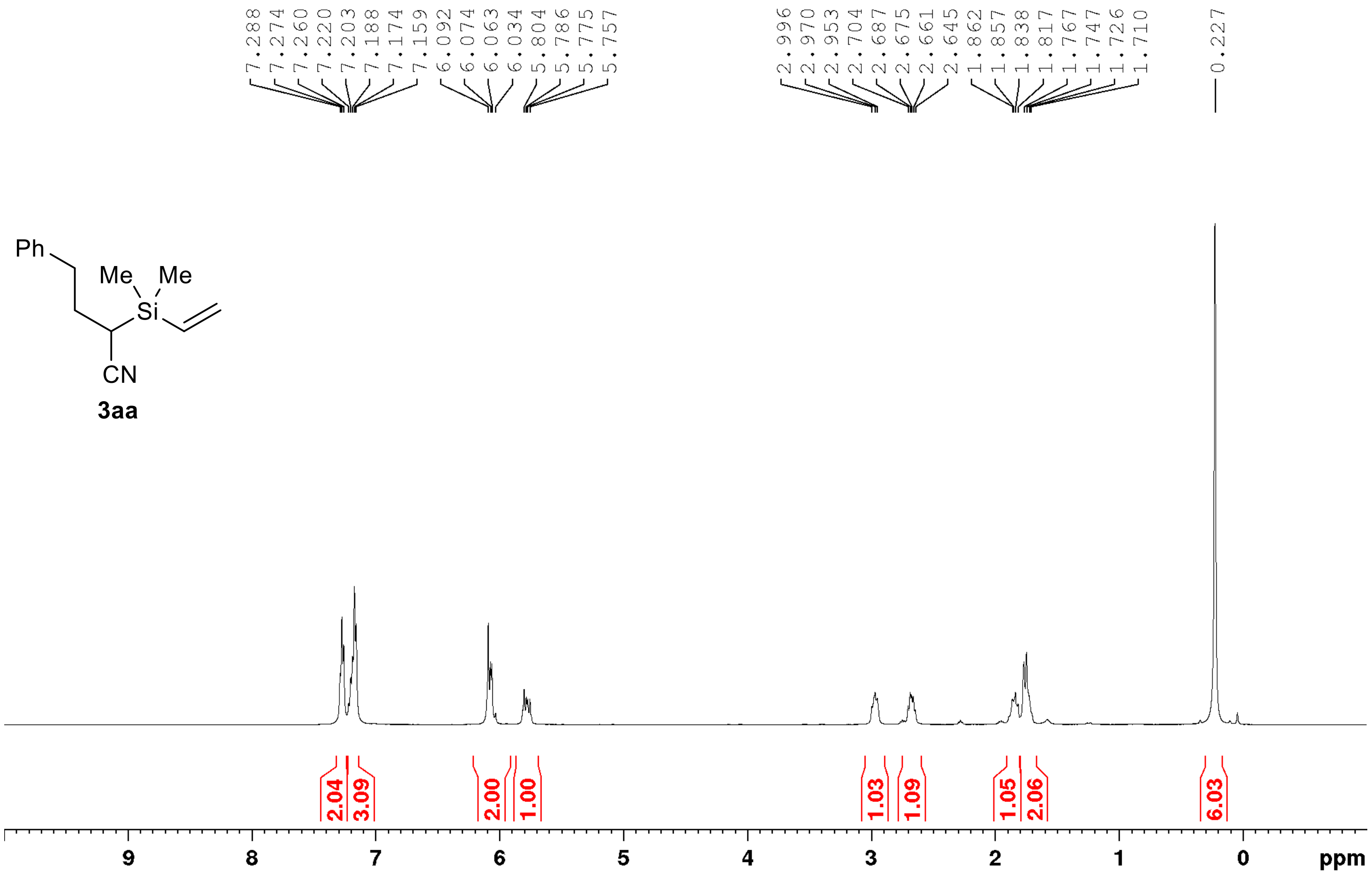

Figure S17. ^{13}**C{**^{1}**H} NMR** (125 MHz, $CDCl_3$, 298K) of **2-(Dimethyl(vinyl)silyl)-4-phenylbutanenitrile (3aa)**

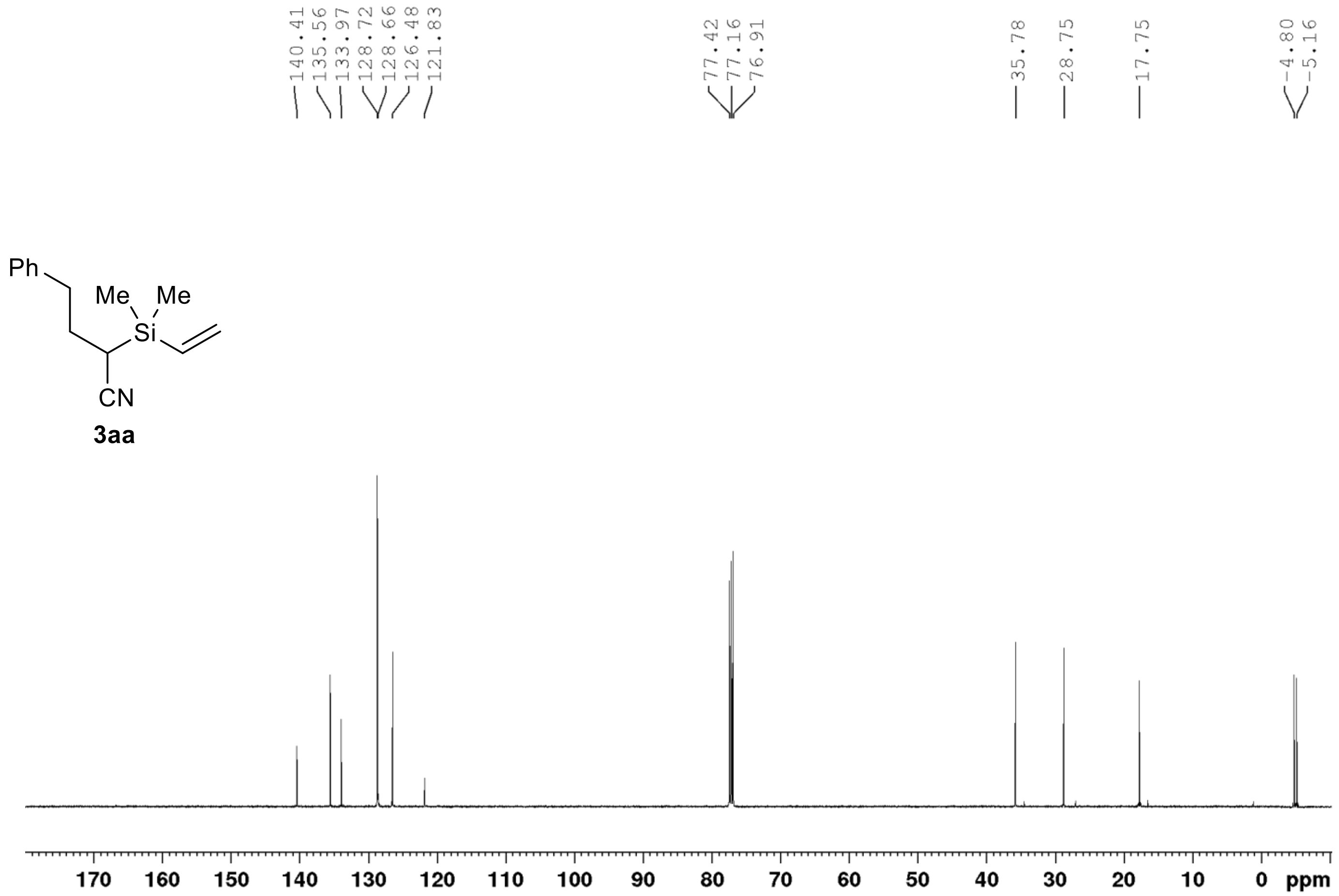

Figure S18. ^{29}**Si{**^{1}**H} DEPT NMR** (99 MHz, $CDCl_3$) of **2-(Dimethyl(vinyl)silyl)-4-phenylbutanenitrile (3aa)**

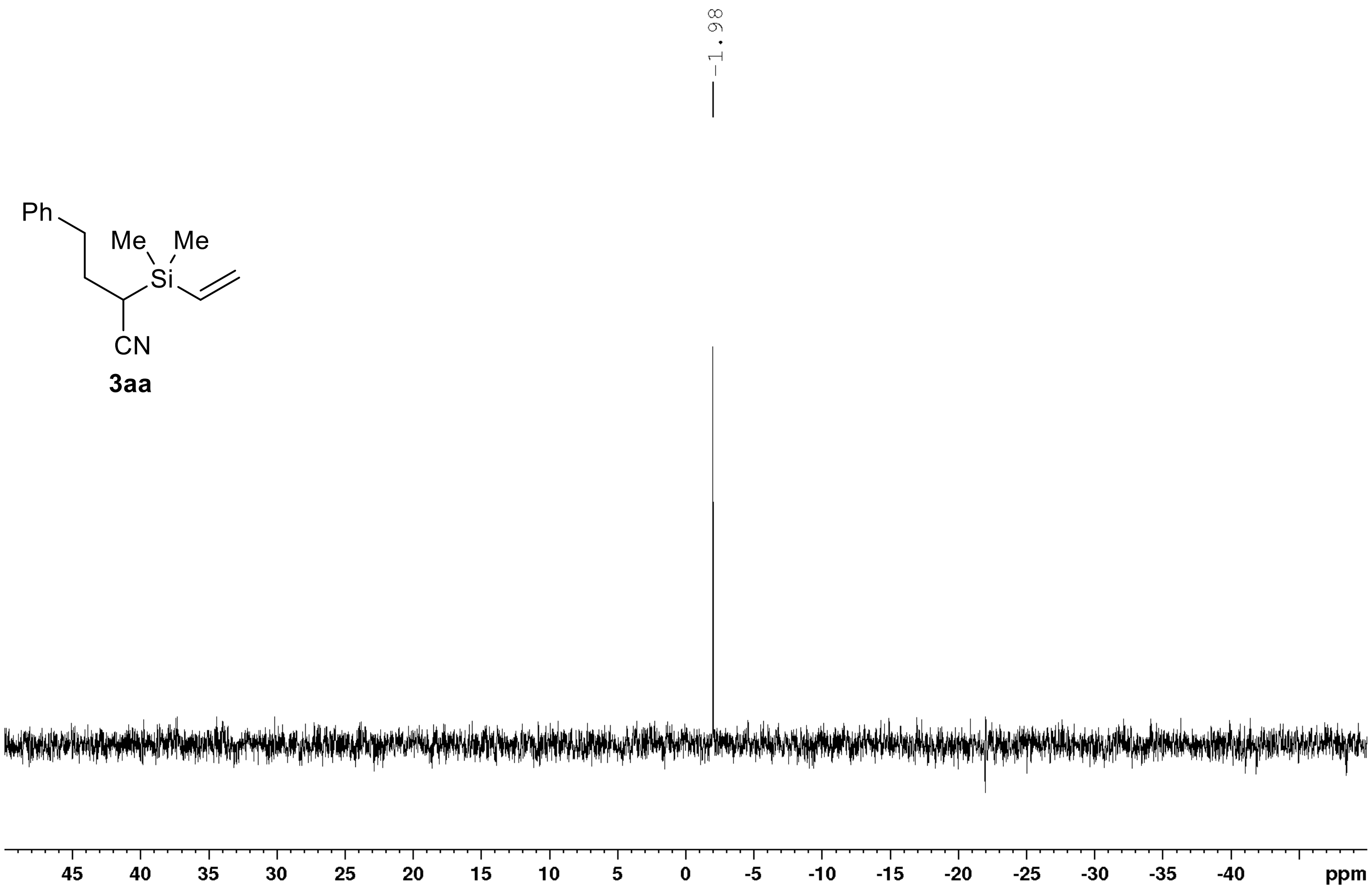

Figure S19. ^{1}H NMR (500 MHz, $CDCl_3$, 298K) of **4-Phenyl-2-(trivinylsilyl)butanenitrile (3ab)**

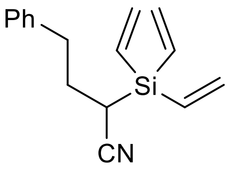

3ab

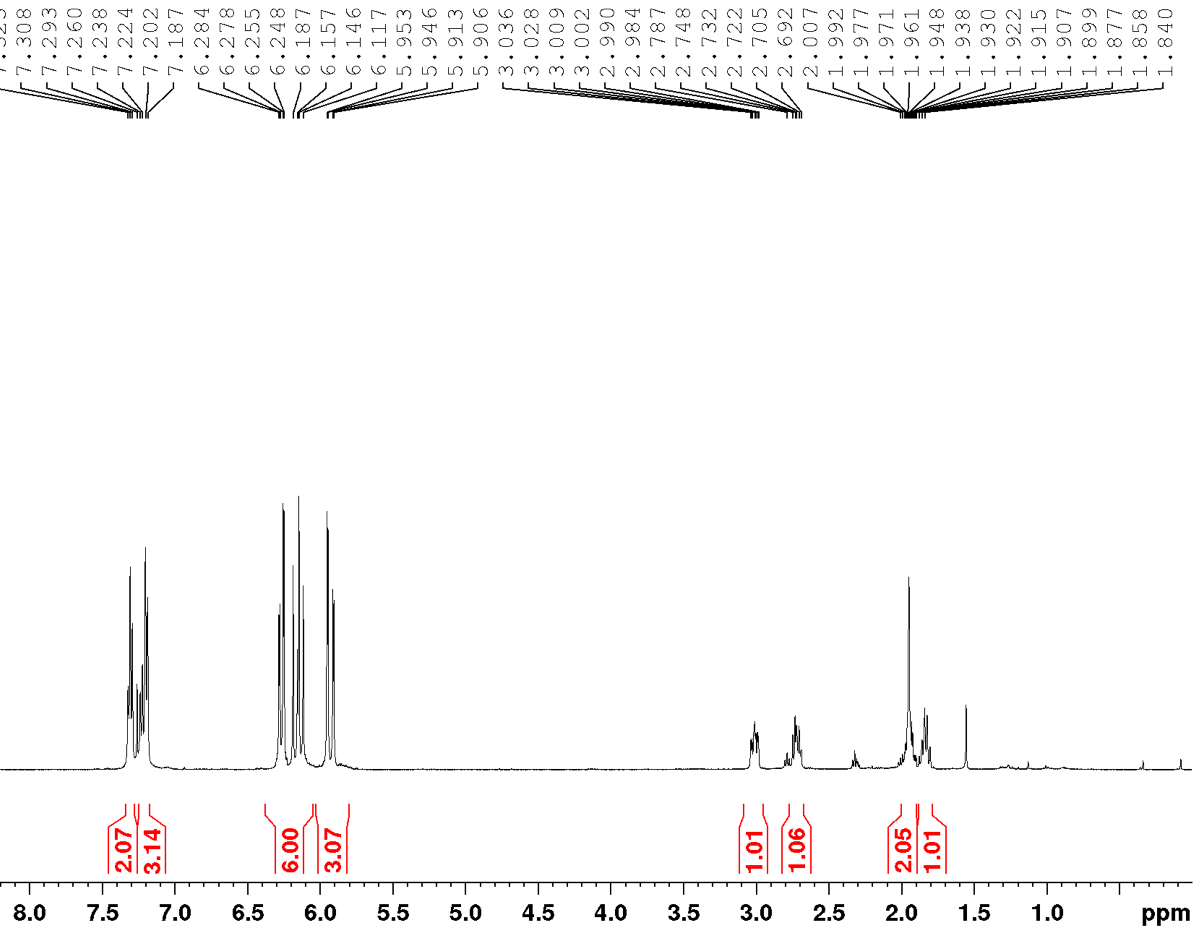

Figure S20. $^{13}C\{^1H\}$ **NMR** (125 MHz, $CDCl_3$, 298K) of **4-Phenyl-2-(trivinylsilyl)butanenitrile (3ab)**

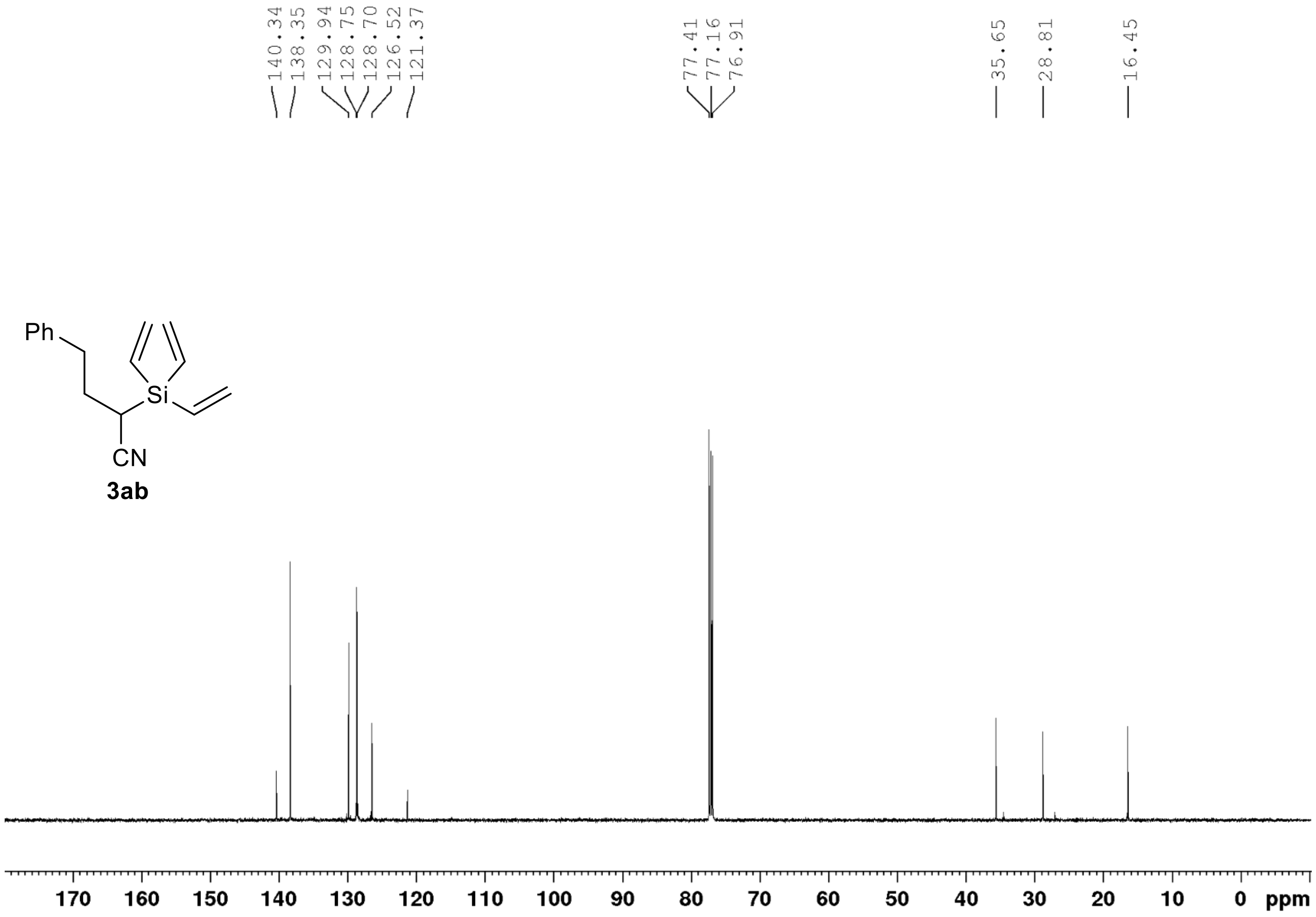

Figure S21. ^{1}**H/**^{29}**Si HMQC NMR** (500/99 MHz, $CDCl_3$, optimized for *J* = 7.0 Hz) of **4-Phenyl-2-(trivinylsilyl)butanenitrile (3ab)**

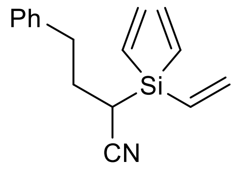

3ab

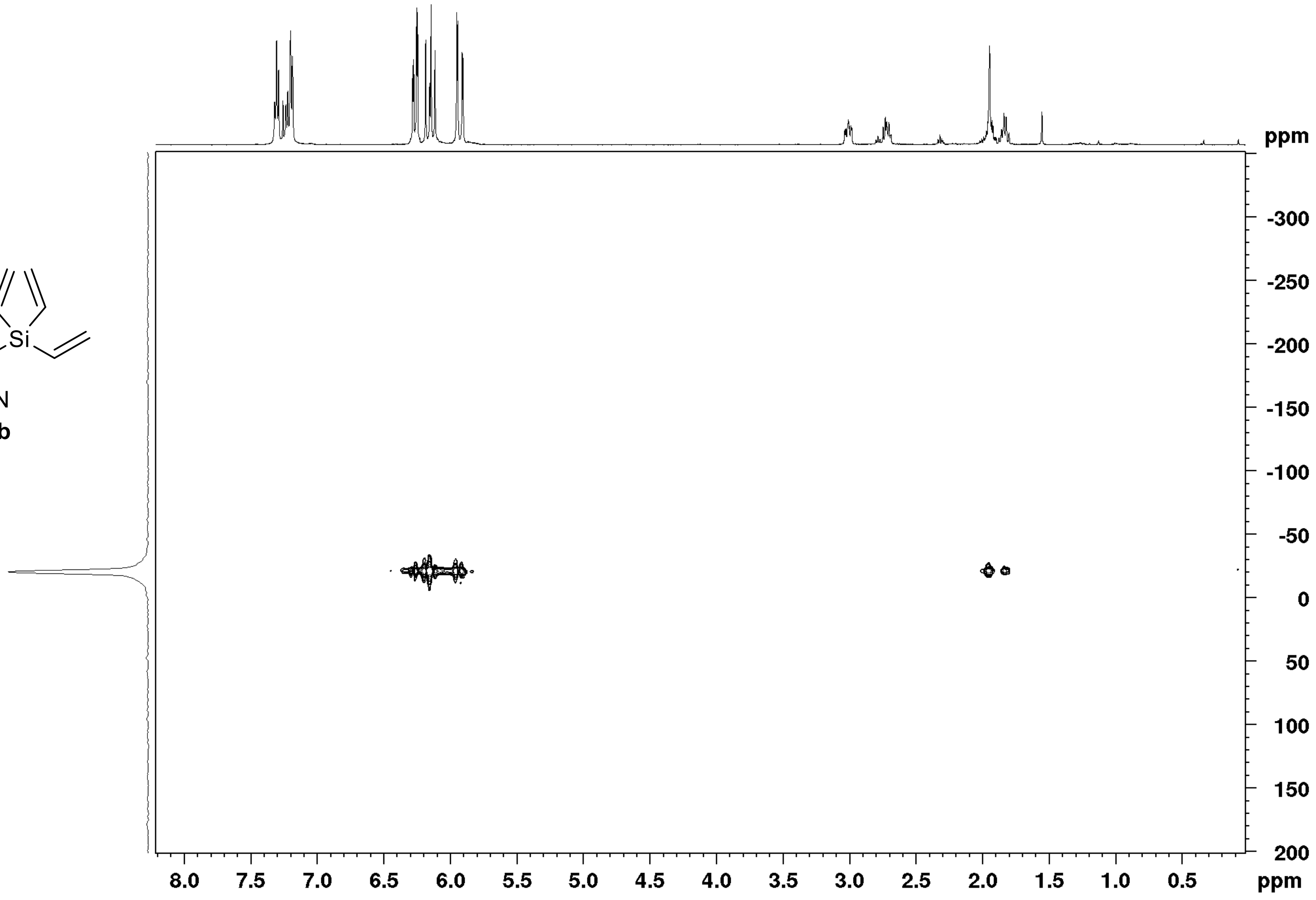

Figure S22. ^{1}H NMR (500 MHz, $CDCl_3$, 298K) of **2-(Ethyldimethylsilyl)-4-phenylbutanenitrile (3ac)**

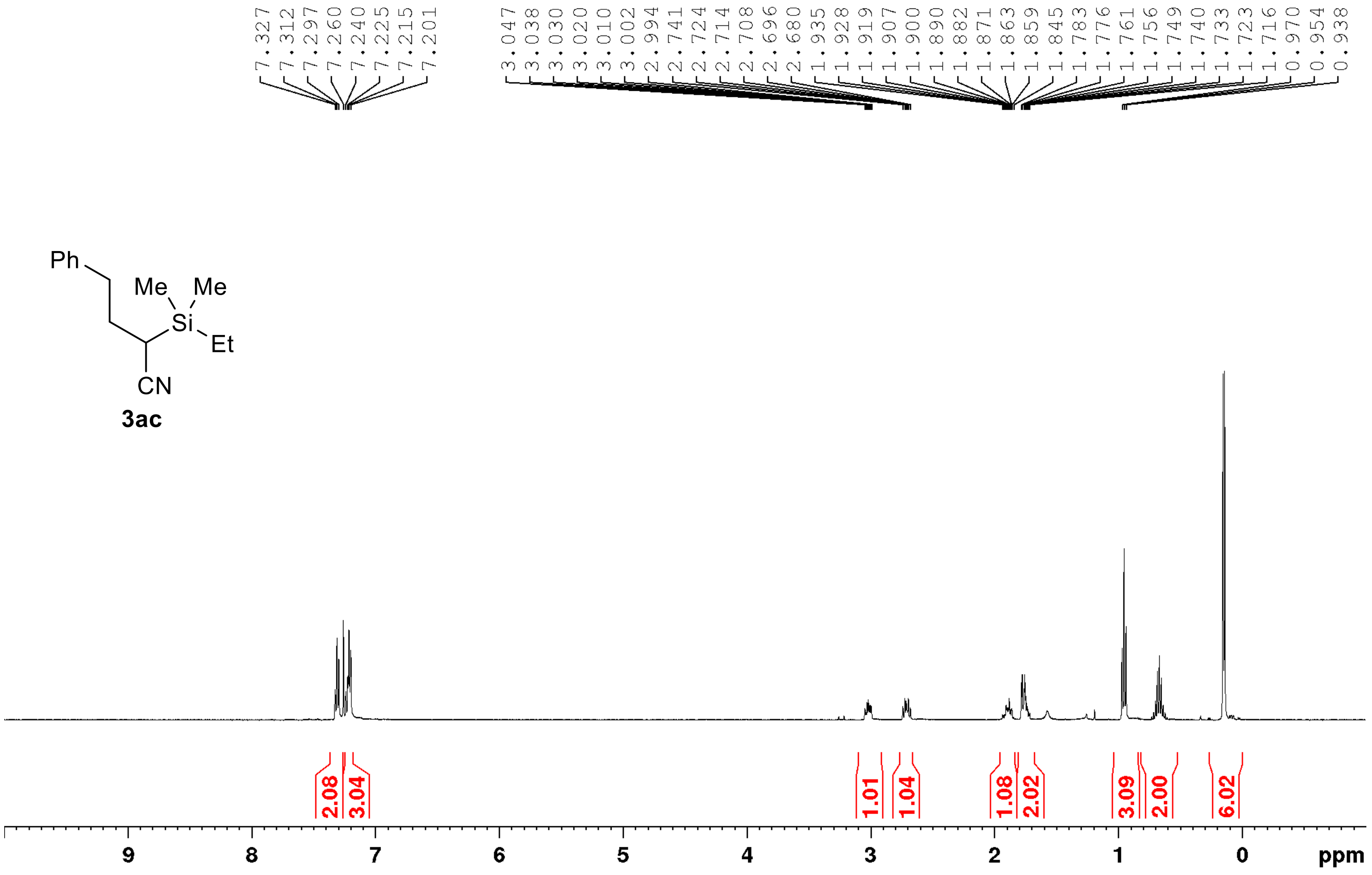

Figure S23. $^{13}C\{^{1}H\}$ NMR (125 MHz, $CDCl_3$, 298K) of **2-(Ethyldimethylsilyl)-4-phenylbutanenitrile (3ac)**

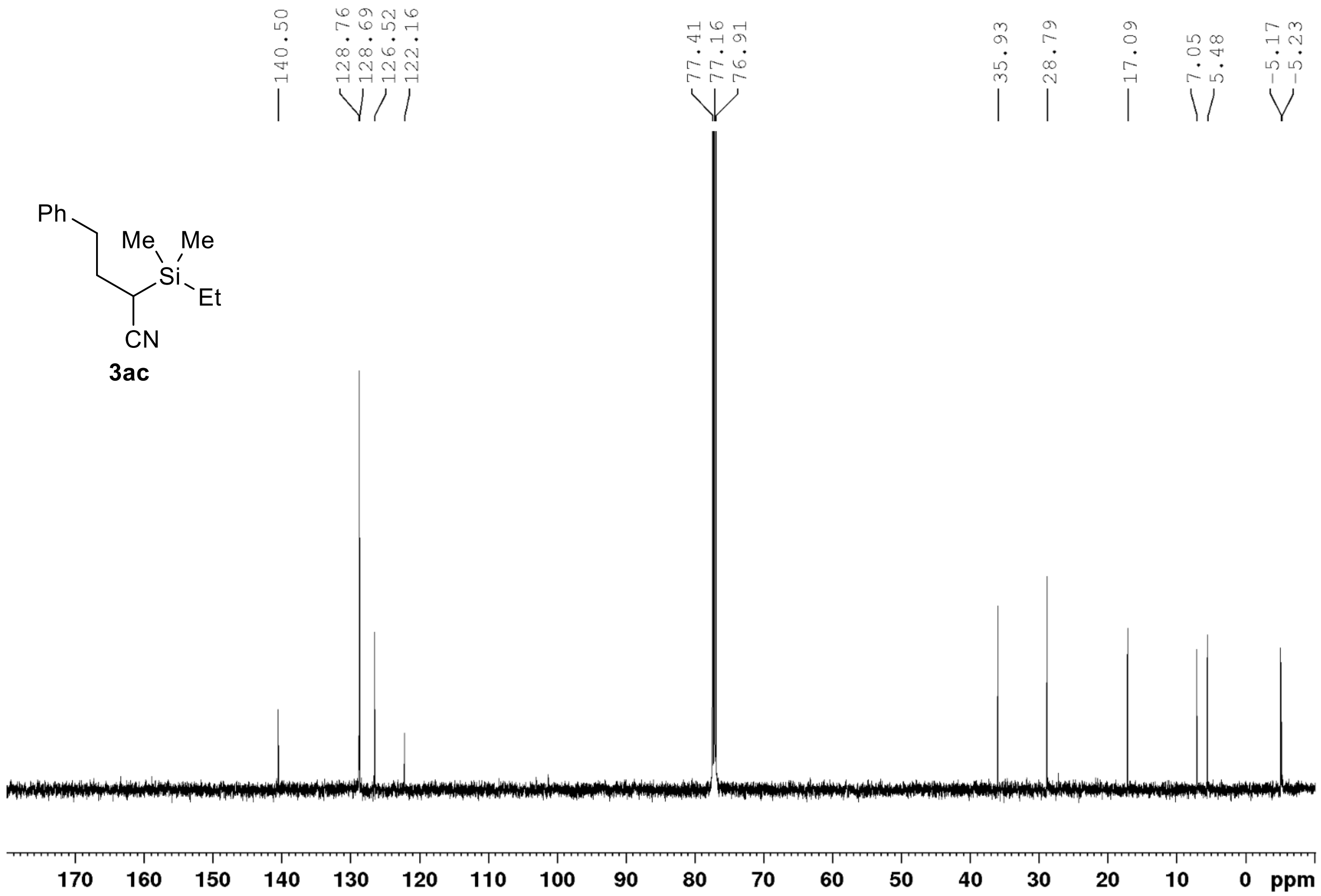

Figure S24. $^{1}H/^{29}Si$ HMQC NMR (500/99 MHz, $CDCl_3$, optimized for J = 7.0 Hz) of **2-(Ethyldimethylsilyl)-4-phenylbutanenitrile (3ac)**

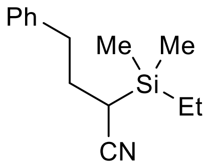

3ac

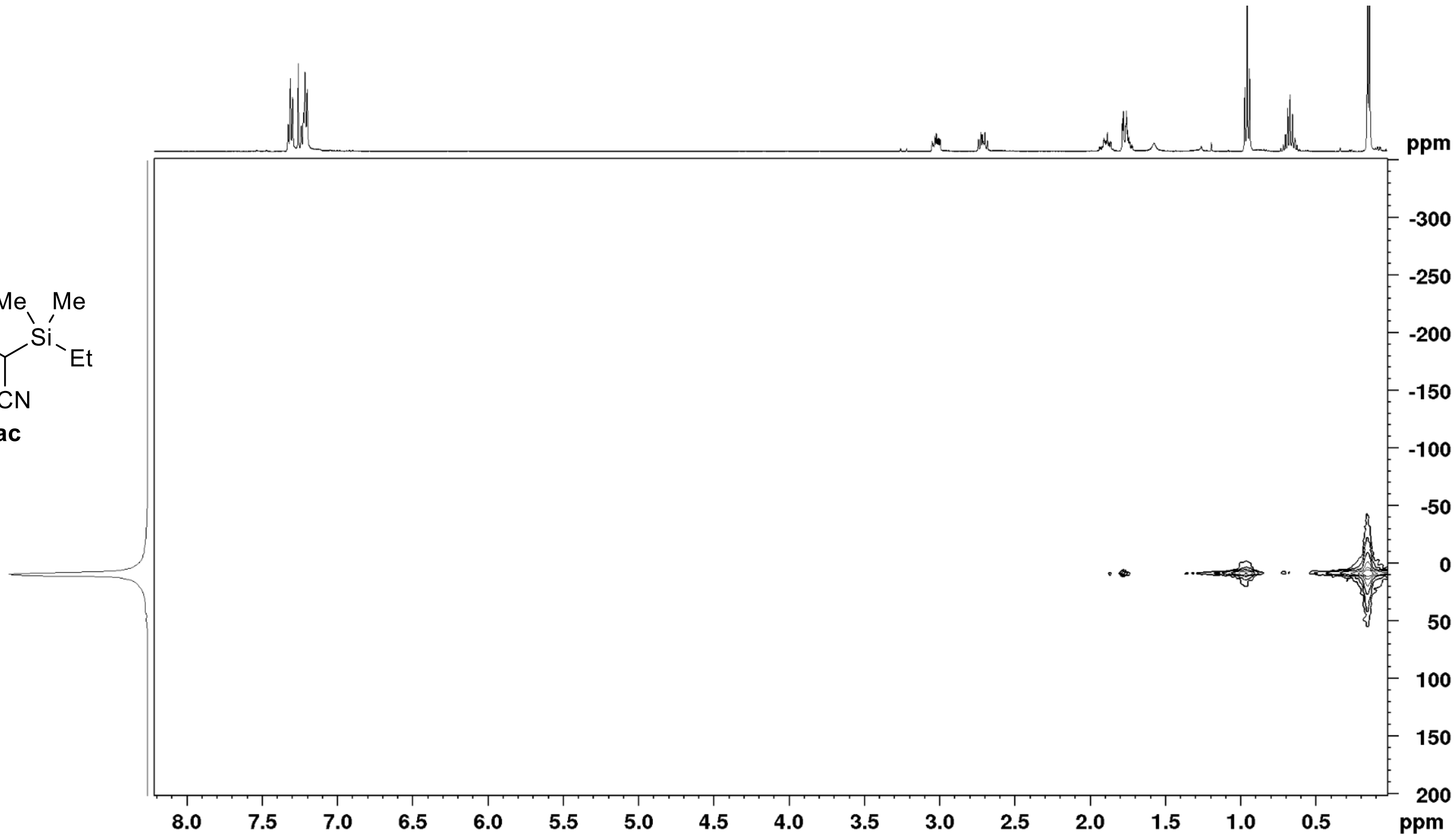

Figure S25. ^{1}H NMR (500 MHz, $CDCl_3$, 298K) of **4-Phenyl-2-(trimethylsilyl)butanenitrile (3ad)**

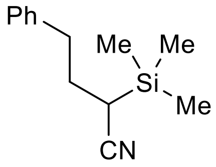

3ad

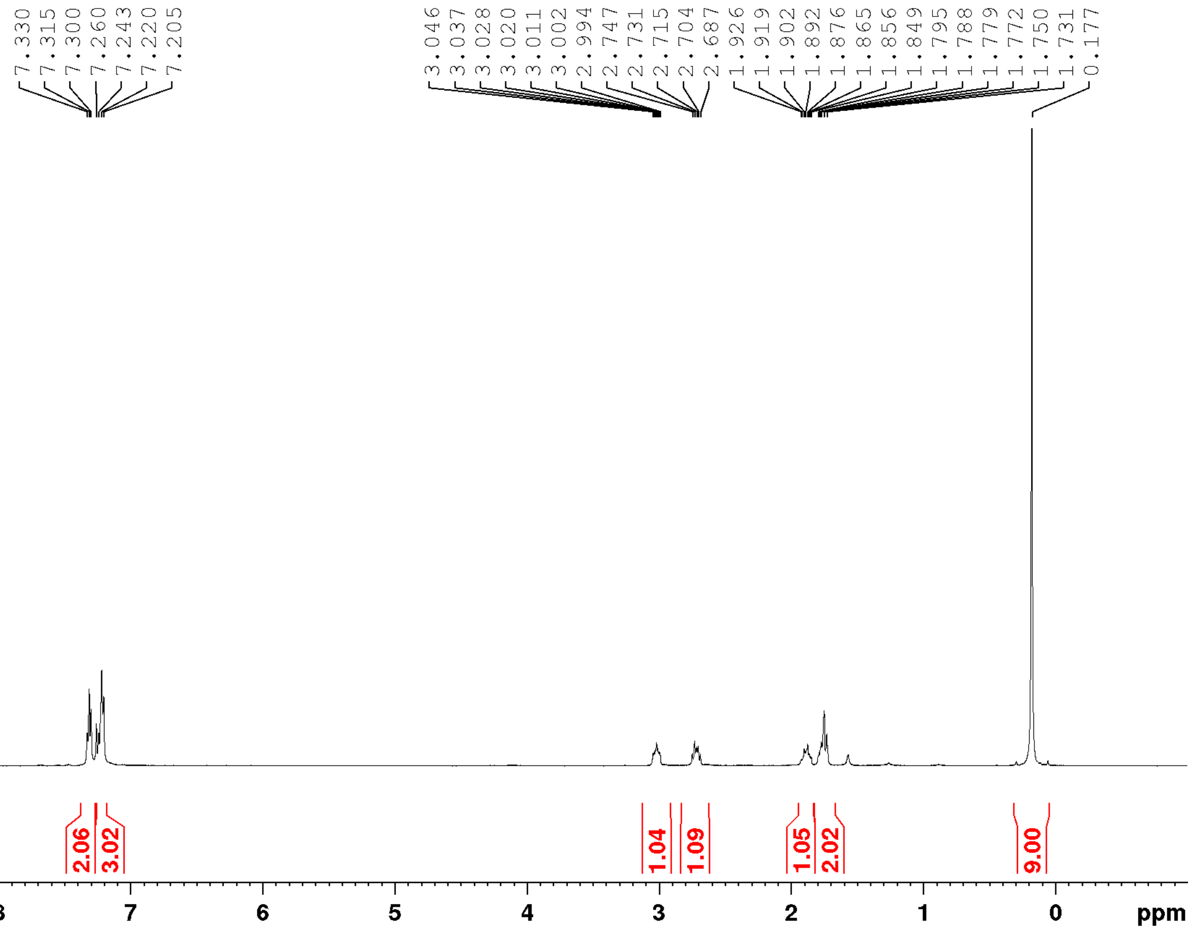

Figure S26. $^{13}C\{^1H\}$ **NMR** (125 MHz, $CDCl_3$, 298K) of **4-Phenyl-2-(trimethylsilyl)butanenitrile (3ad)**

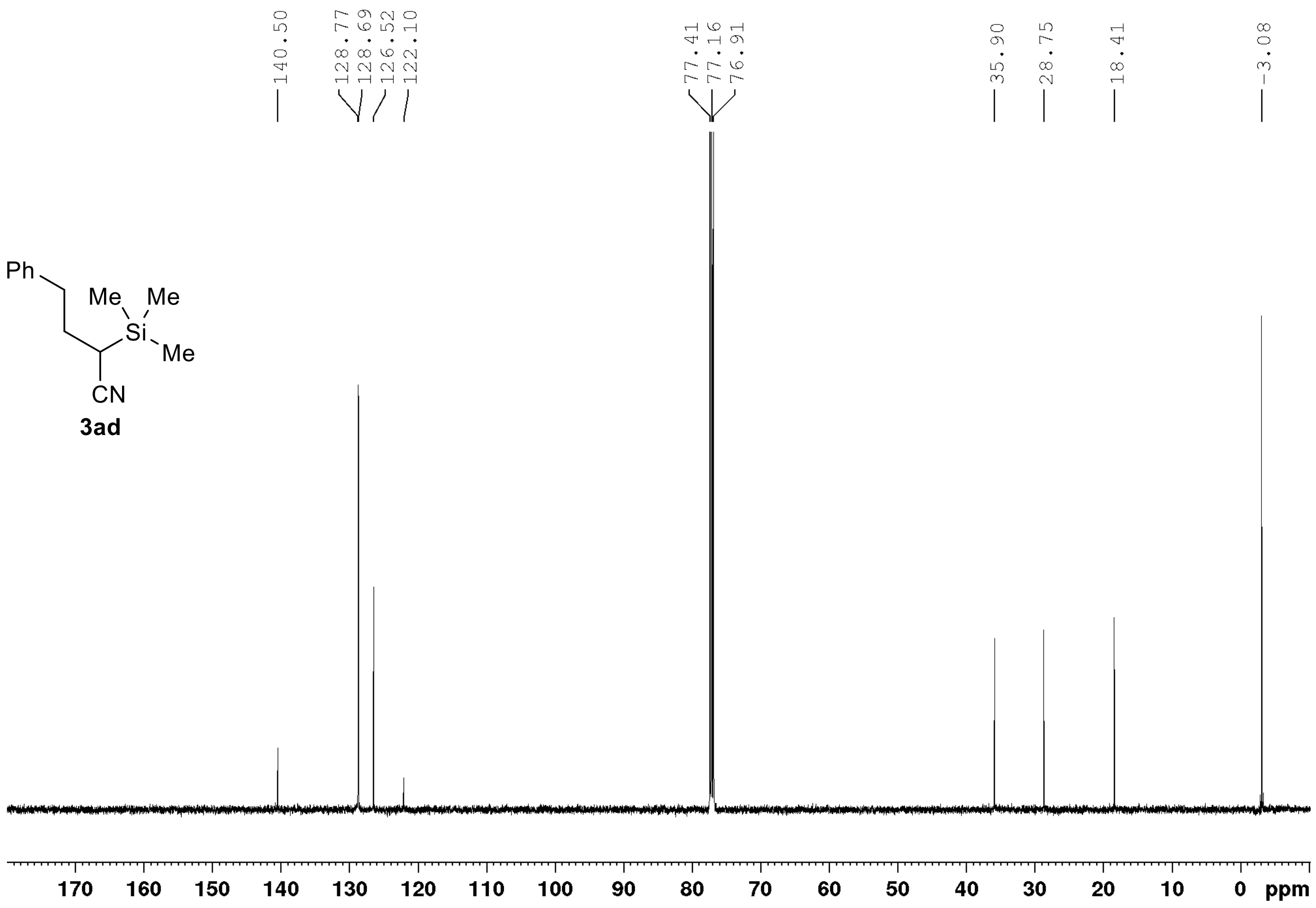

Figure S27. **$^{1}H/^{29}Si$ HMQC NMR** (500/99 MHz, $CDCl_3$, optimized for J = 7.0 Hz) of **4-Phenyl-2-(trimethylsilyl)butanenitrile (3ad)**

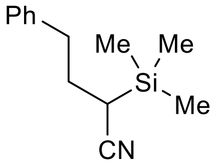

3ad

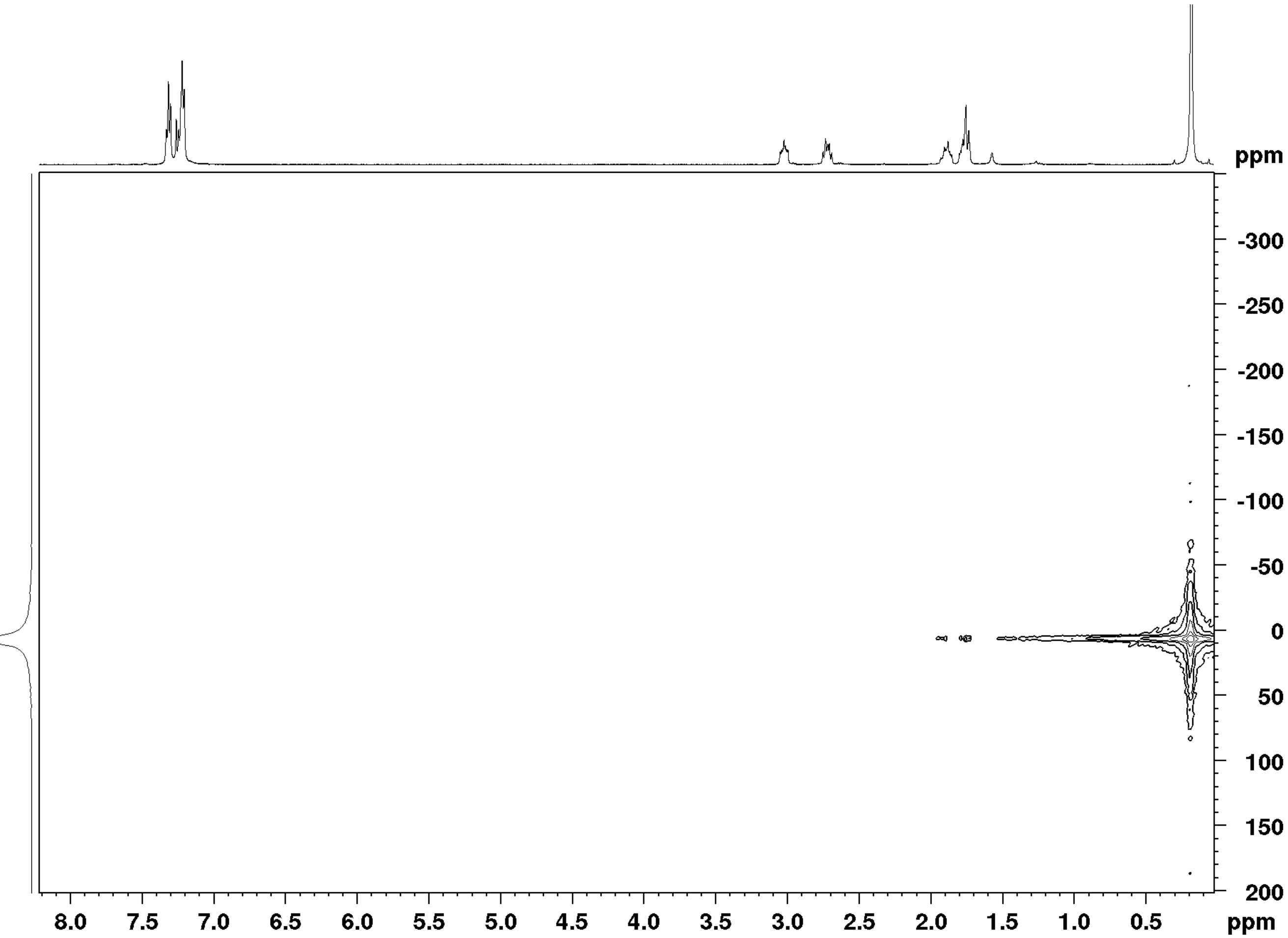

Figure S28. **^{1}H NMR** (500 MHz, $CDCl_3$, 298K) of **4-(4-Chlorophenyl)-2-(dimethyl(vinyl)silyl)butanenitrile (3ba)**
(with small amounts of unknown impurities in the aliphatic region)

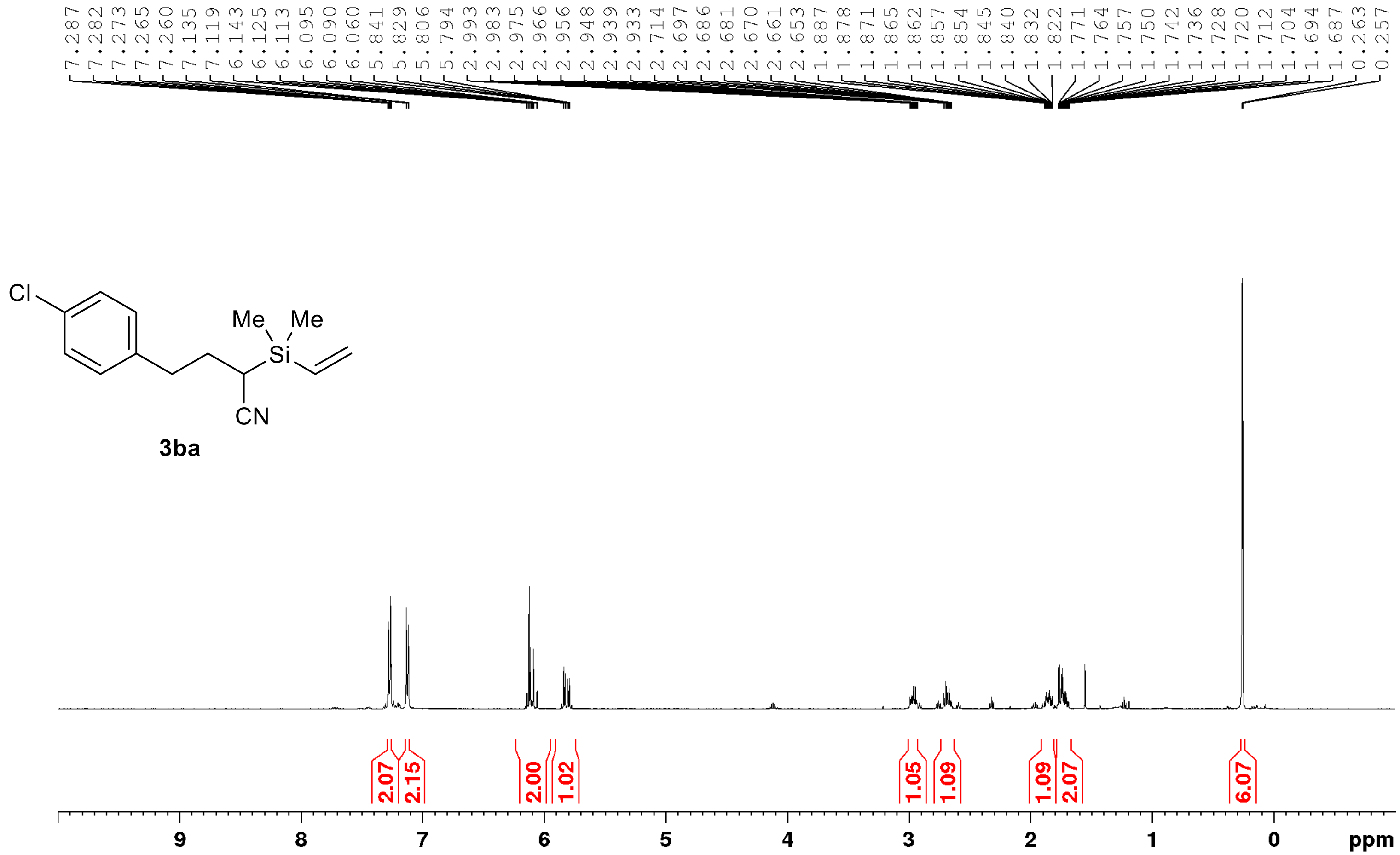

Figure S29. $^{13}C\{^{1}H\}$ **NMR** (125 MHz, $CDCl_3$, 298K) of **4-(4-Chlorophenyl)-2-(dimethyl(vinyl)silyl)butanenitrile (3ba)** (with small amounts of unknown impurities in the aliphatic region)

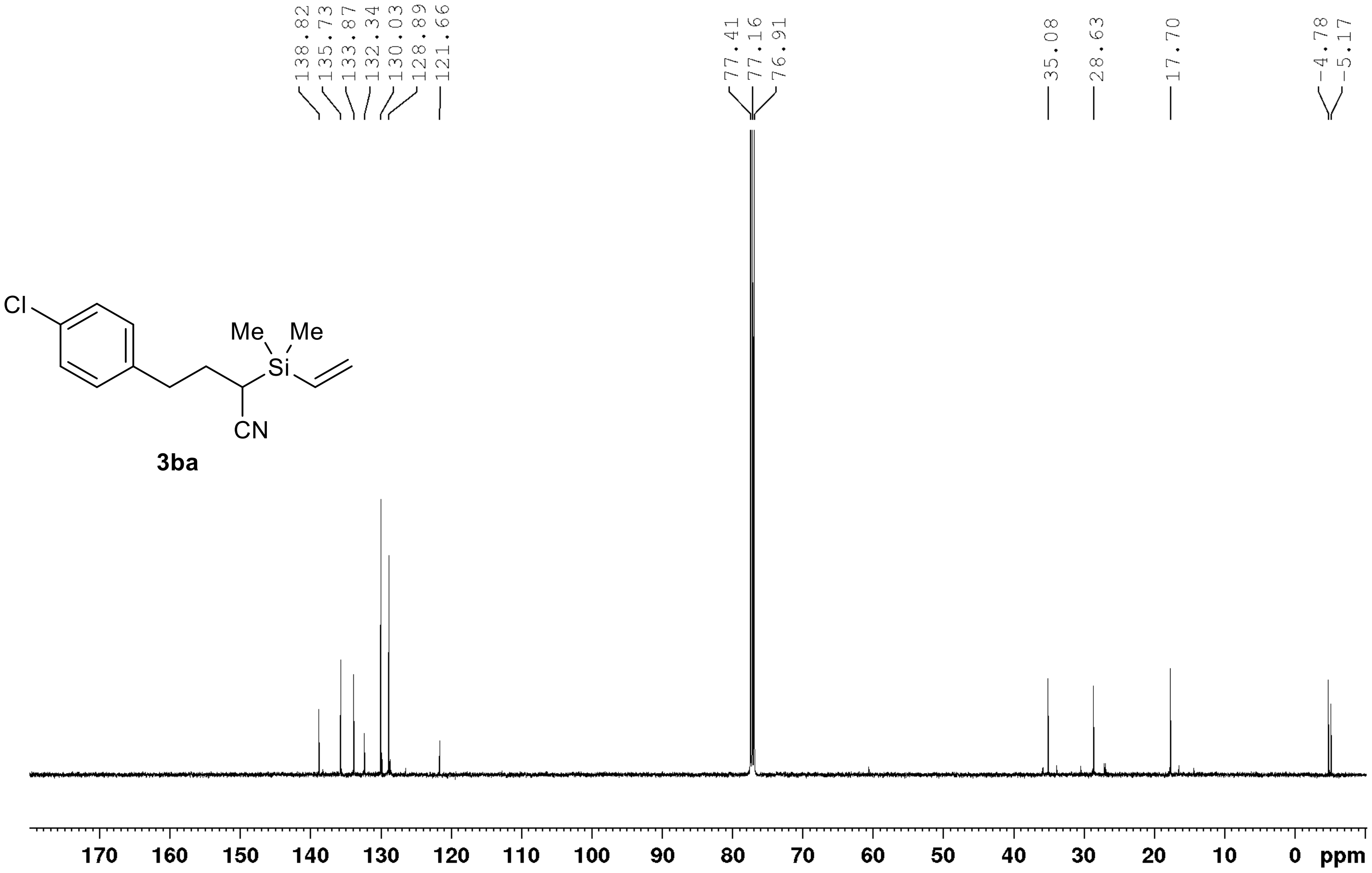

Figure S30. $^{1}H/^{29}Si$ HMQC NMR (500/99 MHz, $CDCl_3$, optimized for J = 7.0 Hz) of **4-(4-Chlorophenyl)-2-(dimethyl(vinyl)silyl)butanenitrile (3ba)**

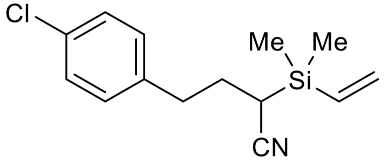

3ba

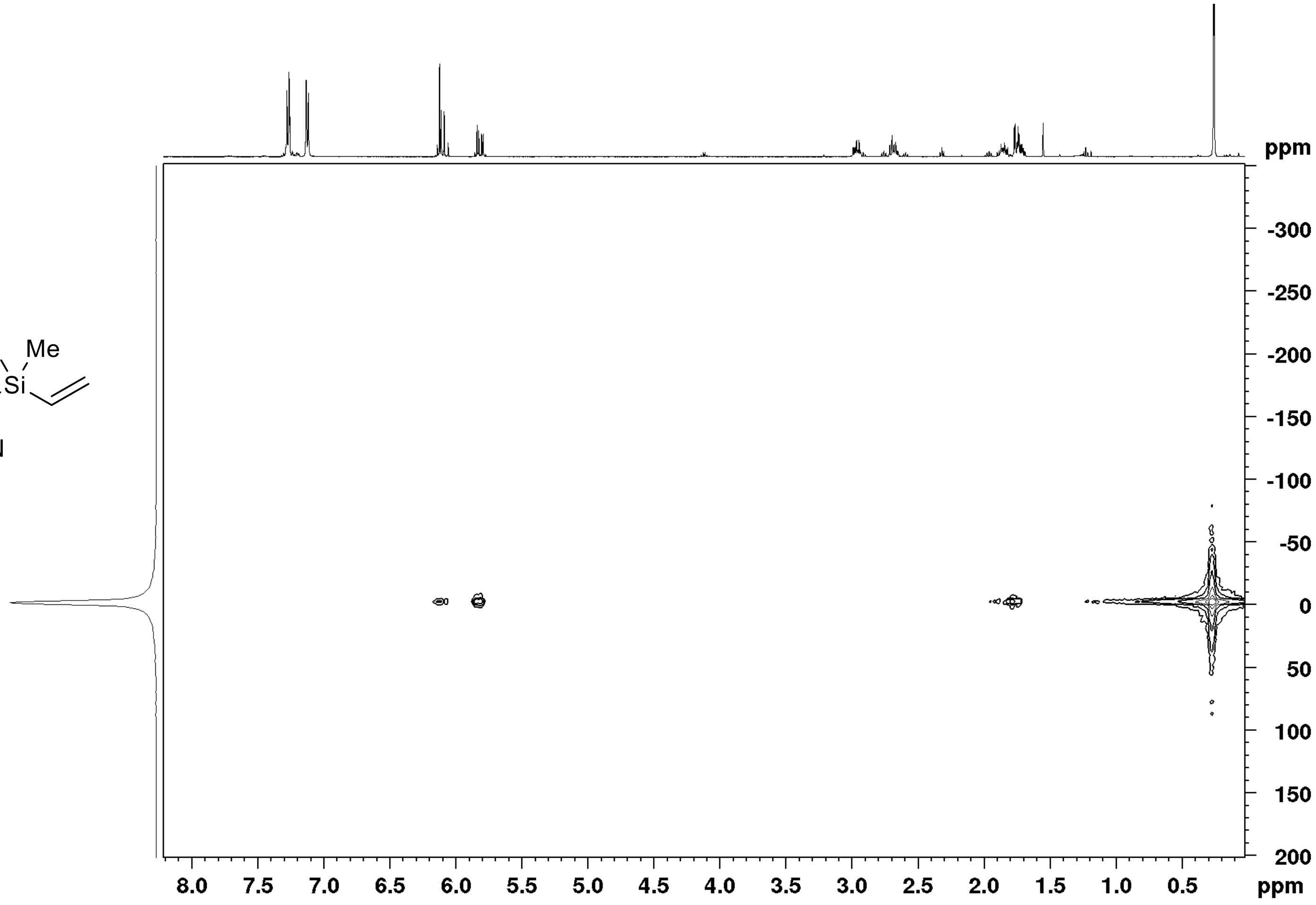

Figure S31. **^{1}H NMR** (500 MHz, $CDCl_3$, 298K) of **4-(4-Bromophenyl)-2-(dimethyl(vinyl)silyl)butanenitrile (3ca)**
(with small amounts of unknown impurities in the aliphatic region)

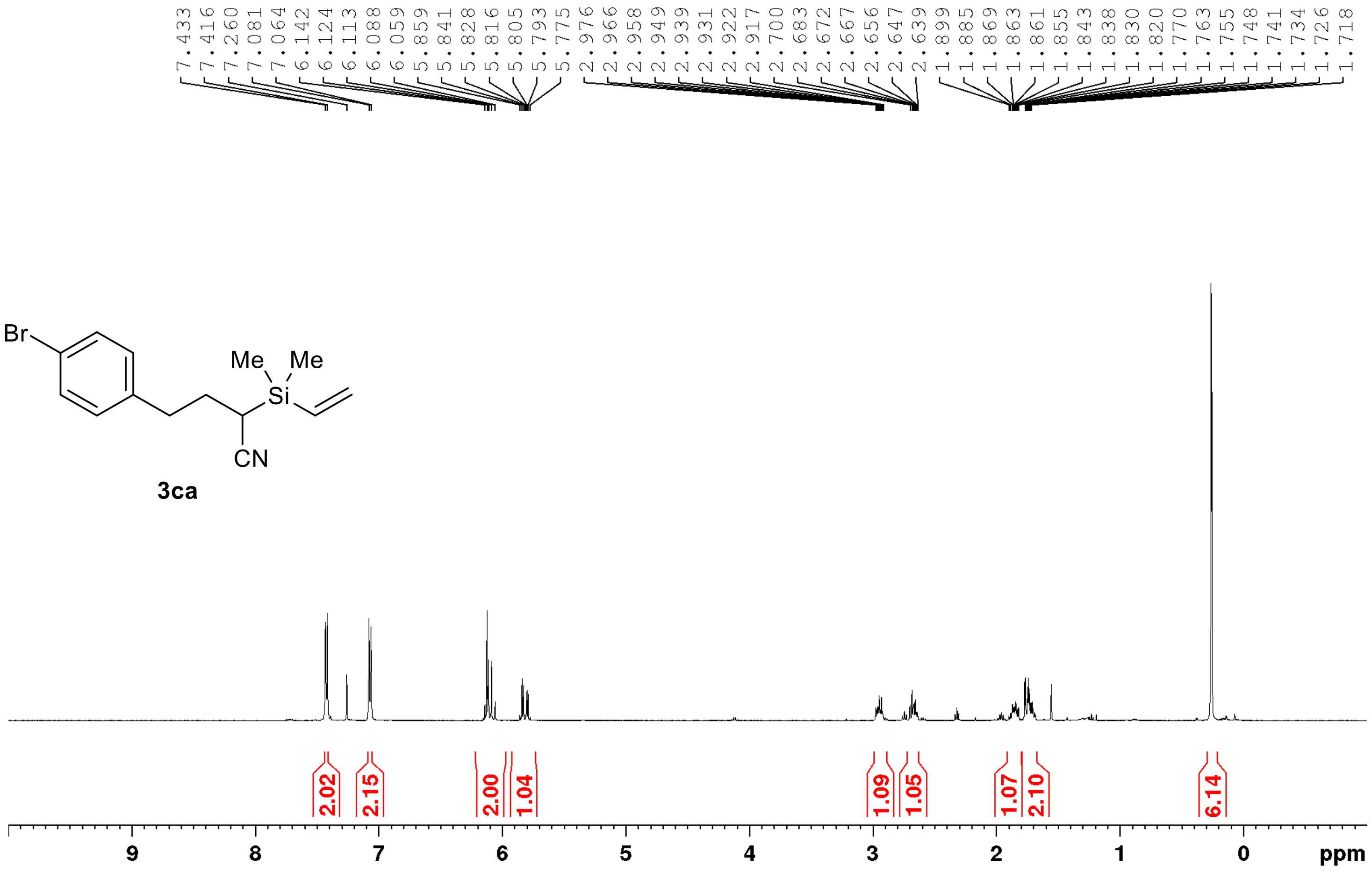

Figure S32. ^{13}**C{**^{1}**H} NMR** (125 MHz, $CDCl_3$, 298K) of **4-(4-Bromophenyl)-2-(dimethyl(vinyl)silyl)butanenitrile (3ca)** (with small amounts of unknown impurities in the aliphatic region)

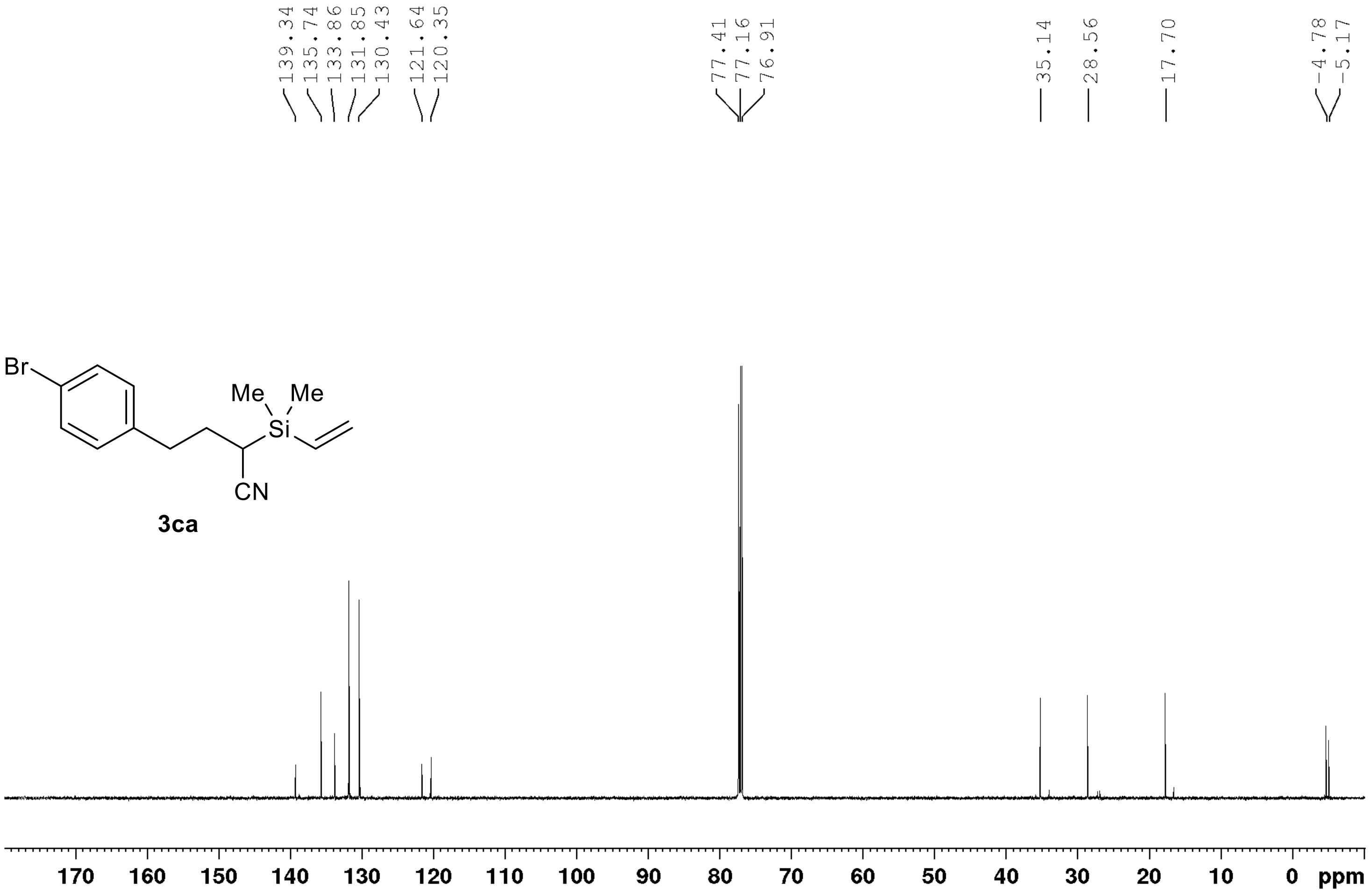

Figure S33. ^{1}H/^{29}Si HMQC NMR (500/99 MHz, $CDCl_3$, optimized for *J* = 7.0 Hz) of **4-(4-Bromophenyl)-2-(dimethyl(vinyl)silyl)butanenitrile (3ca)**

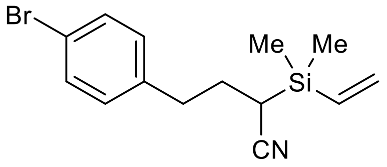

3ca

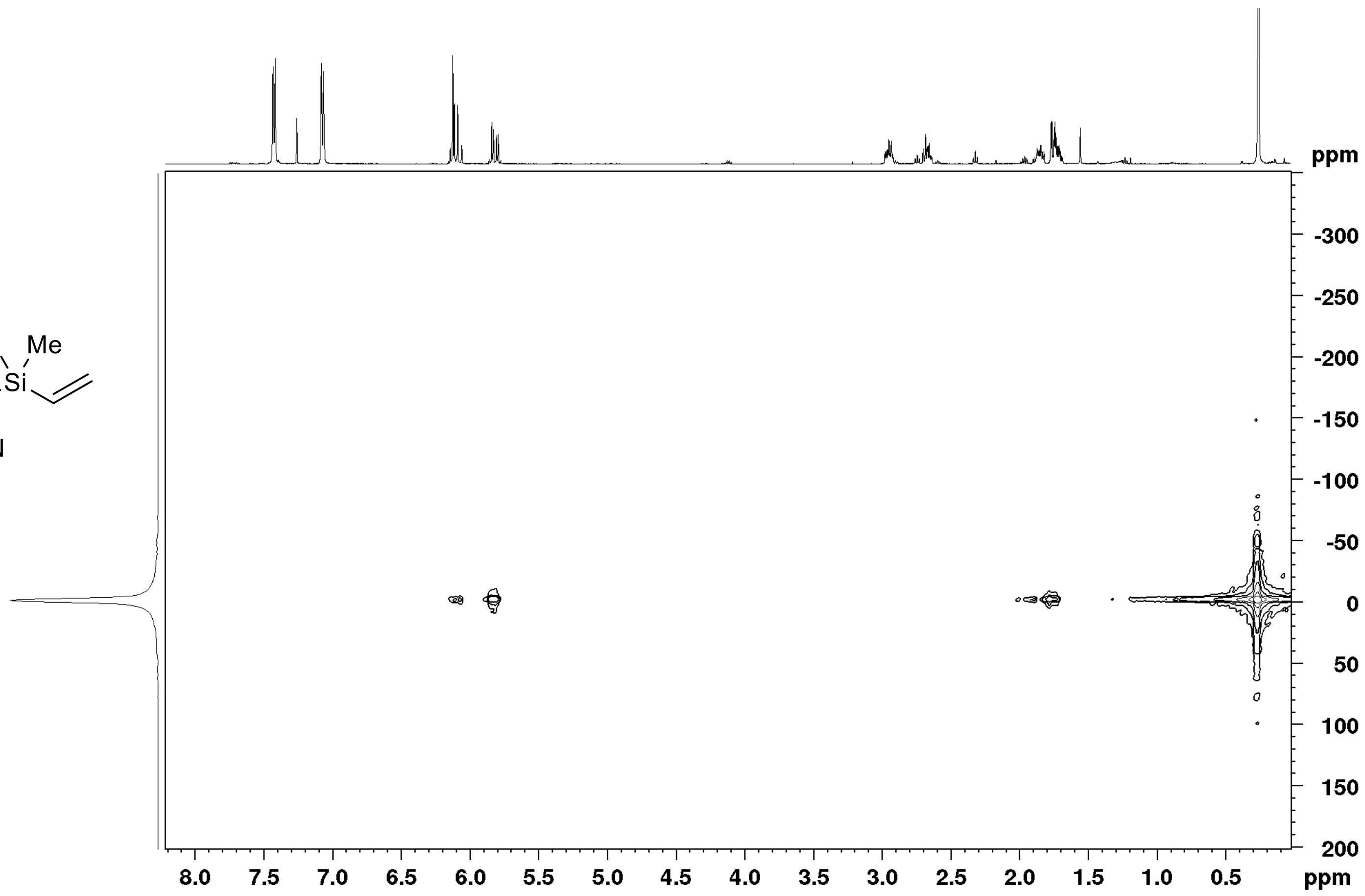

Figure S34. **^{1}H NMR** (500 MHz, $CDCl_3$, 298K) of **2-(Dimethyl(vinyl)silyl)-4-(5-methylfuran-2-yl)butanenitrile (3da)** (with small amounts of unknown impurities in the aliphatic region)

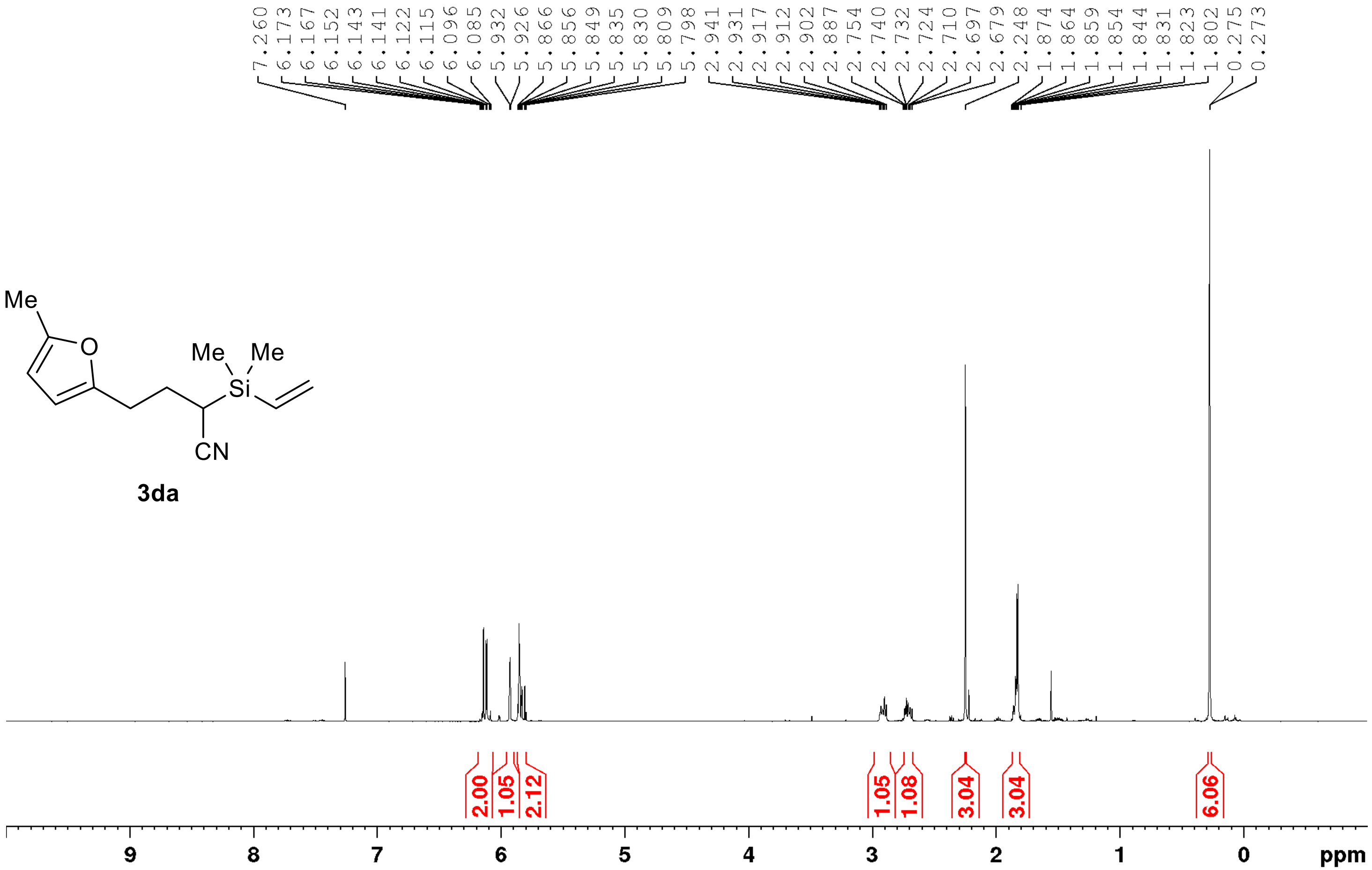

Figure S35. ^{13}**C{**^{1}**H} NMR** (125 MHz, $CDCl_3$, 298K) of **2-(Dimethyl(vinyl)silyl)-4-(5-methylfuran-2-yl)butanenitrile (3da)** (with small amounts of unknown impurities in the aliphatic region)

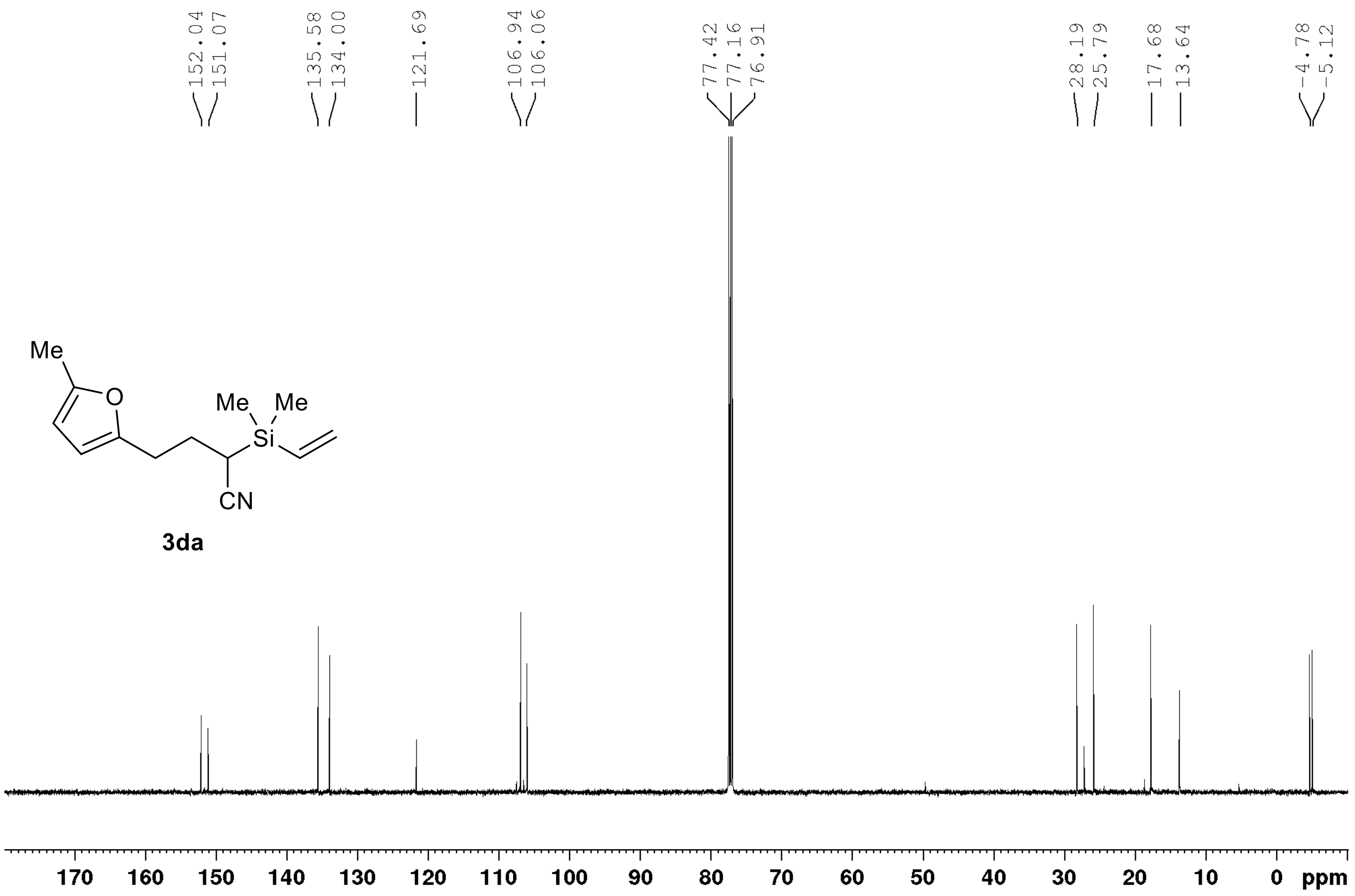

Figure S36. ^{29}Si{^{1}H} DEPT NMR (99 MHz, $CDCl_3$) of **2-(Dimethyl(vinyl)silyl)-4-(5-methylfuran-2-yl)butanenitrile (3da)**

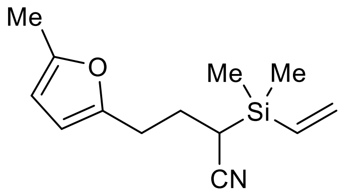

3da

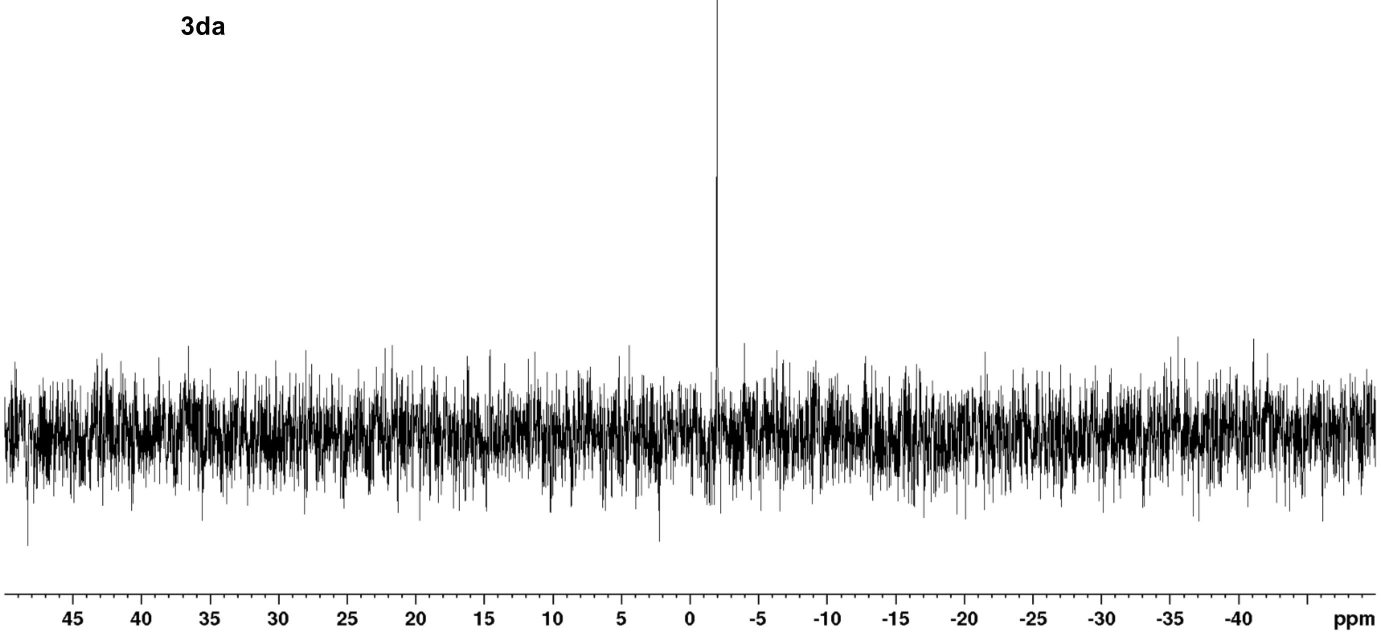

Figure S37. ^{1}H NMR (500 MHz, $CDCl_3$, 298K) of **2-(Dimethyl(vinyl)silyl)-6-phenylhexanenitrile (3ea)**

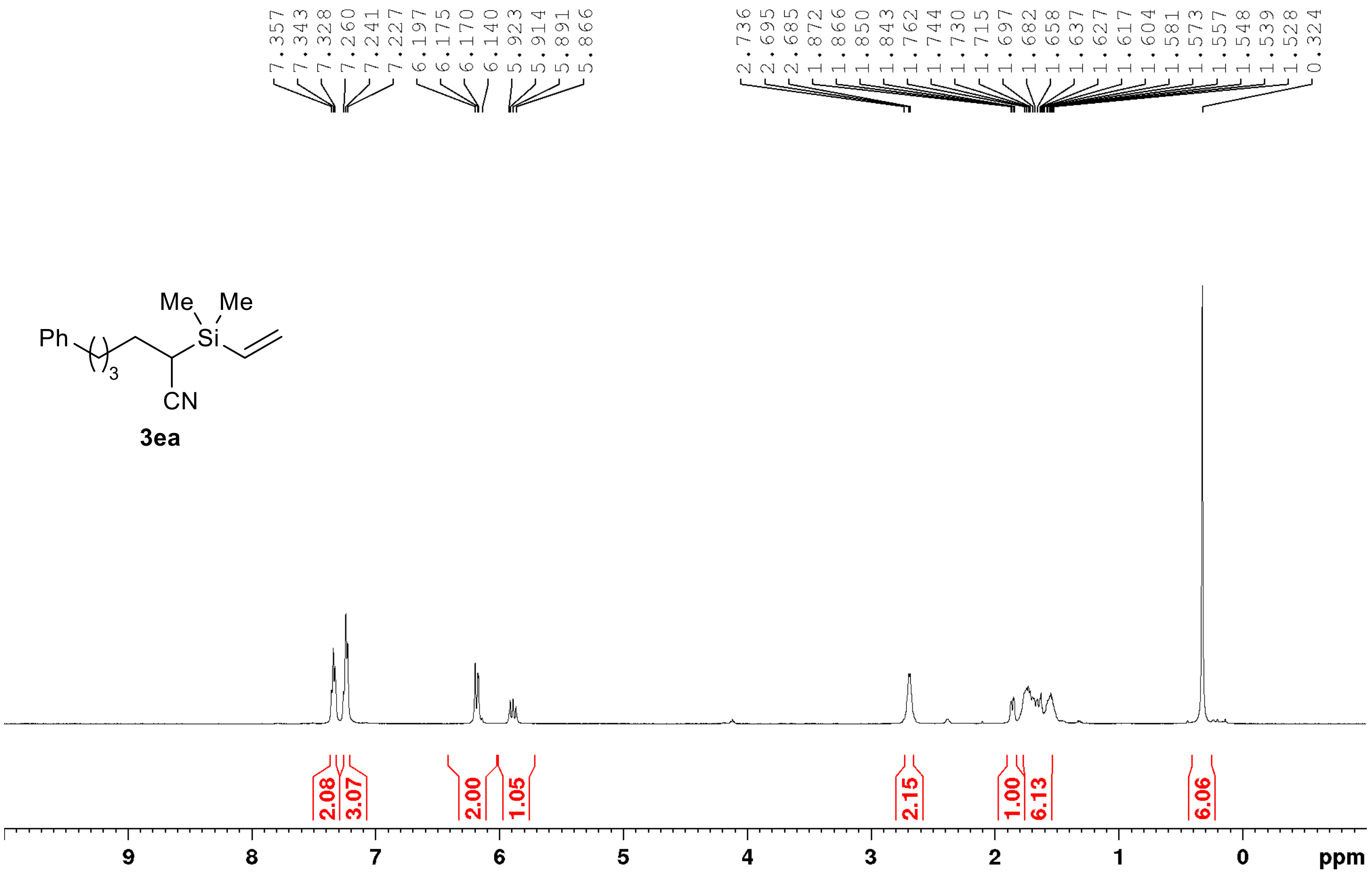

Figure S38. $^{13}C\{^1H\}$ **NMR** (125 MHz, $CDCl_3$, 298K) of **2-(Dimethyl(vinyl)silyl)-6-phenylhexanenitrile (3ea)**

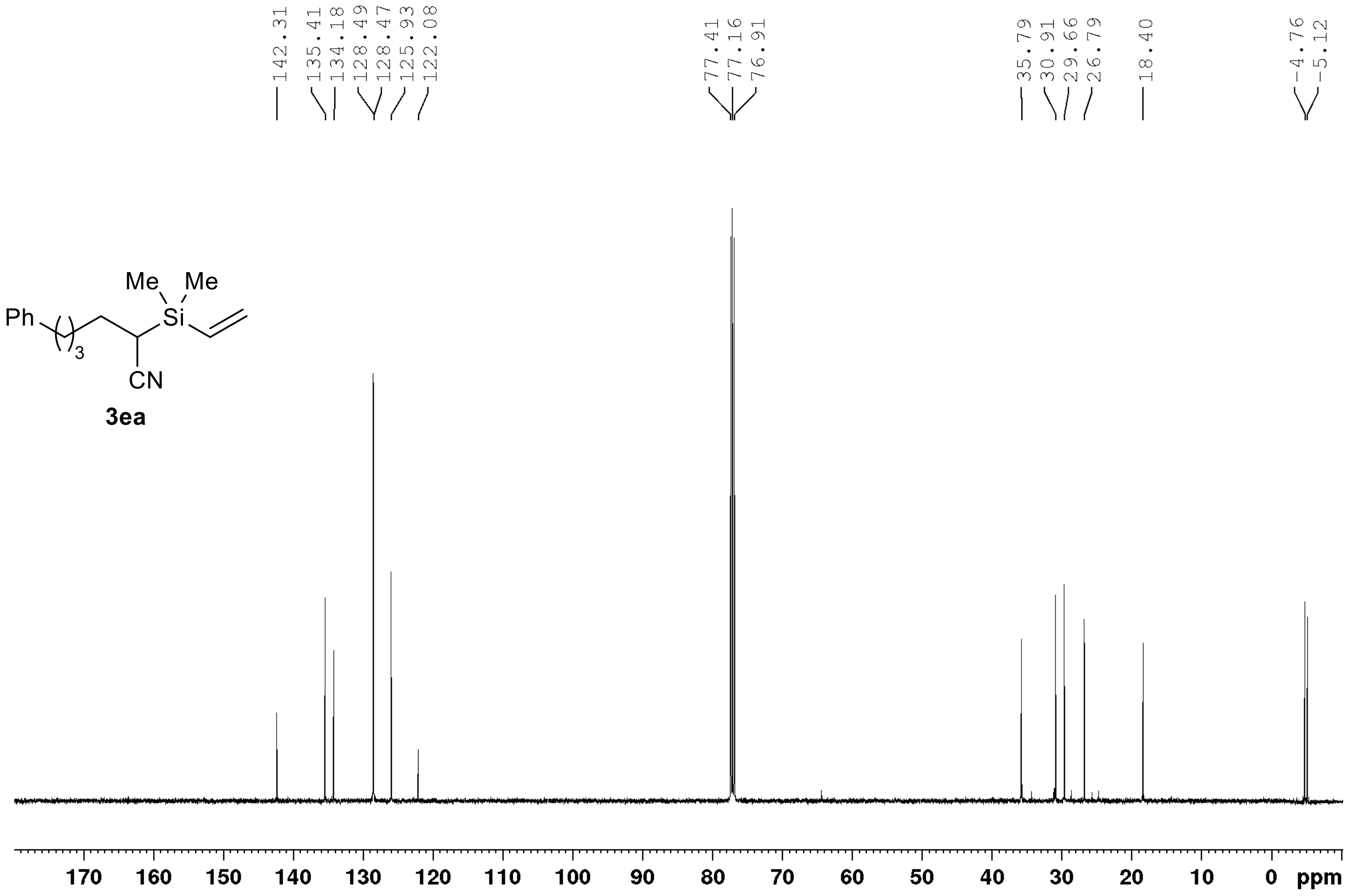

Figure S39. ^{29}Si{^{1}H} DEPT NMR (99 MHz, $CDCl_3$) of **2-(Dimethyl(vinyl)silyl)-6-phenylhexanenitrile (3ea)**

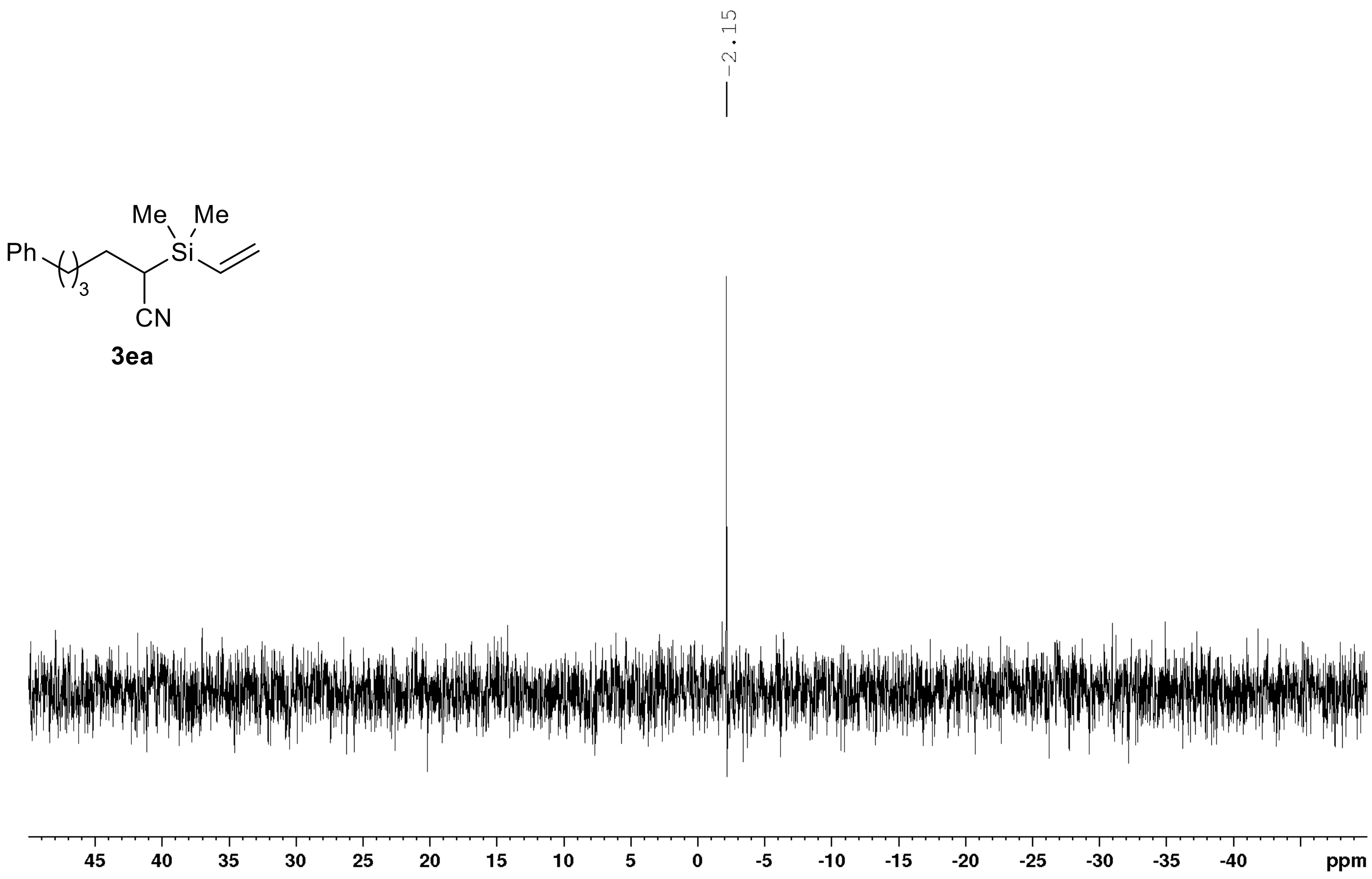

Figure S40. ^{1}H NMR (500 MHz, $CDCl_3$, 298K) of **7-Bromo-2-(dimethyl(vinyl)silyl)heptanenitrile (3fa)**

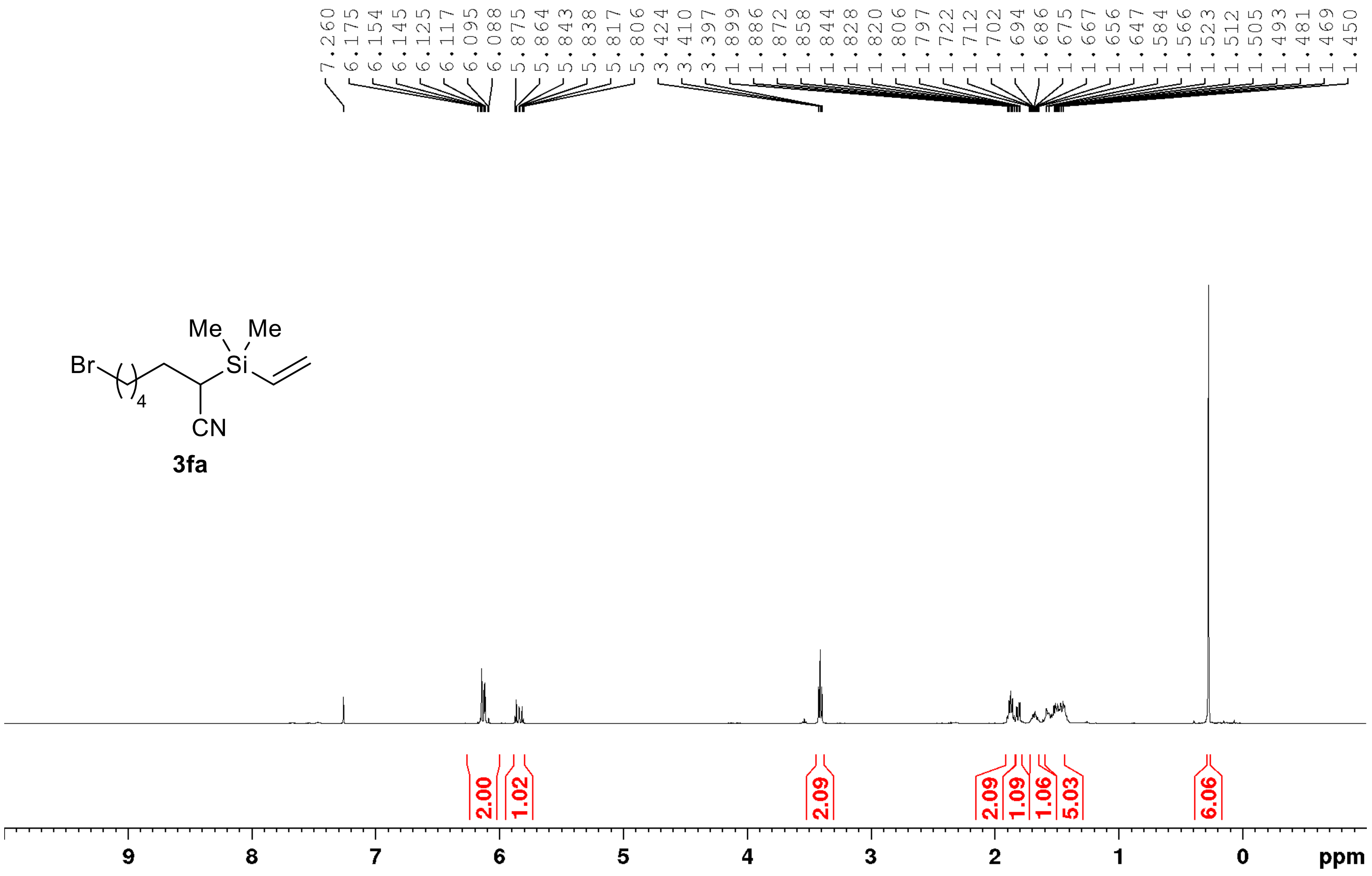

Figure S41. $^{13}C\{^{1}H\}$ **NMR** (125 MHz, $CDCl_3$, 298K) of **7-Bromo-2-(dimethyl(vinyl)silyl)heptanenitrile (3fa)**

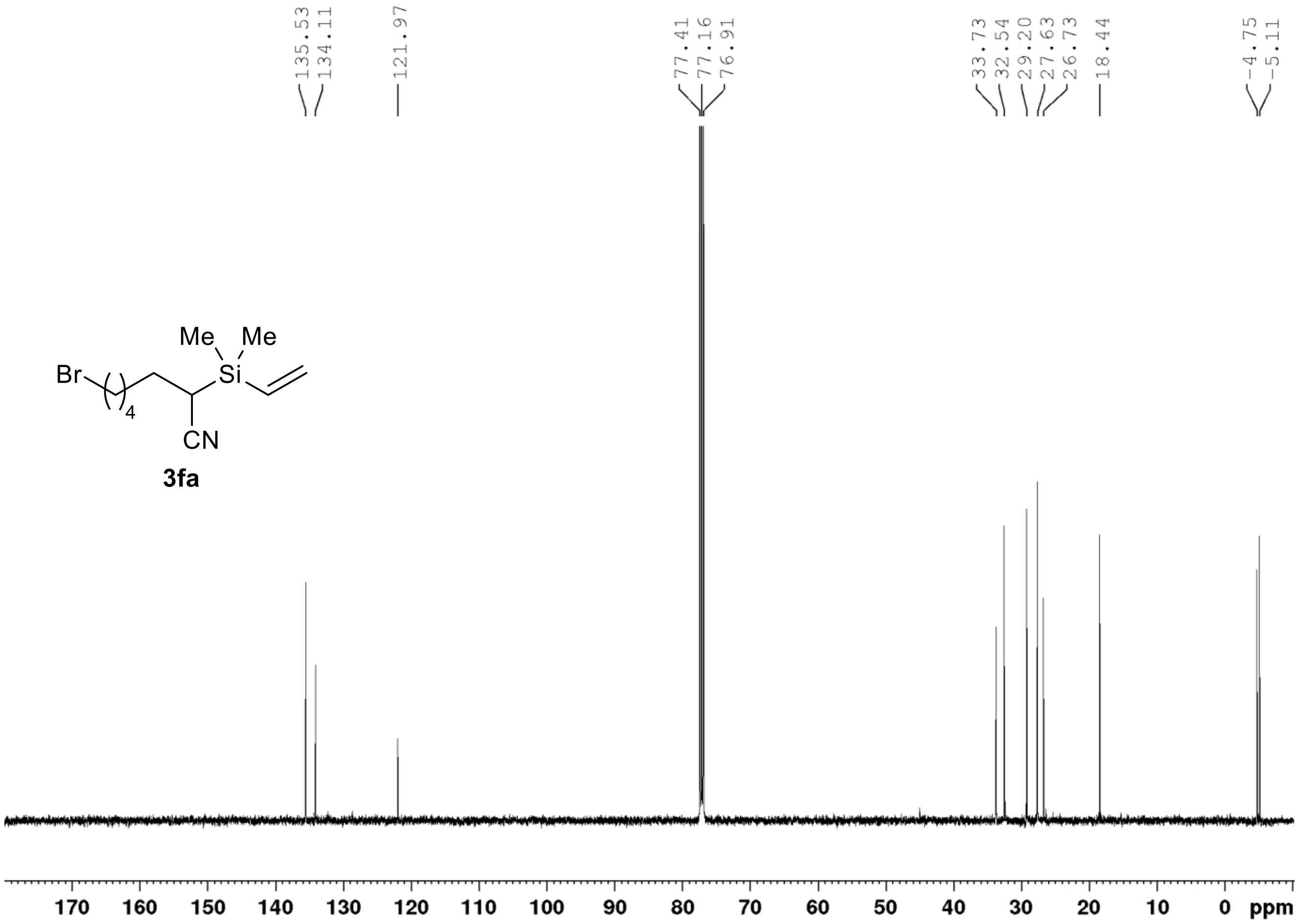

Figure S42. ^{29}Si{^{1}H} DEPT NMR (99 MHz, $CDCl_3$) of **7-Bromo-2-(dimethyl(vinyl)silyl)heptanenitrile (3fa)**

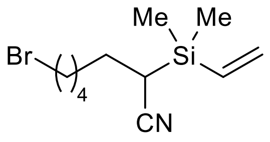

3fa

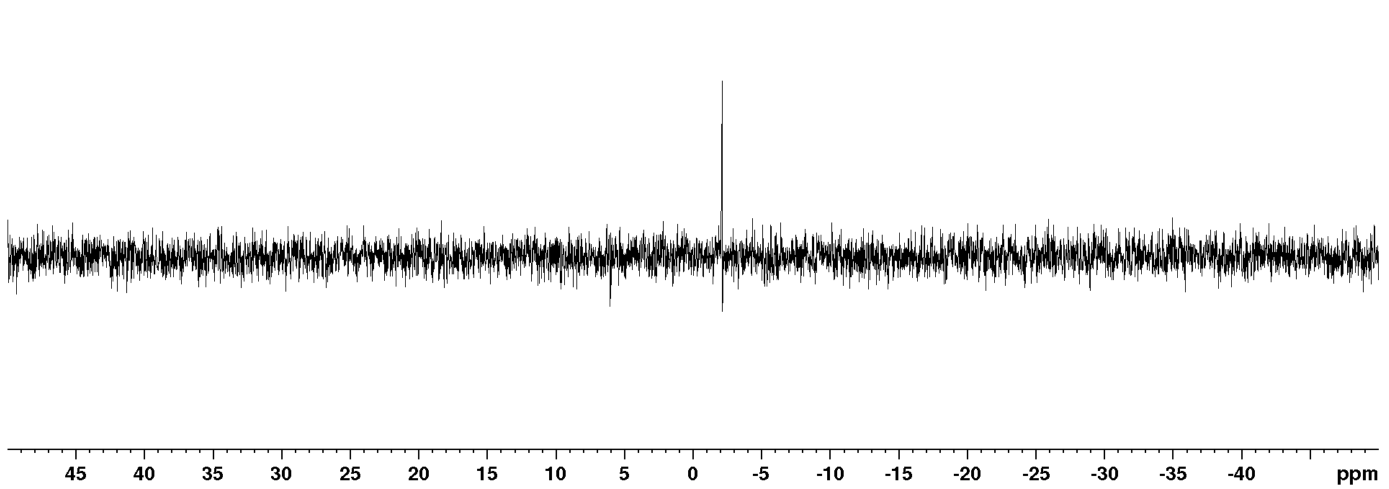

Figure S43. ^{1}H NMR (500 MHz, $CDCl_3$, 298K) of **Ethyl 6-cyano-6-(dimethyl(vinyl)silyl)hexanoate (3ga)** (with small amounts of unknown impurities in the aliphatic region)

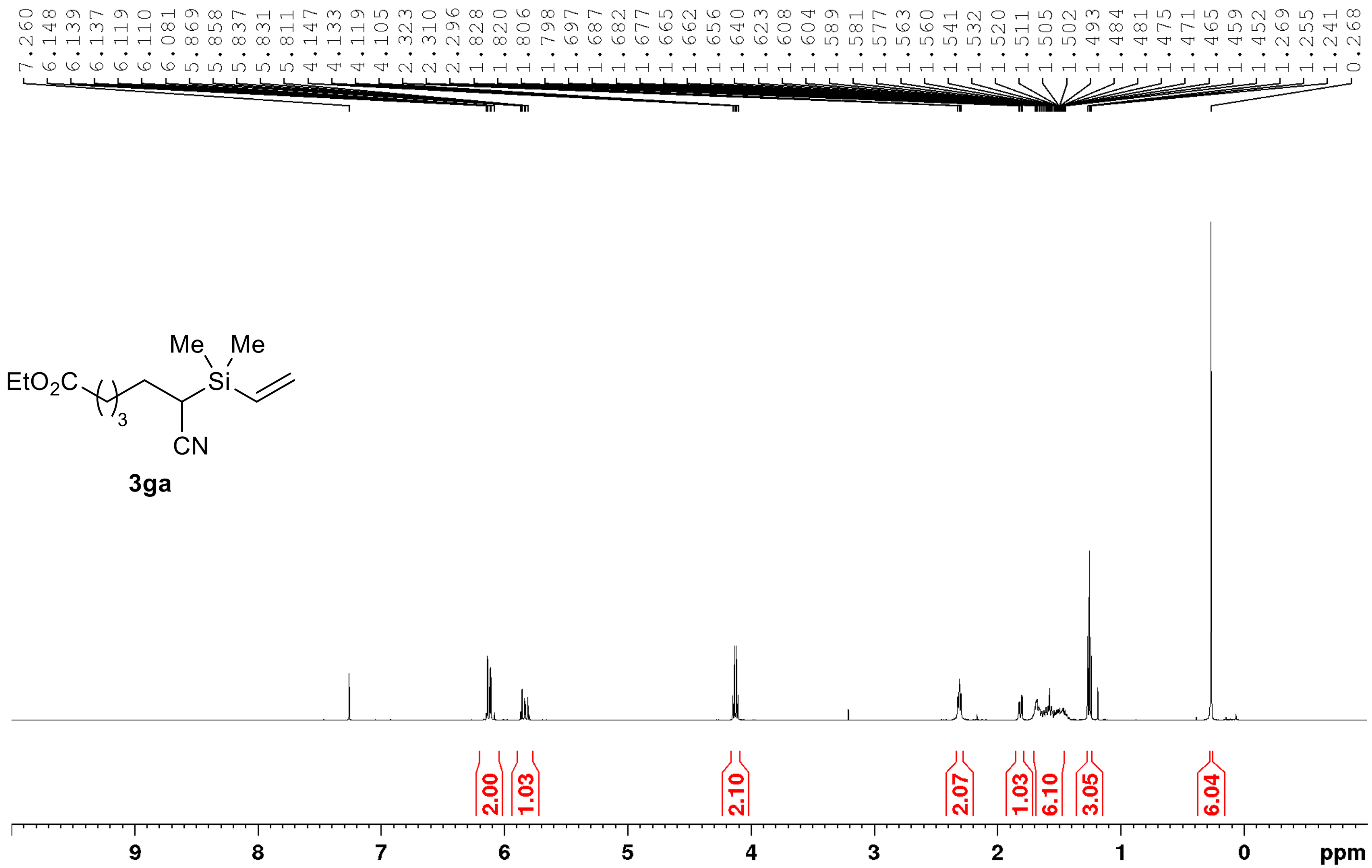

Figure S44. $^{13}C\{^1H\}$ **NMR** (125 MHz, $CDCl_3$, 298K) of **Ethyl 6-cyano-6-(dimethyl(vinyl)silyl)hexanoate (3ga)** (with small amounts of unknown impurities in the aliphatic region)

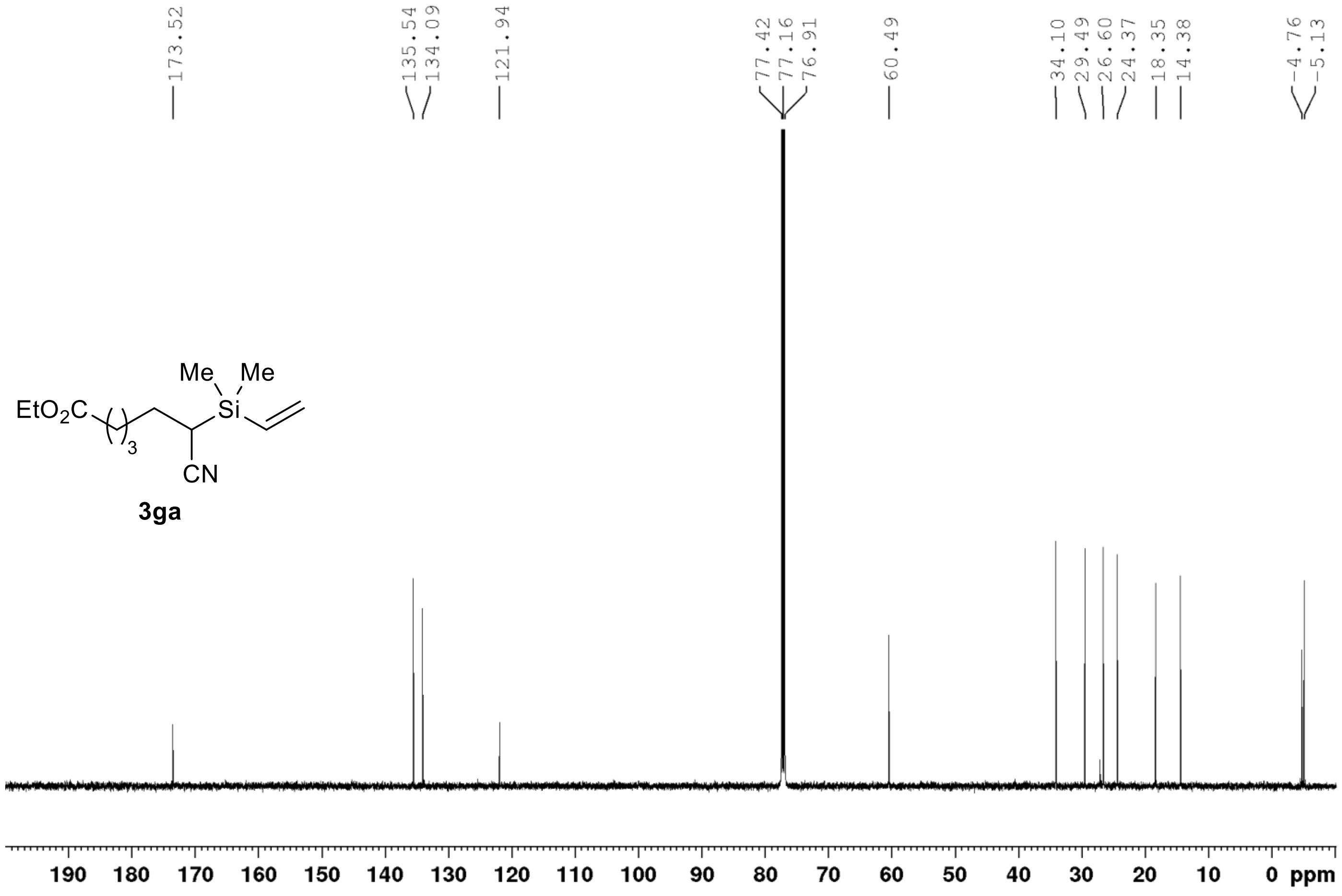

Figure S45. $^{1}H/^{29}Si$ HMQC NMR (500/99 MHz, $CDCl_3$, optimized for J = 7.0 Hz) of **Ethyl 6-cyano-6-(dimethyl(vinyl)silyl)hexanoate (3ga)**

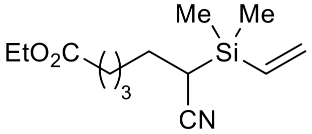

3ga

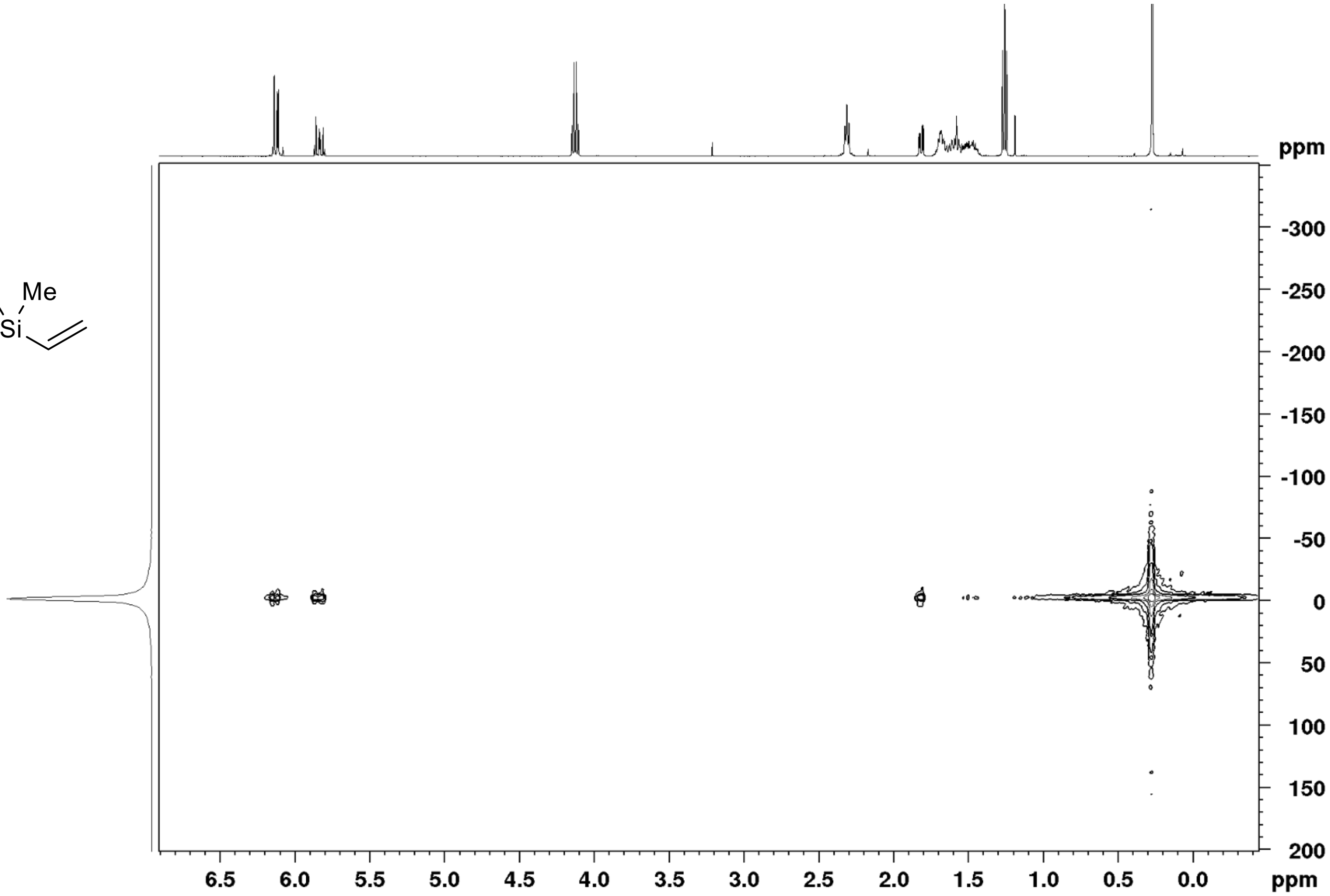

Figure S46. ^{1}**H NMR** (500 MHz, $CDCl_3$, 298K) of **4-Cyano-4-(dimethyl(vinyl)silyl)butyl benzoate (3ha)**

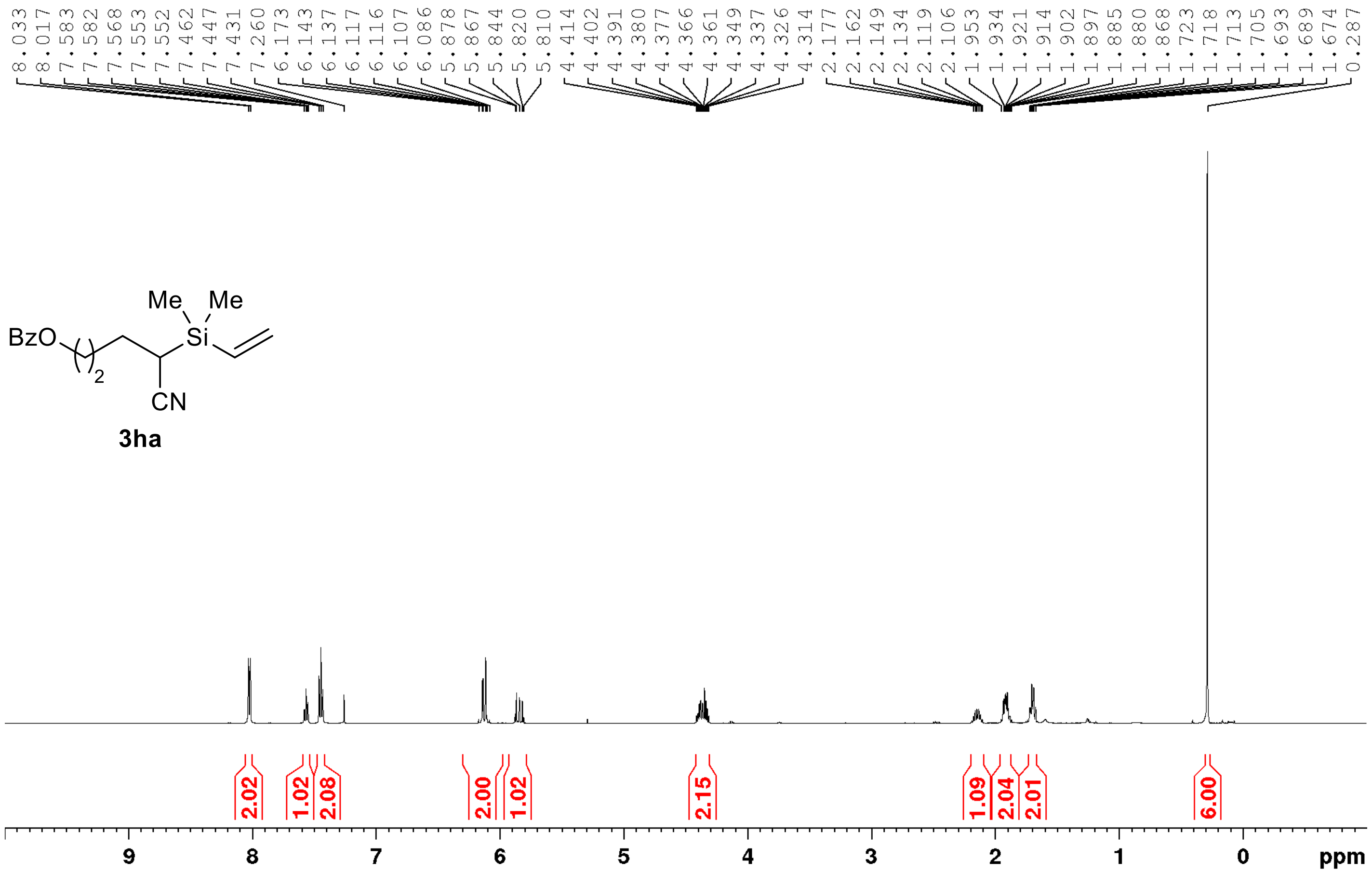

Figure S47. $^{13}C\{^1H\}$ NMR (125 MHz, $CDCl_3$, 298K) of **4-Cyano-4-(dimethyl(vinyl)silyl)butyl benzoate (3ha)**

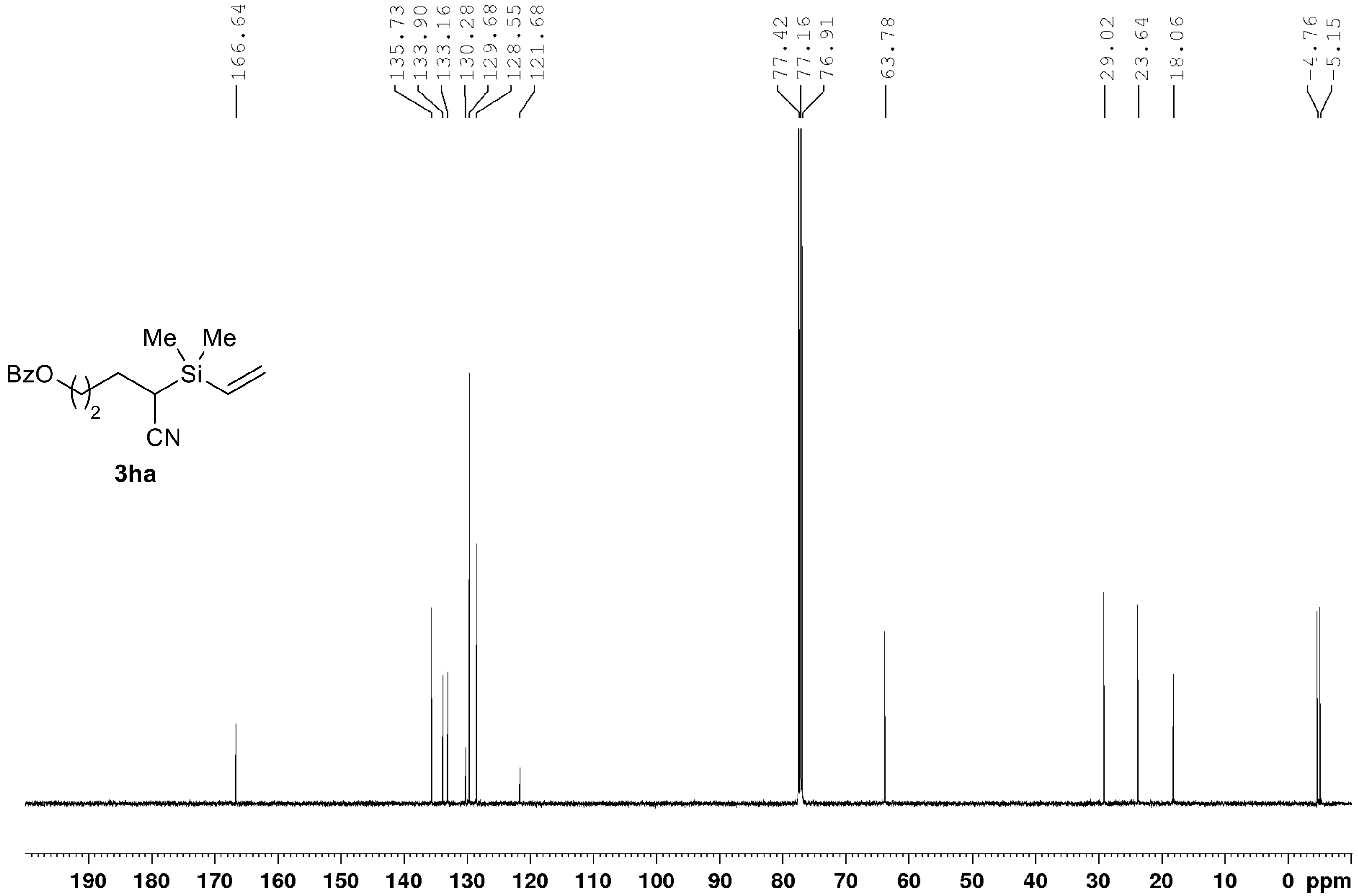

Figure S48. ^{29}Si{^{1}H} DEPT NMR (99 MHz, $CDCl_3$) of **4-Cyano-4-(dimethyl(vinyl)silyl)butyl benzoate (3ha)**

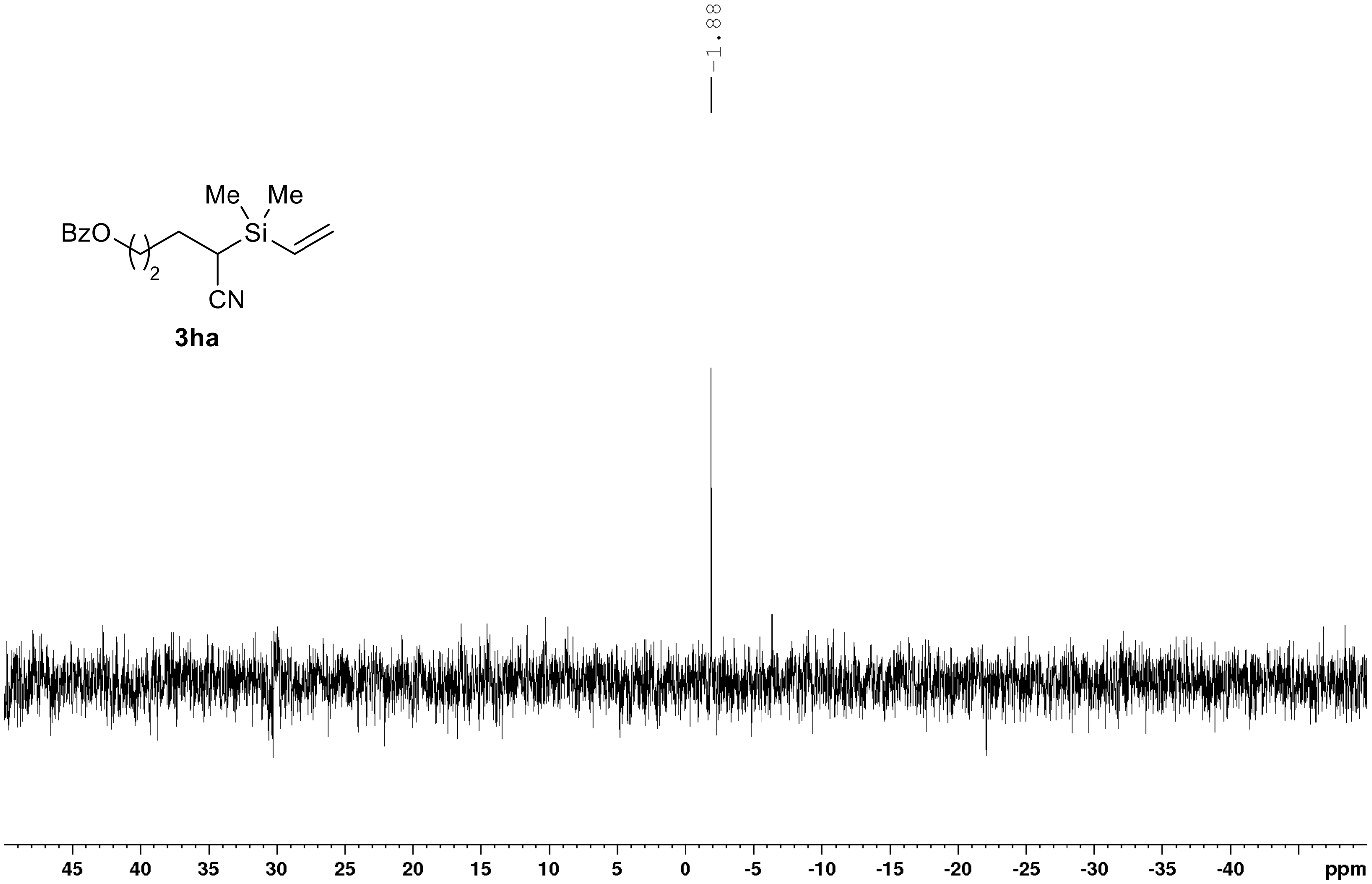

Figure S49. **^{1}H NMR** (500 MHz, $CDCl_3$, 298K) of **4-Cyano-4-(dimethyl(vinyl)silyl)butyl pivalate (3ia)**

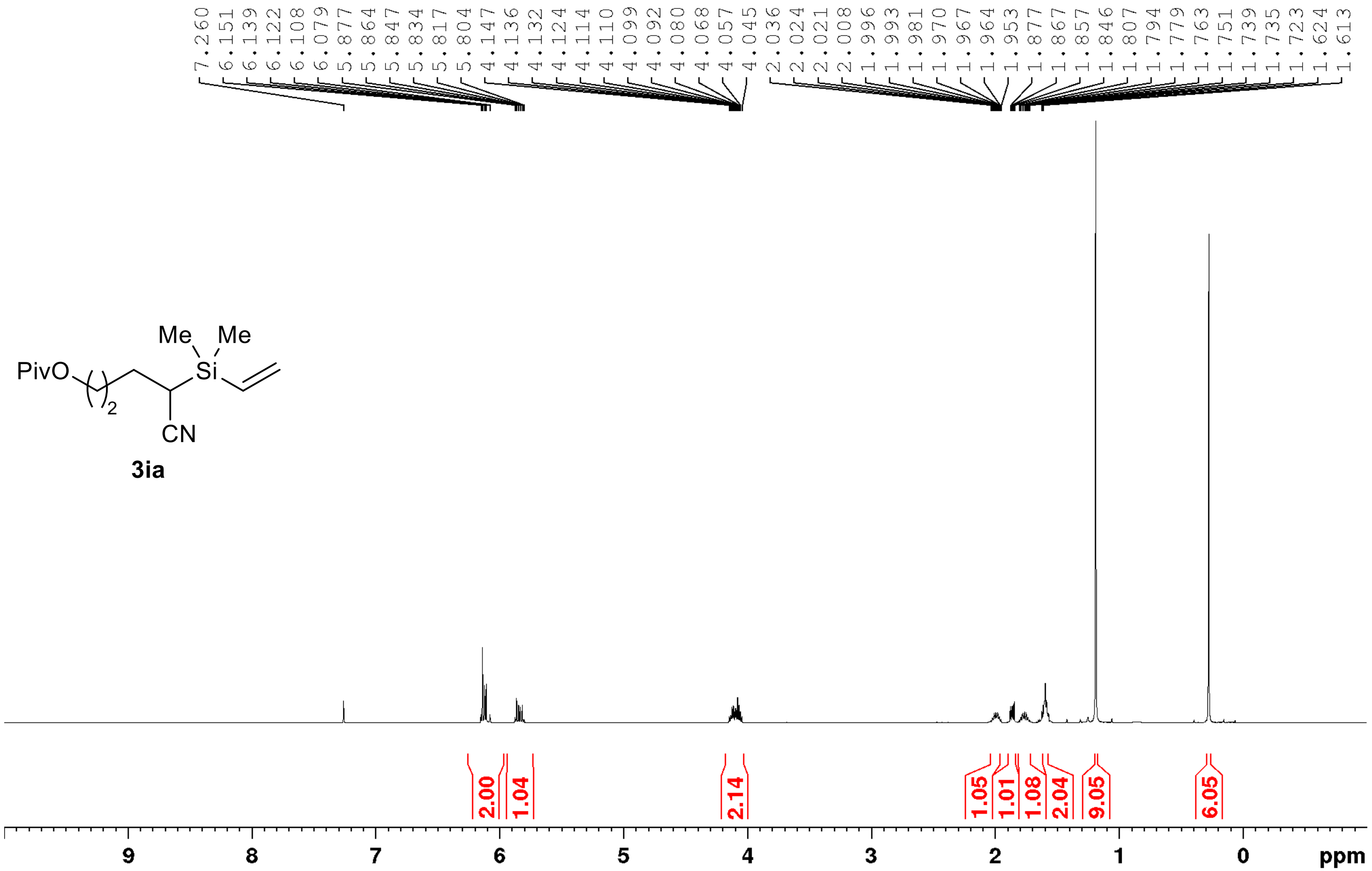

Figure S50. $^{13}C\{^{1}H\}$ **NMR** (125 MHz, $CDCl_3$, 298K) of **4-Cyano-4-(dimethyl(vinyl)silyl)butyl pivalate (3ia)**

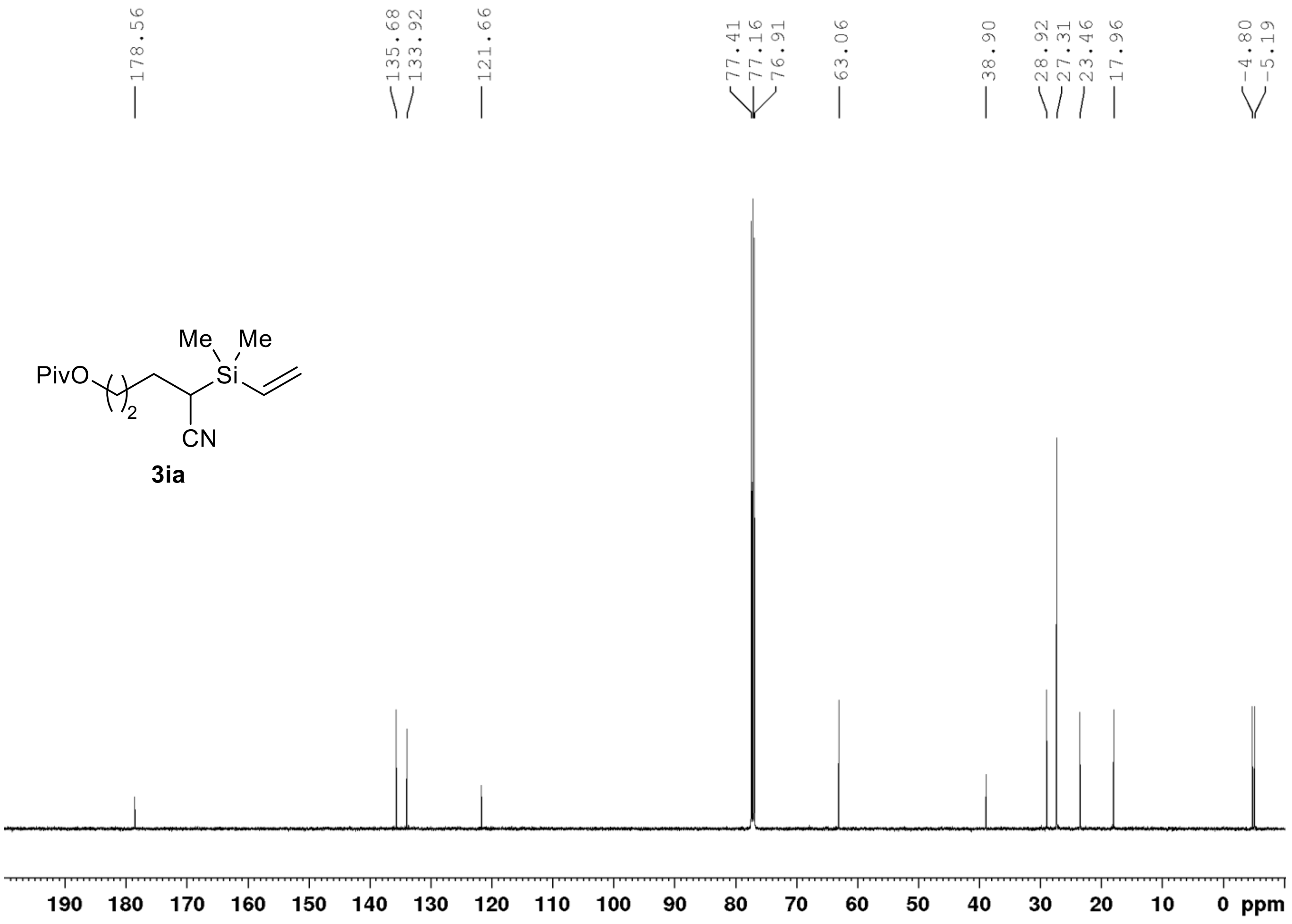

Figure S51. ^{29}Si{^{1}H} DEPT NMR (99 MHz, $CDCl_3$) of **4-Cyano-4-(dimethyl(vinyl)silyl)butyl pivalate (3ia)**

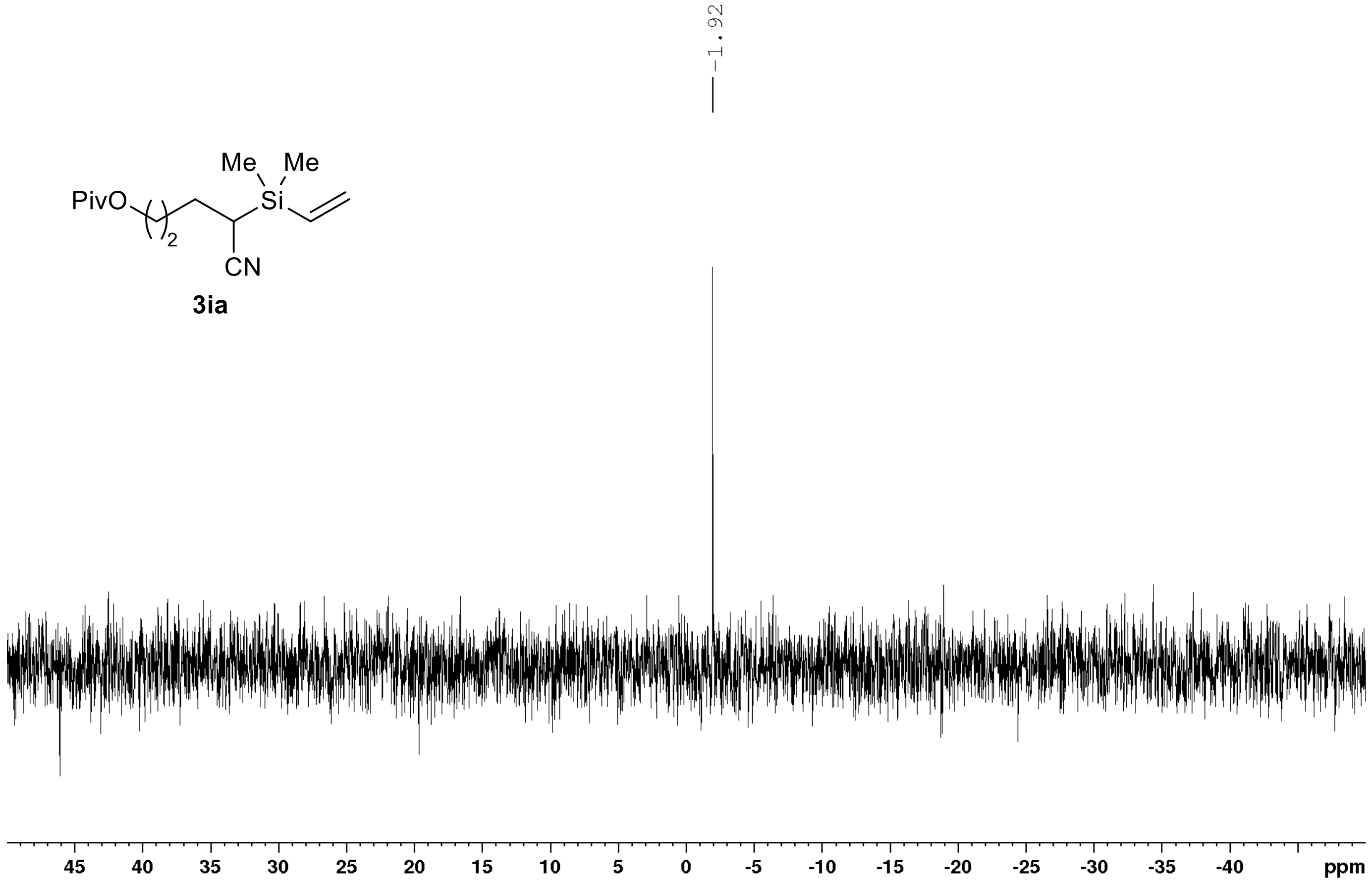

Figure S52. **^{1}H NMR** (500 MHz, $CDCl_3$, 298K) of **2-(Dimethyl(vinyl)silyl)hept-6-enenitrile (3ja)**

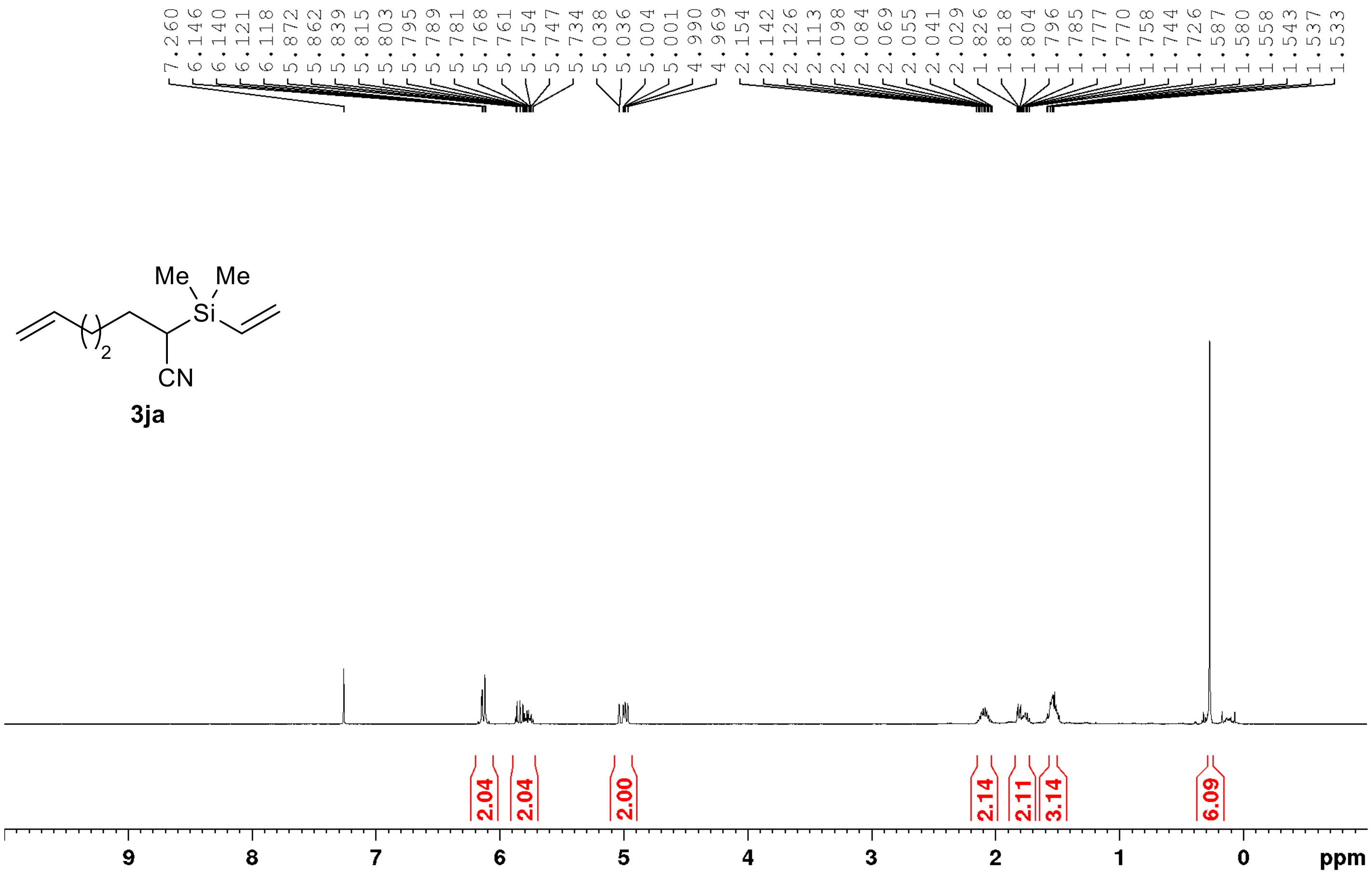

Figure S53. $^{13}C\{^1H\}$ **NMR** (125 MHz, $CDCl_3$, 298K) of **2-(Dimethyl(vinyl)silyl)hept-6-enenitrile (3ja)**

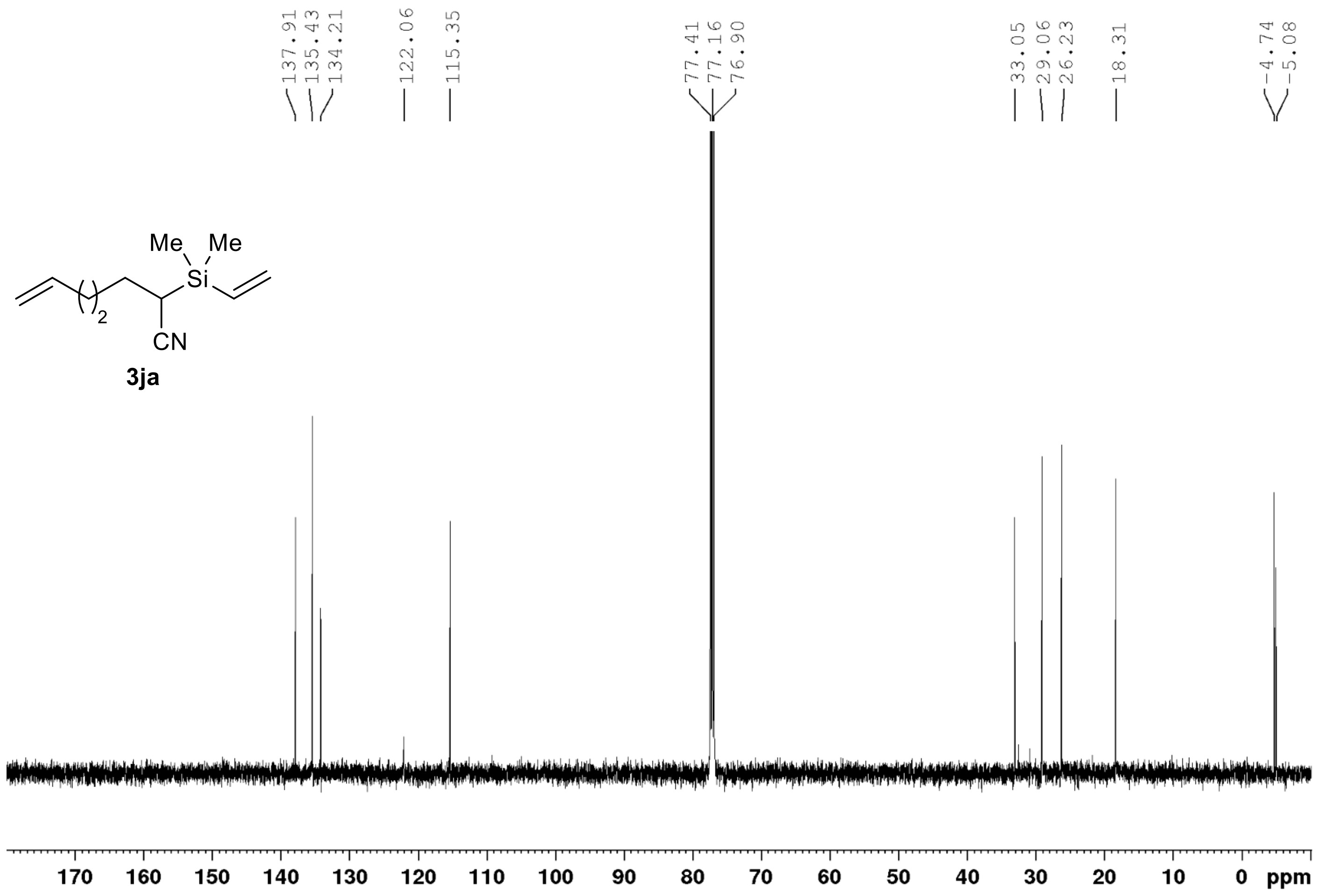

Figure S54. ^{1}H/^{29}Si HMQC NMR (500/99 MHz, $CDCl_3$, optimized for J = 7.0 Hz) of **2-(Dimethyl(vinyl)silyl)hept-6-enenitrile (3ja)**

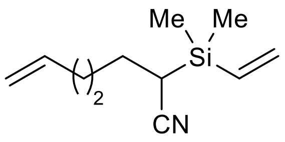

3ja

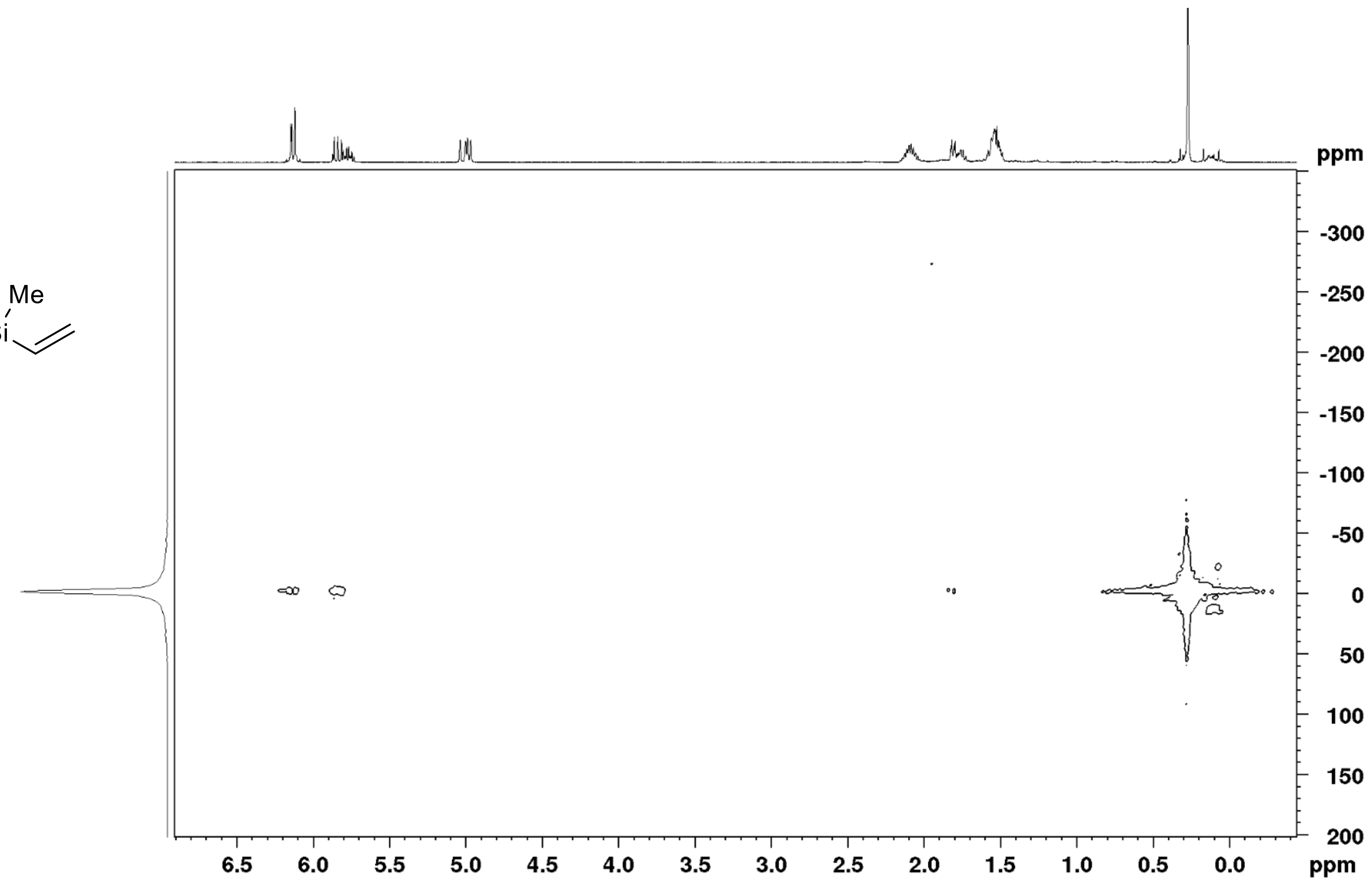

Figure S55. ^{1}H NMR (500 MHz, $CDCl_3$, 298K) of **4-((6-Cyano-6-(dimethyl(vinyl)silyl)hexyl)oxy)benzonitrile (3ka)**

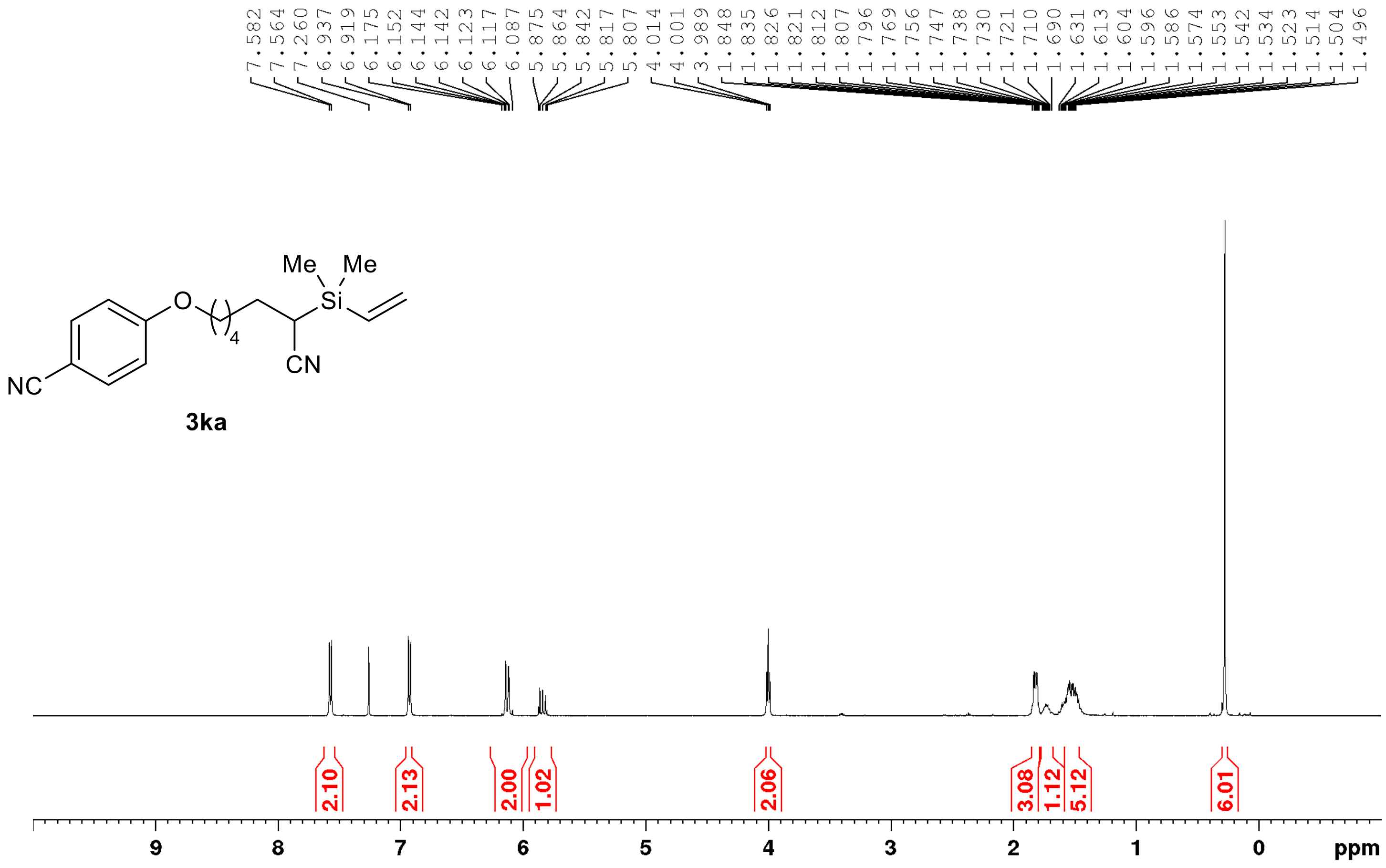

Figure S56. $^{13}C\{^1H\}$ NMR (125 MHz, $CDCl_3$, 298K) of **4-((6-Cyano-6-(dimethyl(vinyl)silyl)hexyl)oxy)benzonitrile (3ka)**

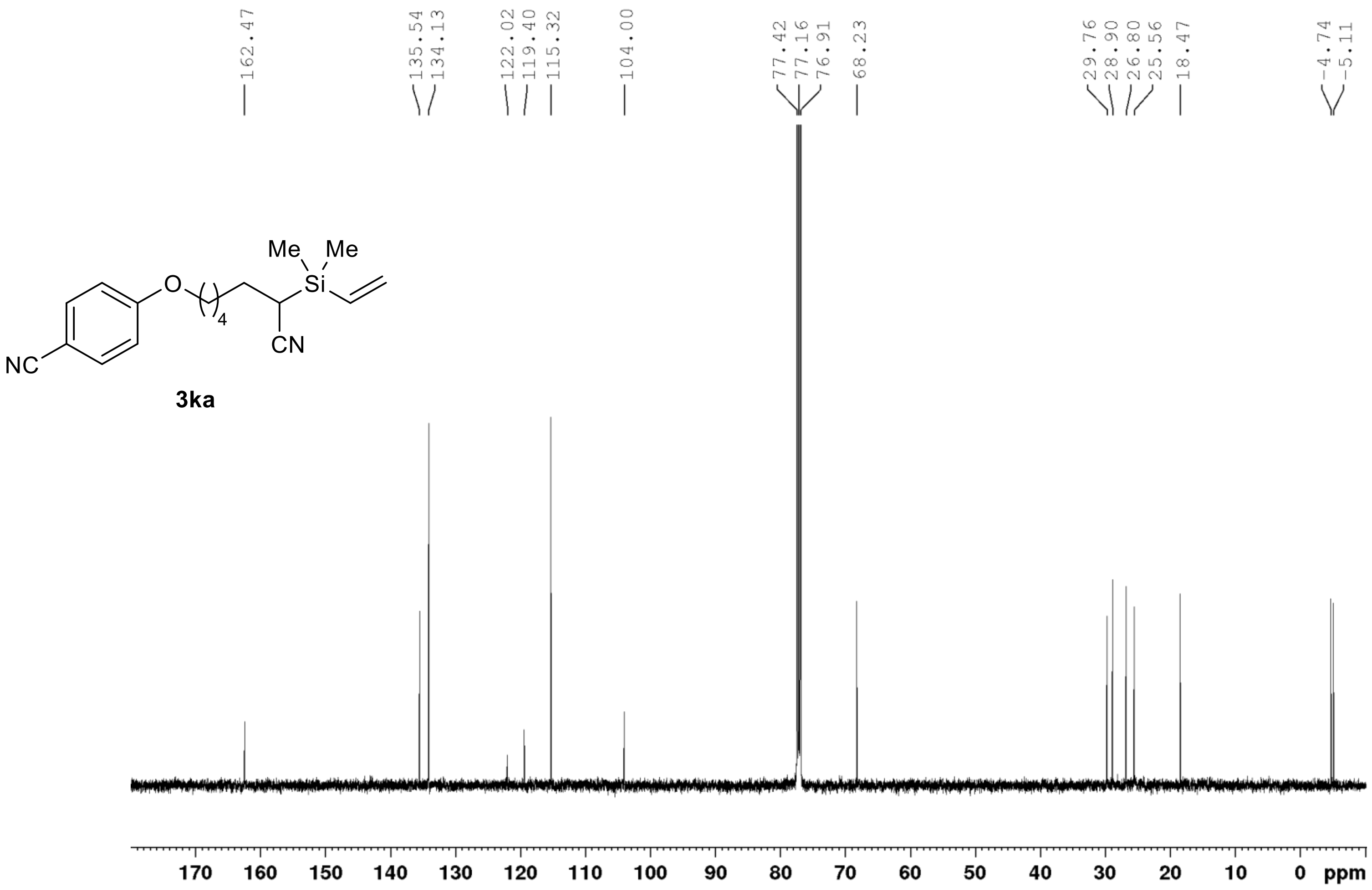

3ka

Figure S57. $^{1}H/^{29}Si$ **HMQC NMR** (500/99 MHz, $CDCl_3$, optimized for J = 7.0 Hz) of **4-((6-Cyano-6-(dimethyl(vinyl)silyl)hexyl)oxy)benzonitrile (3ka)**

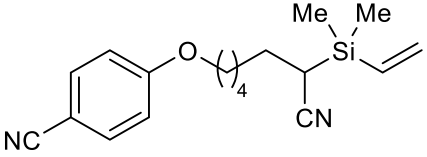

3ka

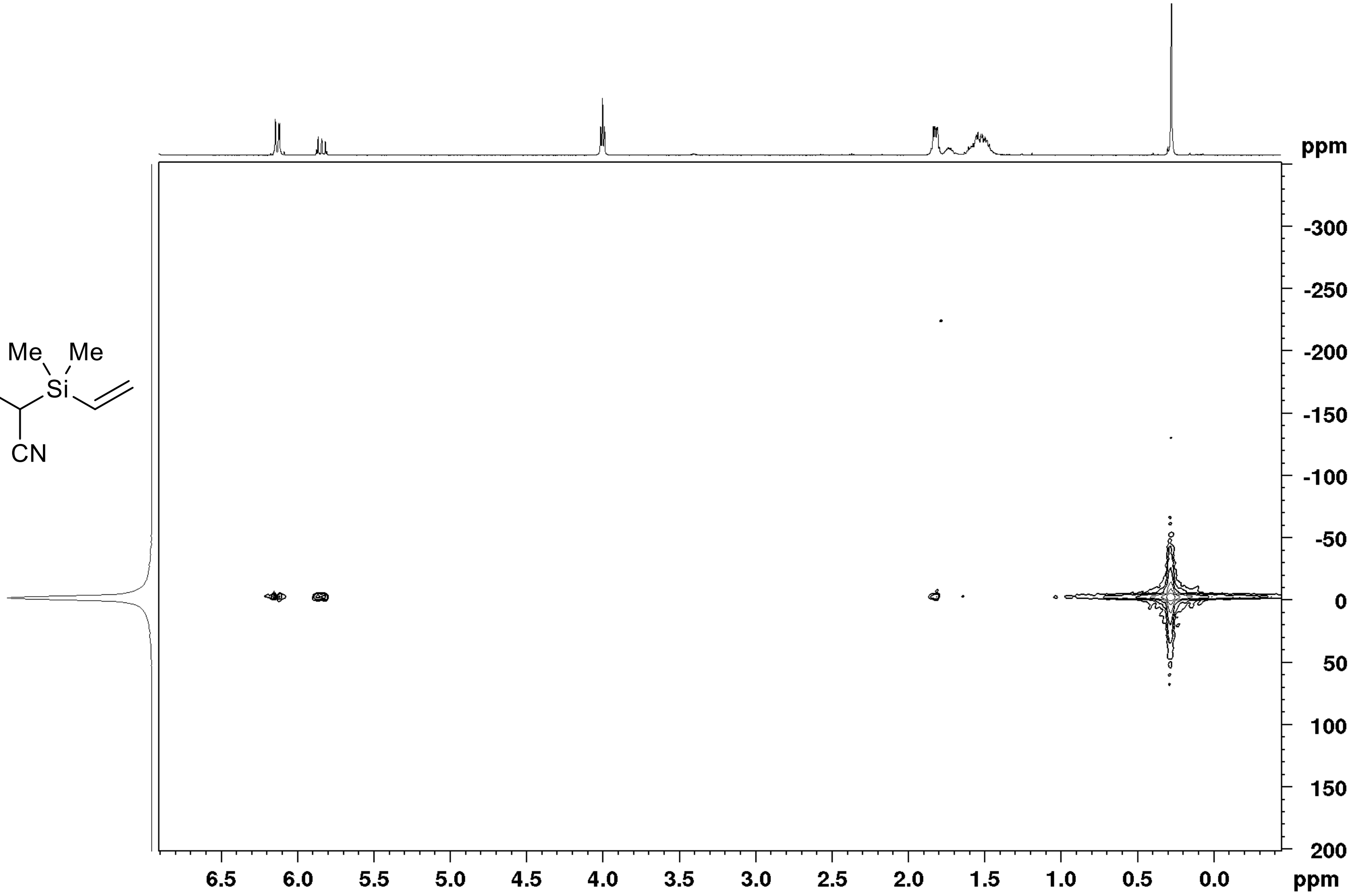

Figure S58. ^{1}H NMR (500 MHz, $CDCl_3$, 298K) of **2-(Cyclohex-2-en-1-yl)-2-(dimethyl(vinyl)silyl)acetonitrile (3la)**
(with small amounts of unknown impurities in the aliphatic region)

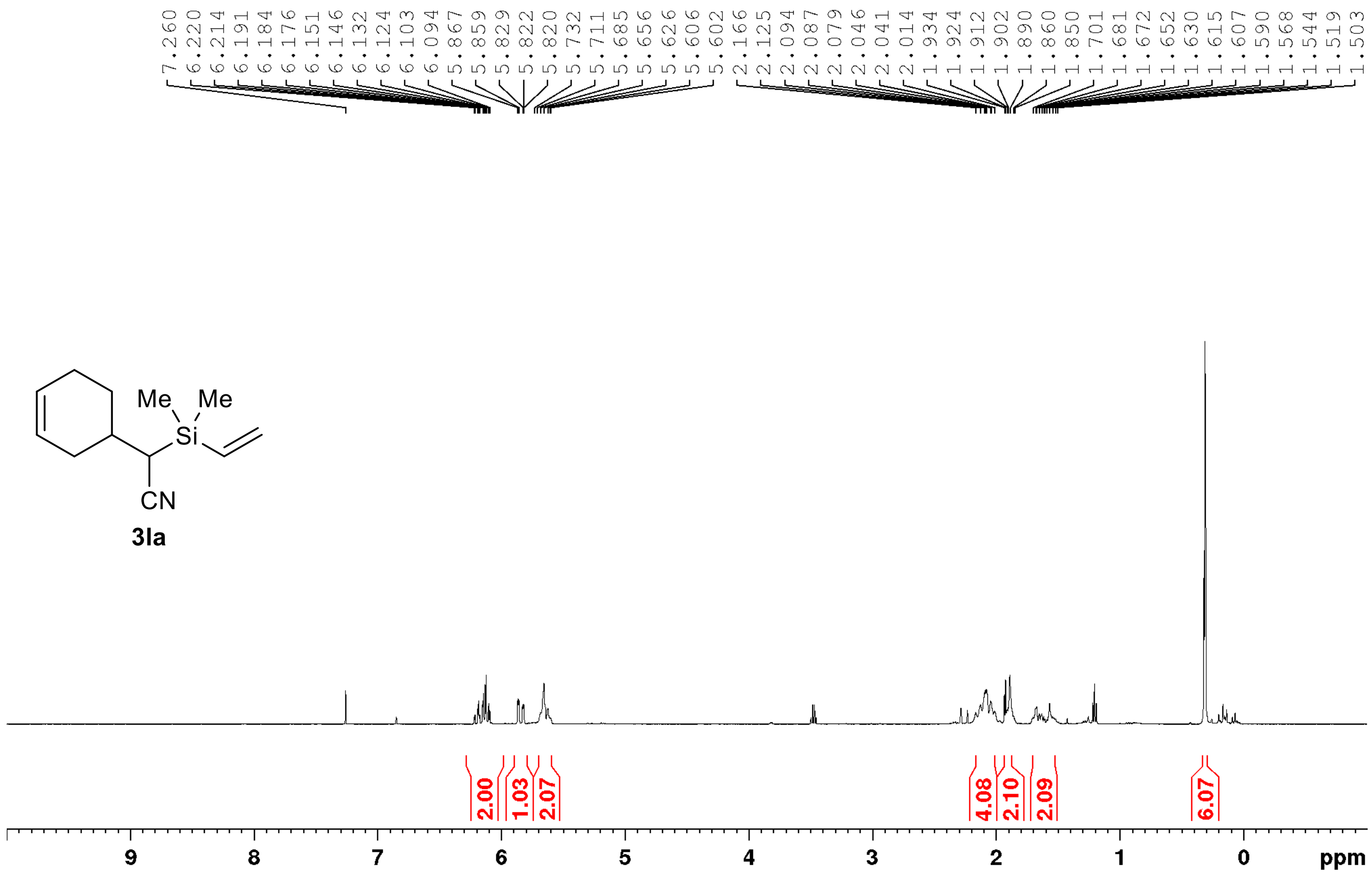

Figure S59. $^{13}C\{^{1}H\}$ **NMR** (125 MHz, $CDCl_3$, 298K) of **2-(Cyclohex-2-en-1-yl)-2-(dimethyl(vinyl)silyl)acetonitrile (3la)** (with small amounts of unknown impurities in the aliphatic region)

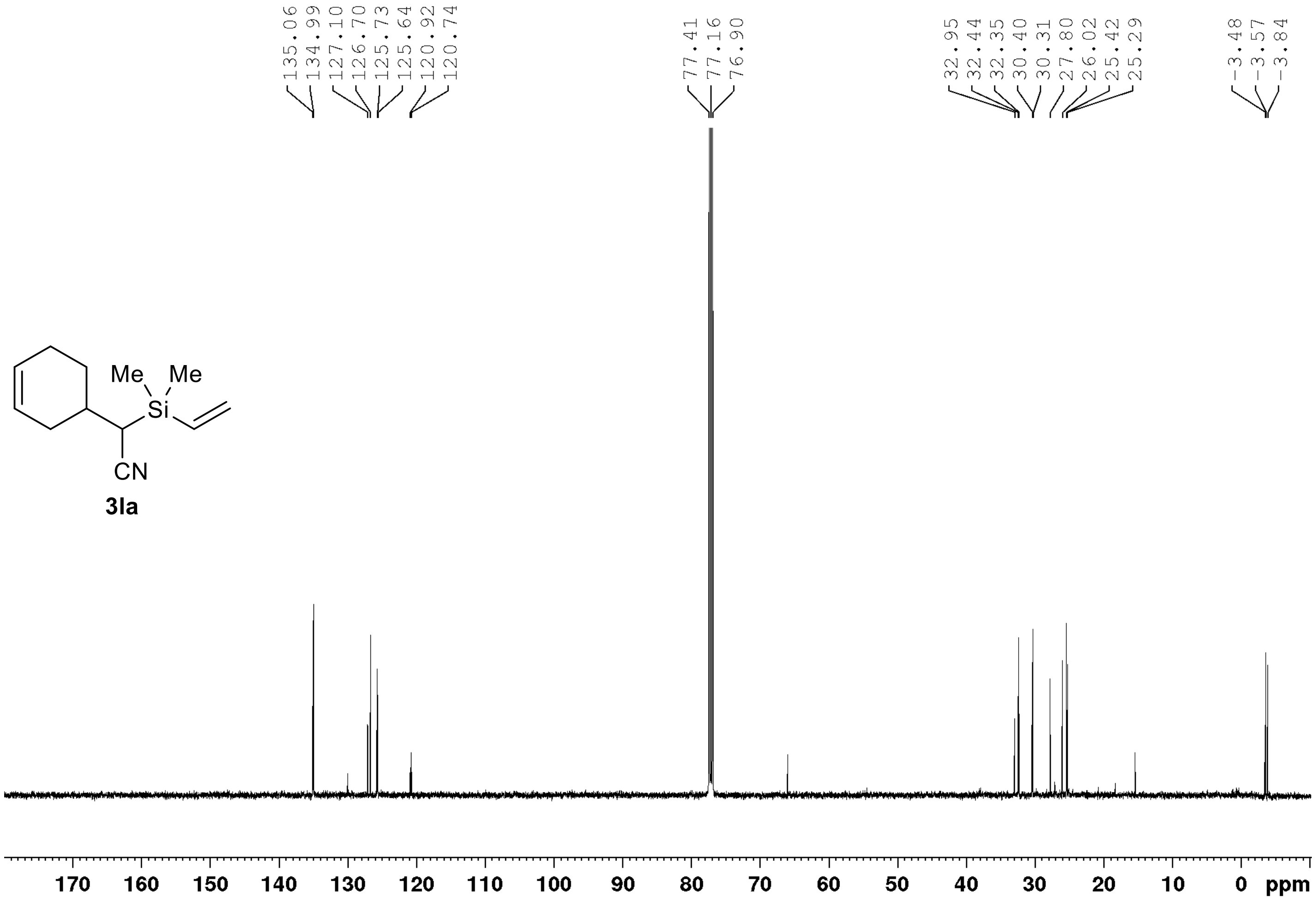

Figure S60. ^{29}**Si{**^{1}**H} DEPT NMR** (99 MHz, $CDCl_3$) of **2-(Cyclohex-2-en-1-yl)-2-(dimethyl(vinyl)silyl)acetonitrile (3la)**

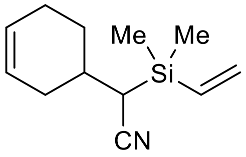

3la

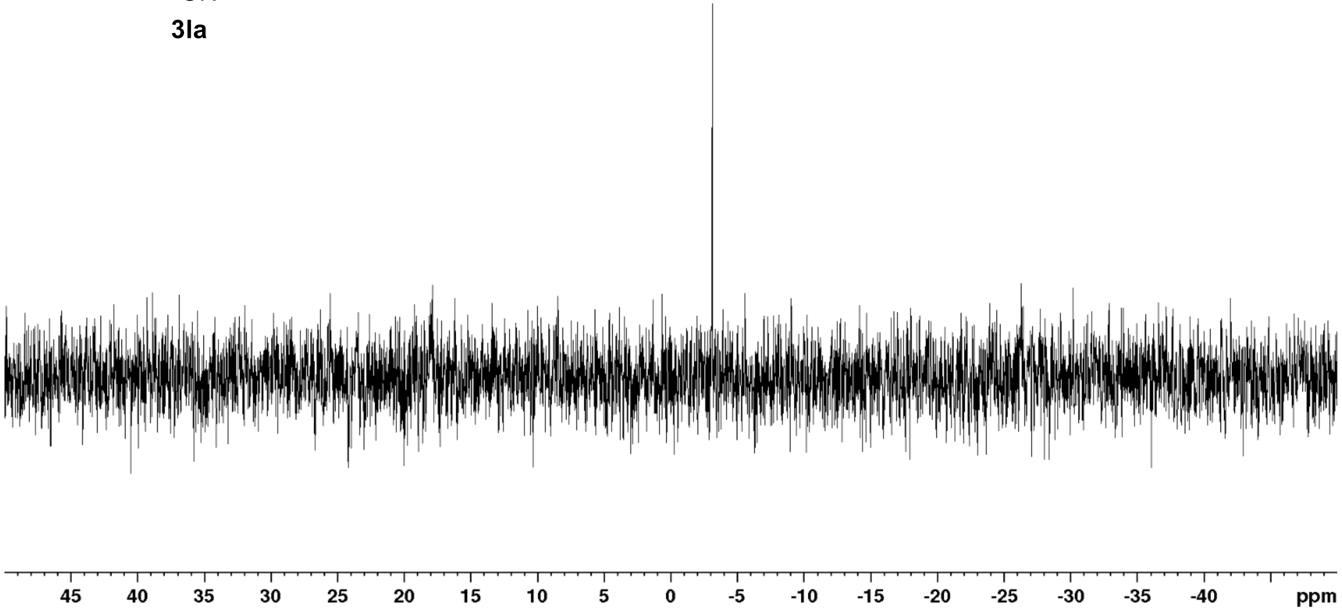

Figure S61. ^{1}H NMR (500 MHz, $CDCl_3$, 298K) of **2-(Dimethyl(vinyl)silyl)octanenitrile (3ma)**
(with small amounts of unknown impurities in the aliphatic region)

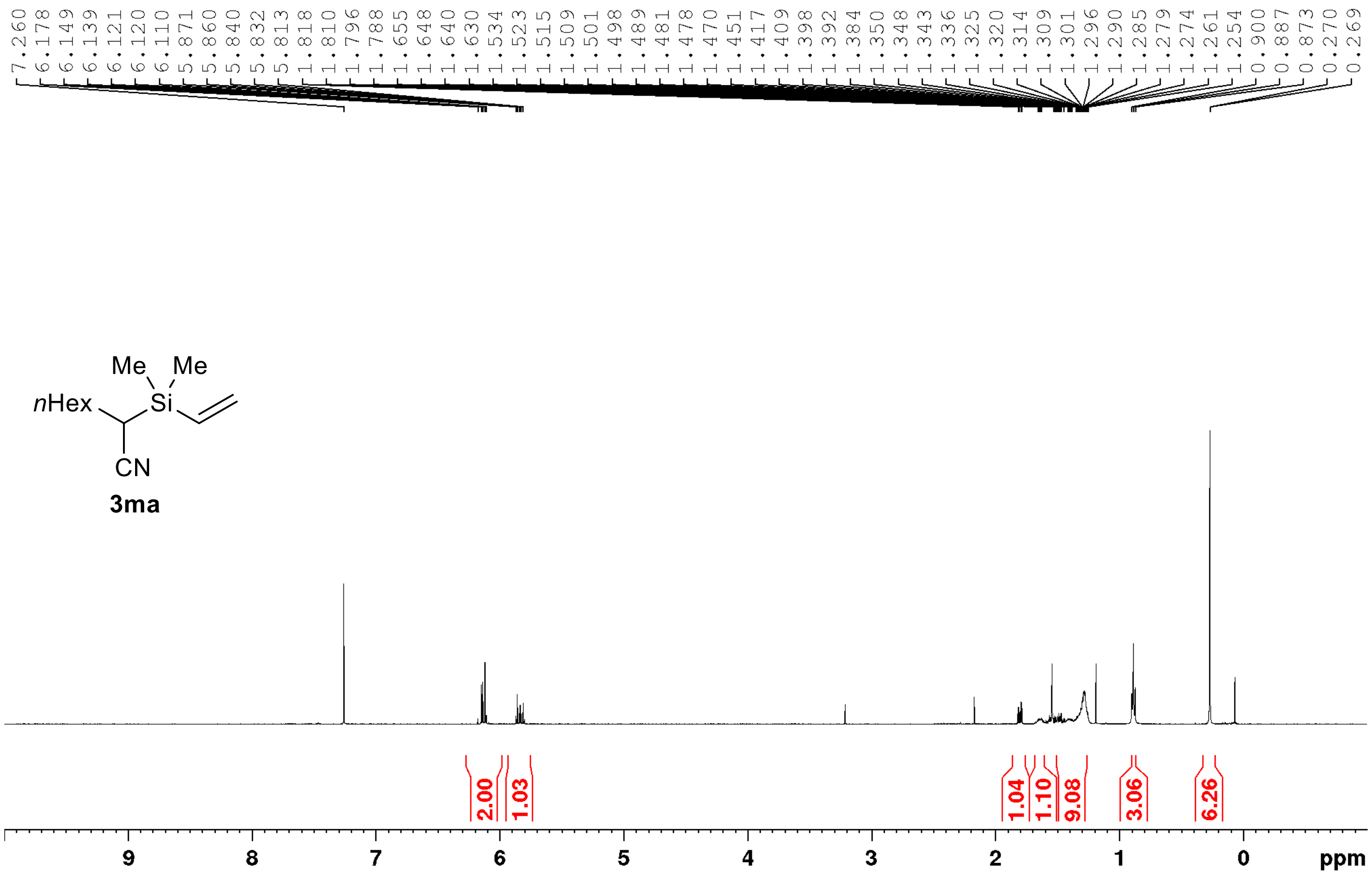

Figure S62. ^{13}C{^{1}H} NMR (125 MHz, $CDCl_3$, 298K) of **2-(Dimethyl(vinyl)silyl)octanenitrile (3ma)** (with small amounts of unknown impurities in the aliphatic region)

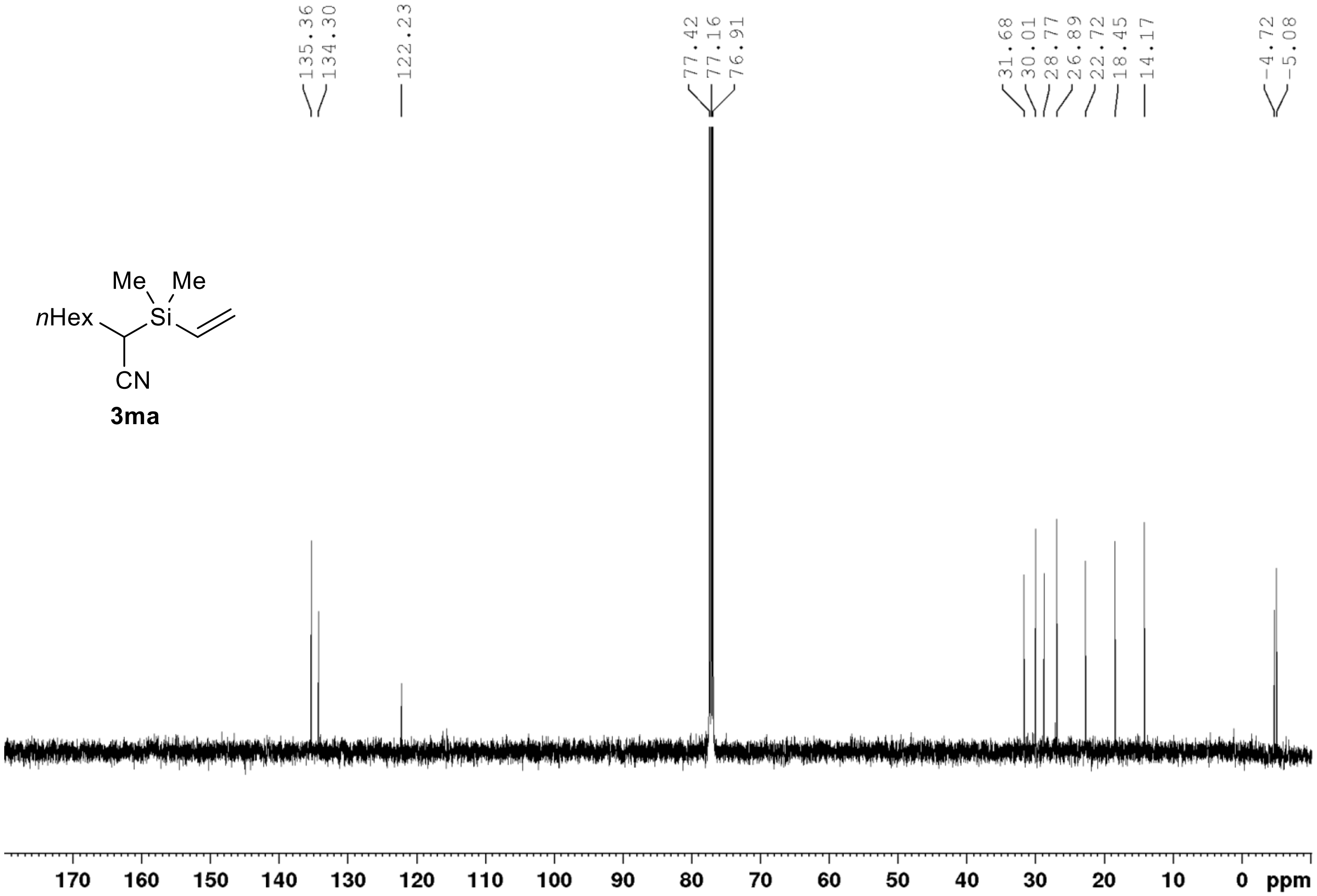

Figure S63. $^{1}H/^{29}Si$ HMQC NMR (500/99 MHz, $CDCl_3$, optimized for J = 7.0 Hz) of **2-(Dimethyl(vinyl)silyl)octanenitrile (3ma)**

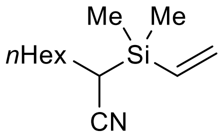

3ma

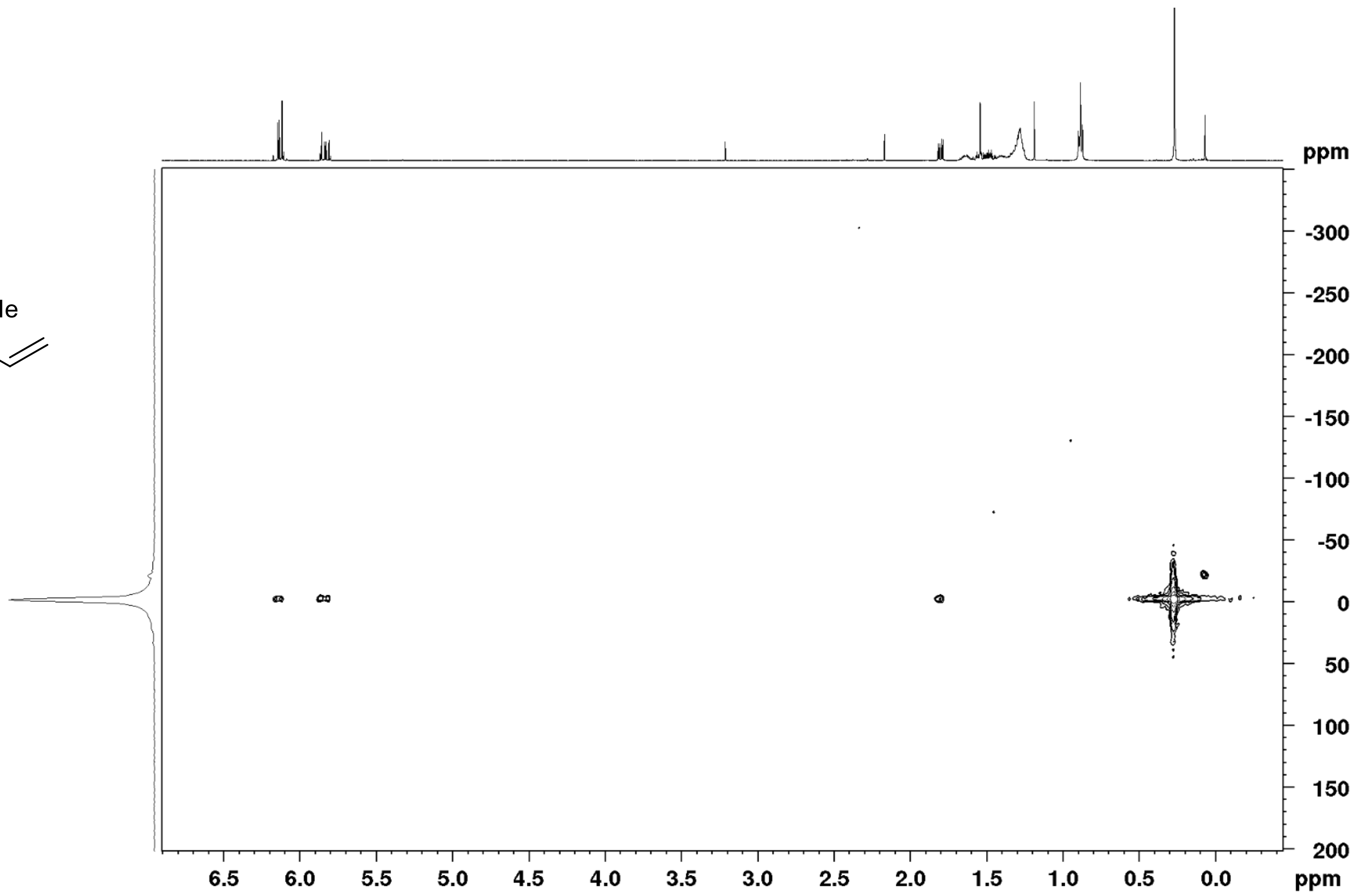

Figure S64. **^{1}H NMR** (500 MHz, $CDCl_3$, 298K) of **2-Cyclopentyl-2-(dimethyl(vinyl)silyl)acetonitrile (3na)**

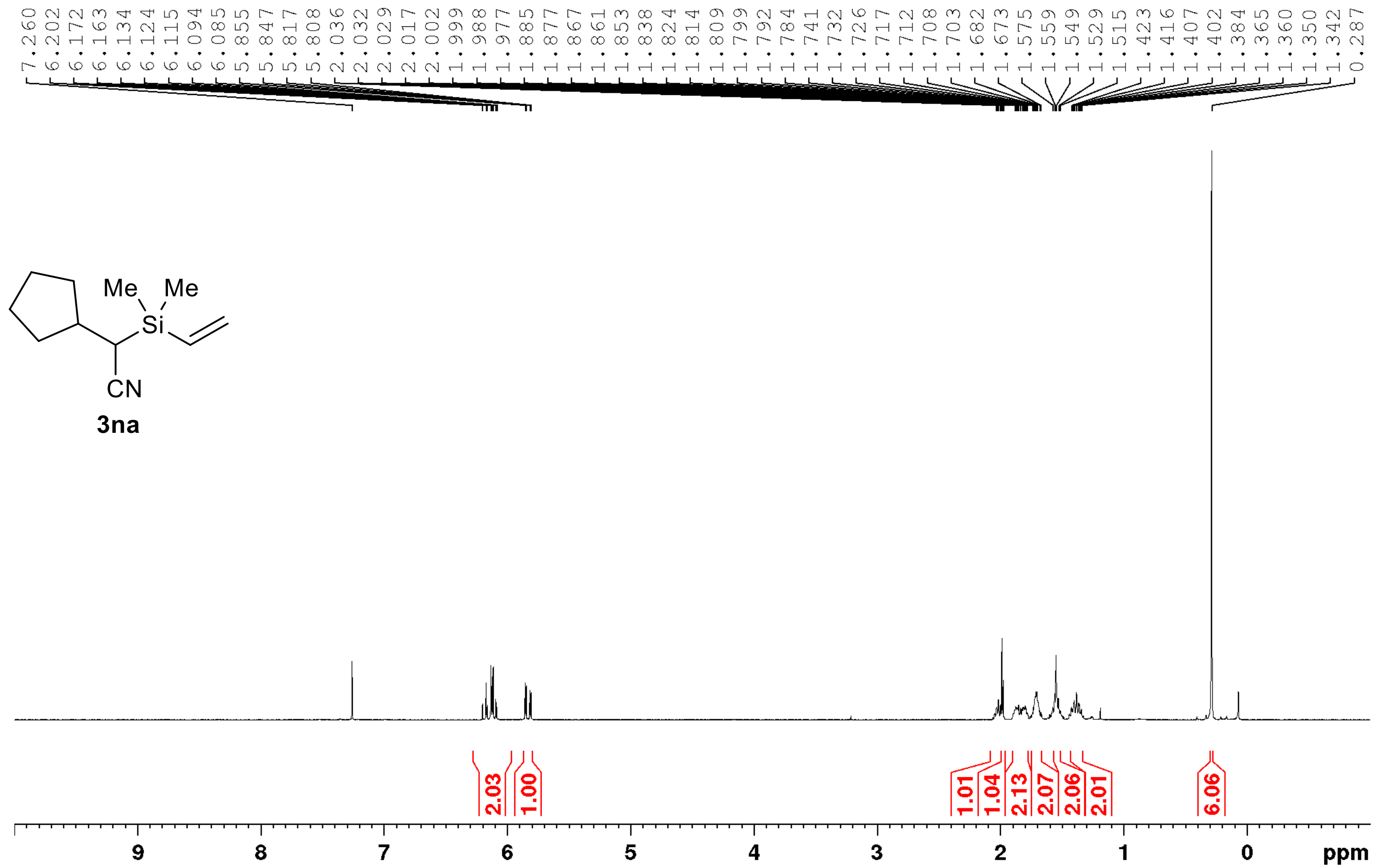

Figure S65. **$^{13}C\{^{1}H\}$ NMR** (125 MHz, $CDCl_3$, 298K) of **2-Cyclopentyl-2-(dimethyl(vinyl)silyl)acetonitrile (3na)**

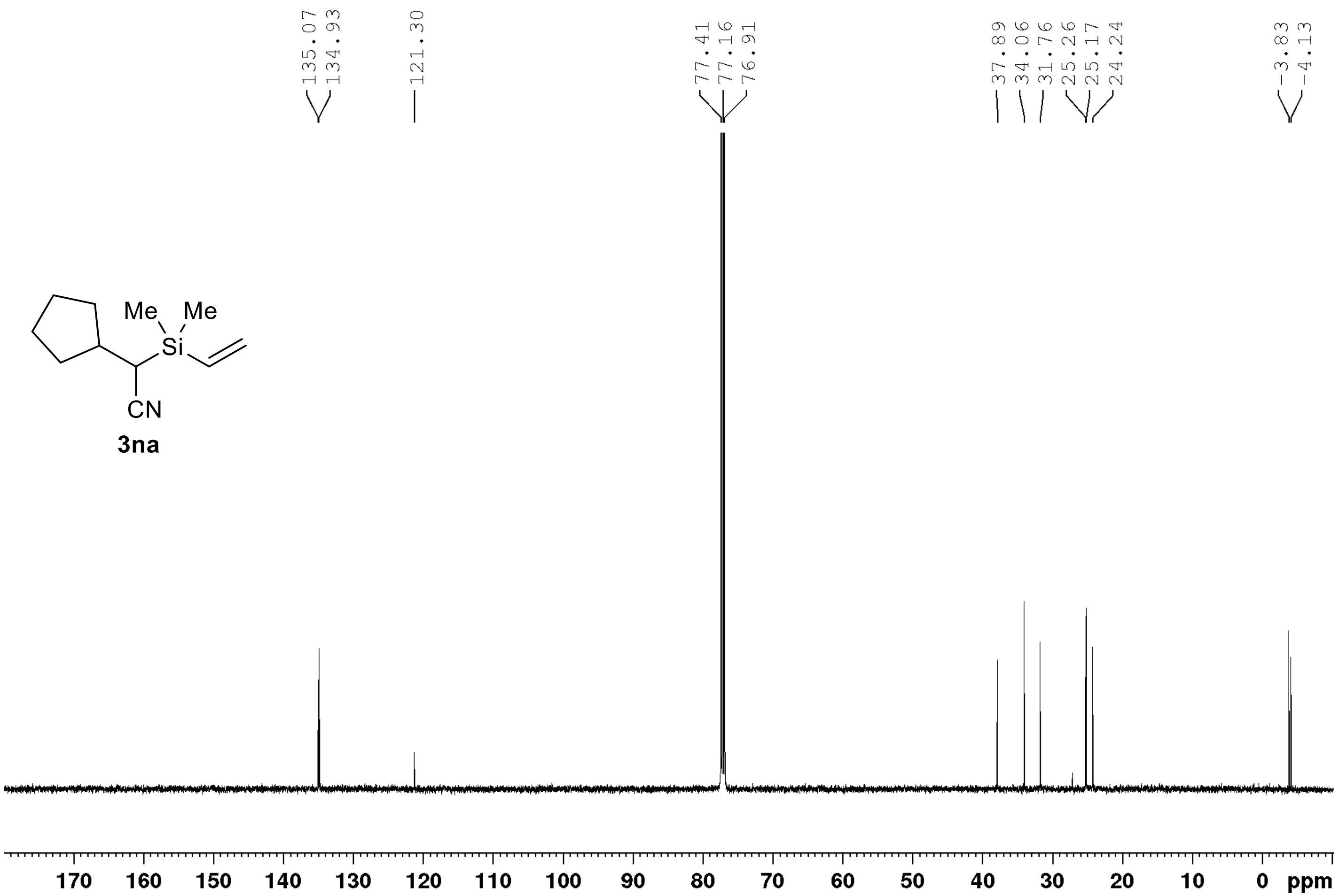

Figure S66. $^{1}H/^{29}Si$ HMQC NMR (500/99 MHz, $CDCl_3$, optimized for J = 7.0 Hz) of **2-Cyclopentyl-2-(dimethyl(vinyl)silyl)acetonitrile (3na)**

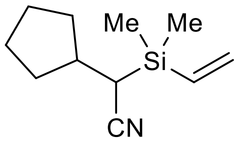

3na

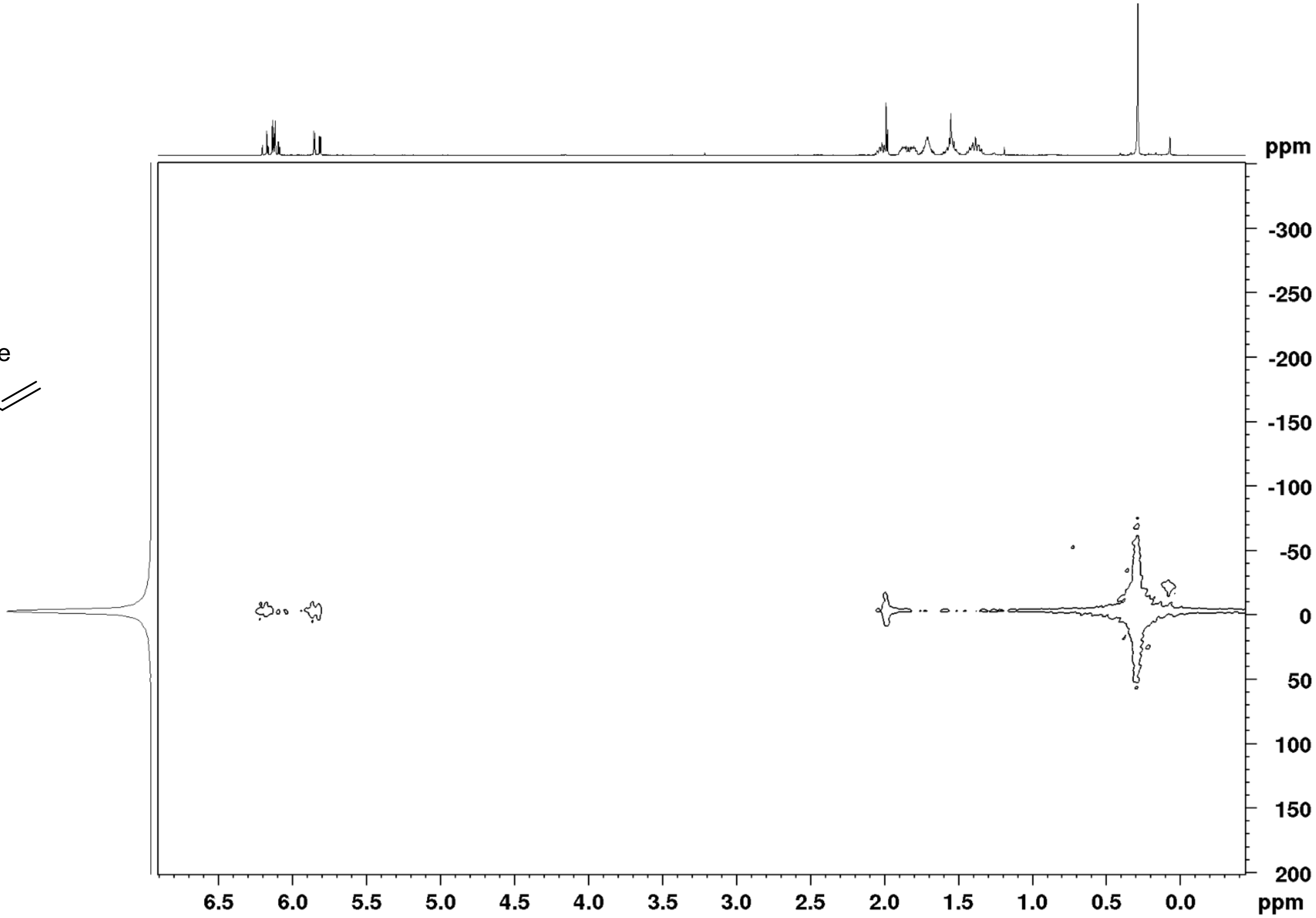

Figure S67. ^{1}H NMR (500 MHz, $CDCl_3$, 298K) of **2-Cyclohexyl-2-(dimethyl(vinyl)silyl)acetonitrile (3oa)**

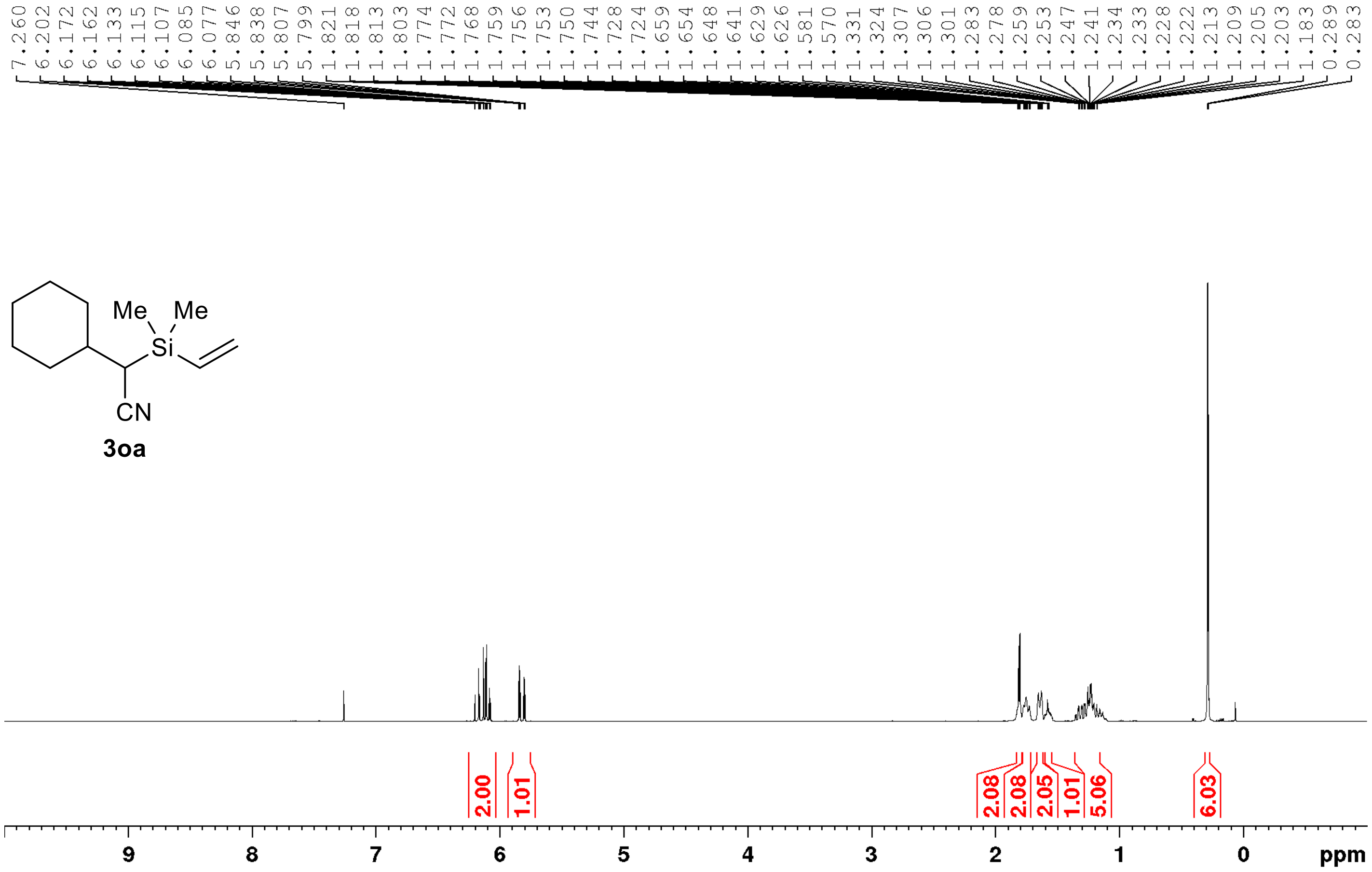

Figure S68. $^{13}C\{^{1}H\}$ **NMR** (125 MHz, $CDCl_3$, 298K) of **2-Cyclohexyl-2-(dimethyl(vinyl)silyl)acetonitrile (3oa)**

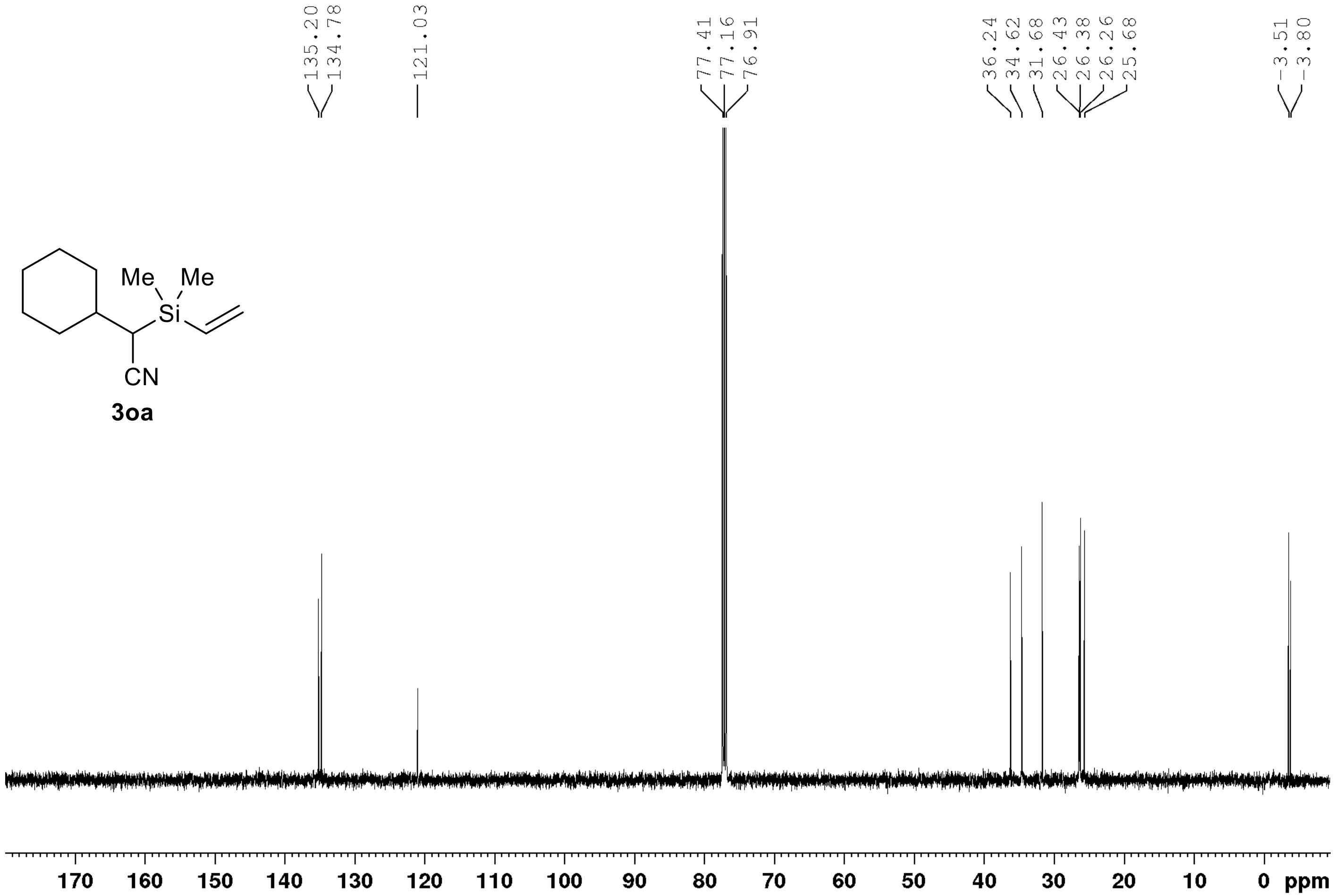

Figure S69. ^{29}**Si{^{1}H} DEPT NMR** (99 MHz, $CDCl_3$) of **2-Cyclohexyl-2-(dimethyl(vinyl)silyl)acetonitrile (3oa)**

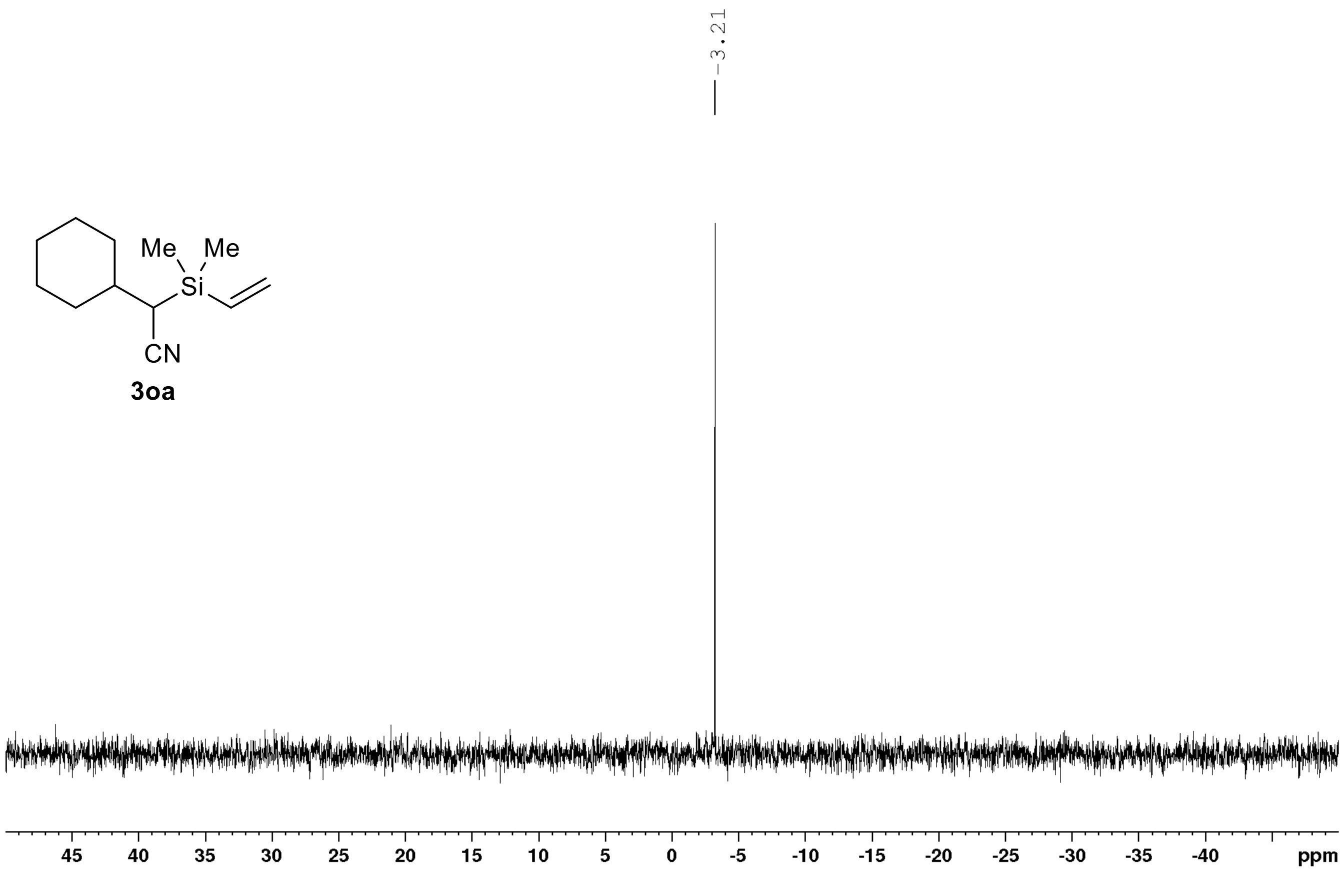

Figure S70. ^{1}H NMR (500 MHz, $CDCl_3$, 298K) of **2-(Dimethyl(vinyl)silyl)-2-(tetrahydro-2*H*-pyran-4-yl)acetonitrile (3pa)**

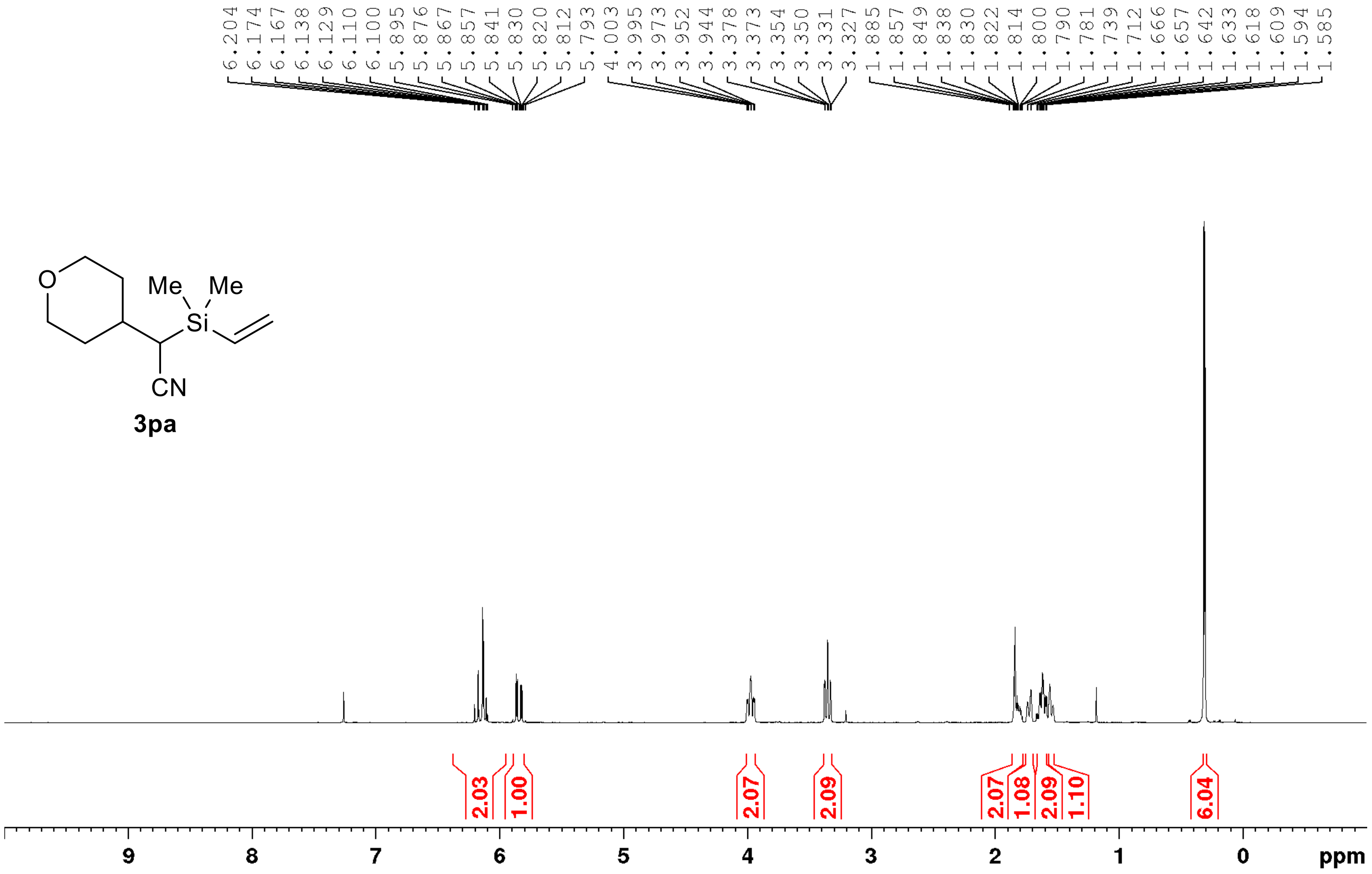

3pa

Figure S71. $^{13}C\{^{1}H\}$ **NMR** (125 MHz, $CDCl_3$, 298K) of **2-(Dimethyl(vinyl)silyl)-2-(tetrahydro-2*H*-pyran-4-yl)acetonitrile (3pa)**

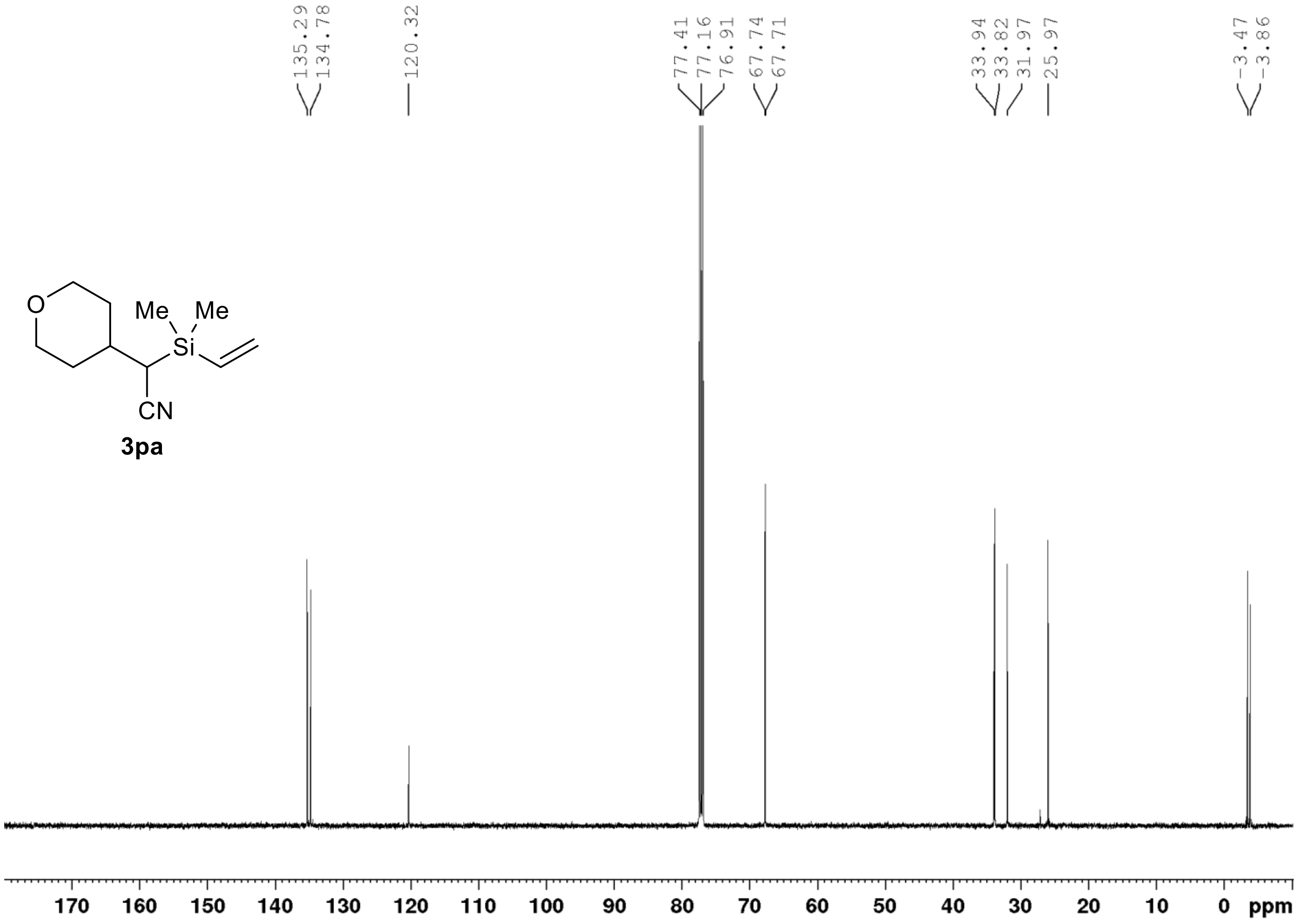

Figure S72. ^{29}Si{^{1}H} DEPT NMR (99 MHz, $CDCl_3$) of **2-(Dimethyl(vinyl)silyl)-2-(tetrahydro-2*H*-pyran-4-yl)acetonitrile (3pa)**

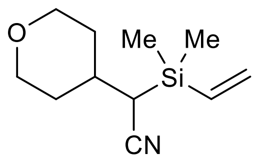

3pa

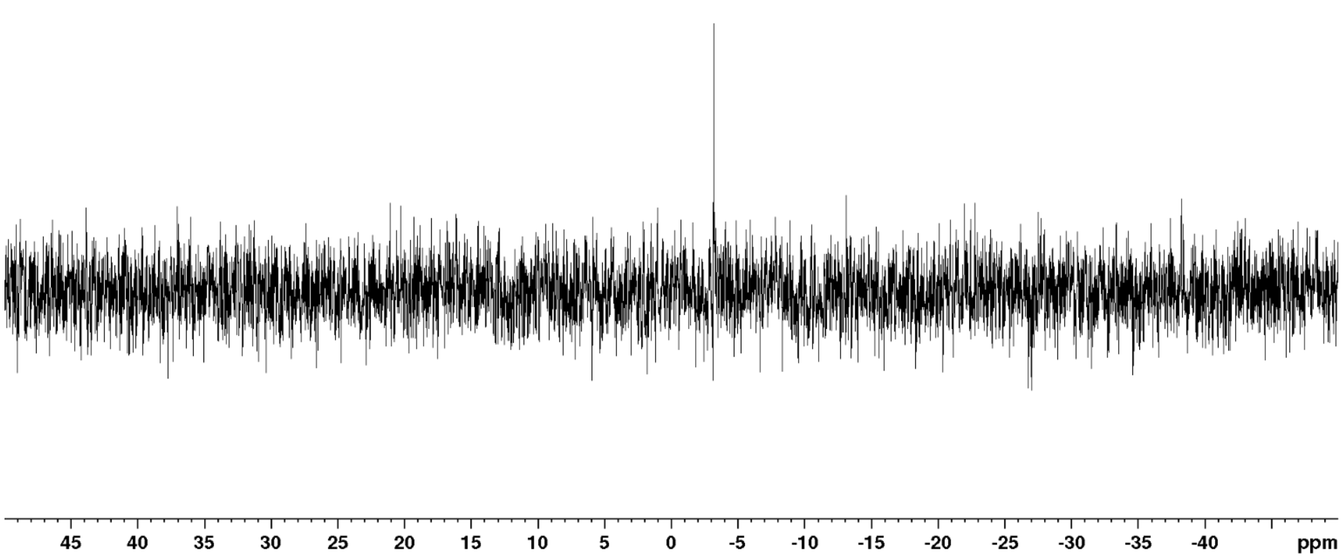

Figure S73. ^{1}H NMR (500 MHz, $CDCl_3$, 298K) of ***tert*-Butyl 4-(cyano(dimethyl(vinyl)silyl)methyl)piperidine-1-carboxylate (3qa)** (with small amounts of unknown impurities in the aliphatic region)

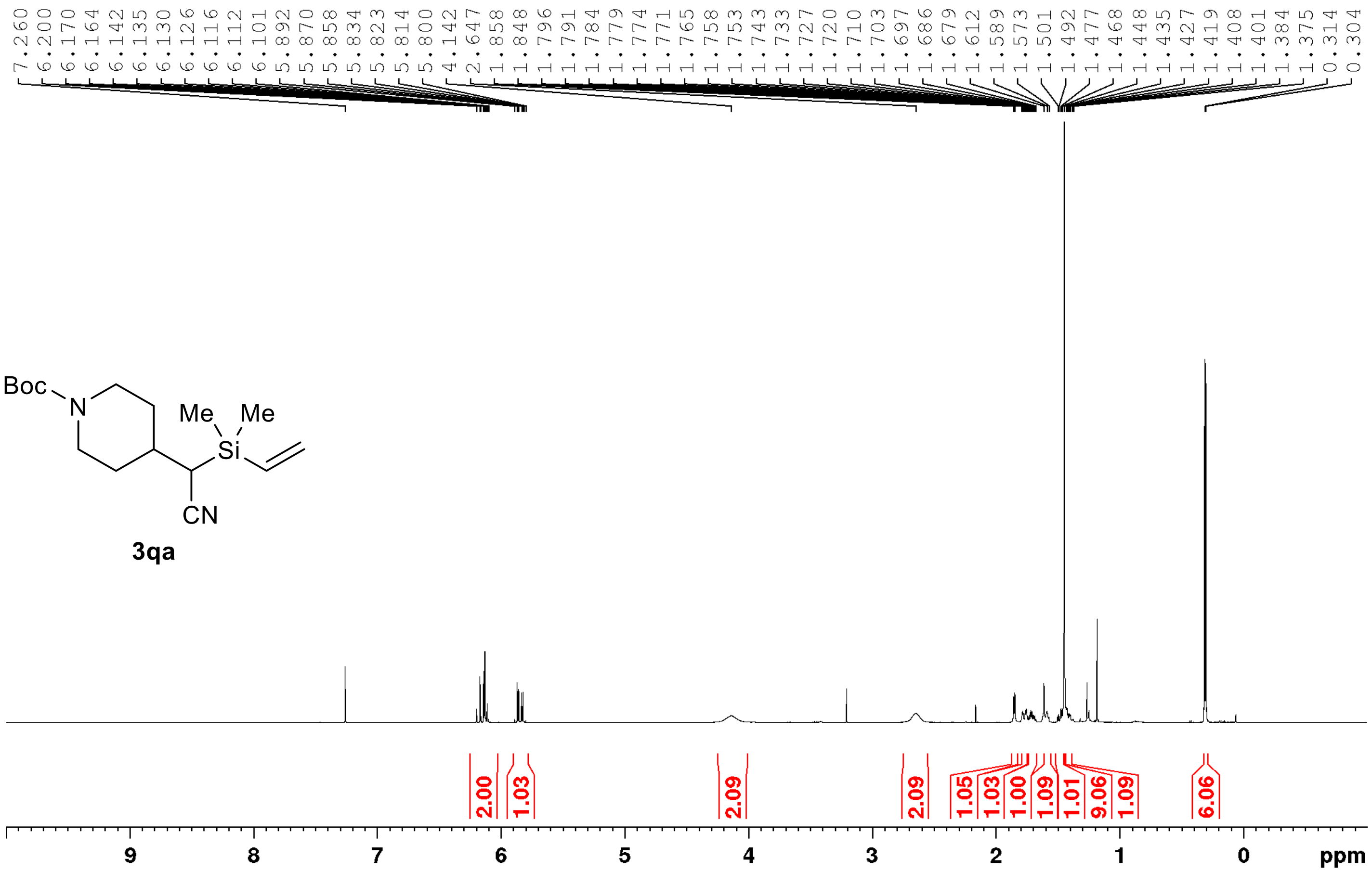

Figure S74. $^{13}C\{^{1}H\}$ **NMR** (125 MHz, $CDCl_3$, 298K) of ***tert*-Butyl 4-(cyano(dimethyl(vinyl)silyl)methyl)piperidine-1-carboxylate (3qa)** (with small amounts of unknown impurities in the aliphatic region)

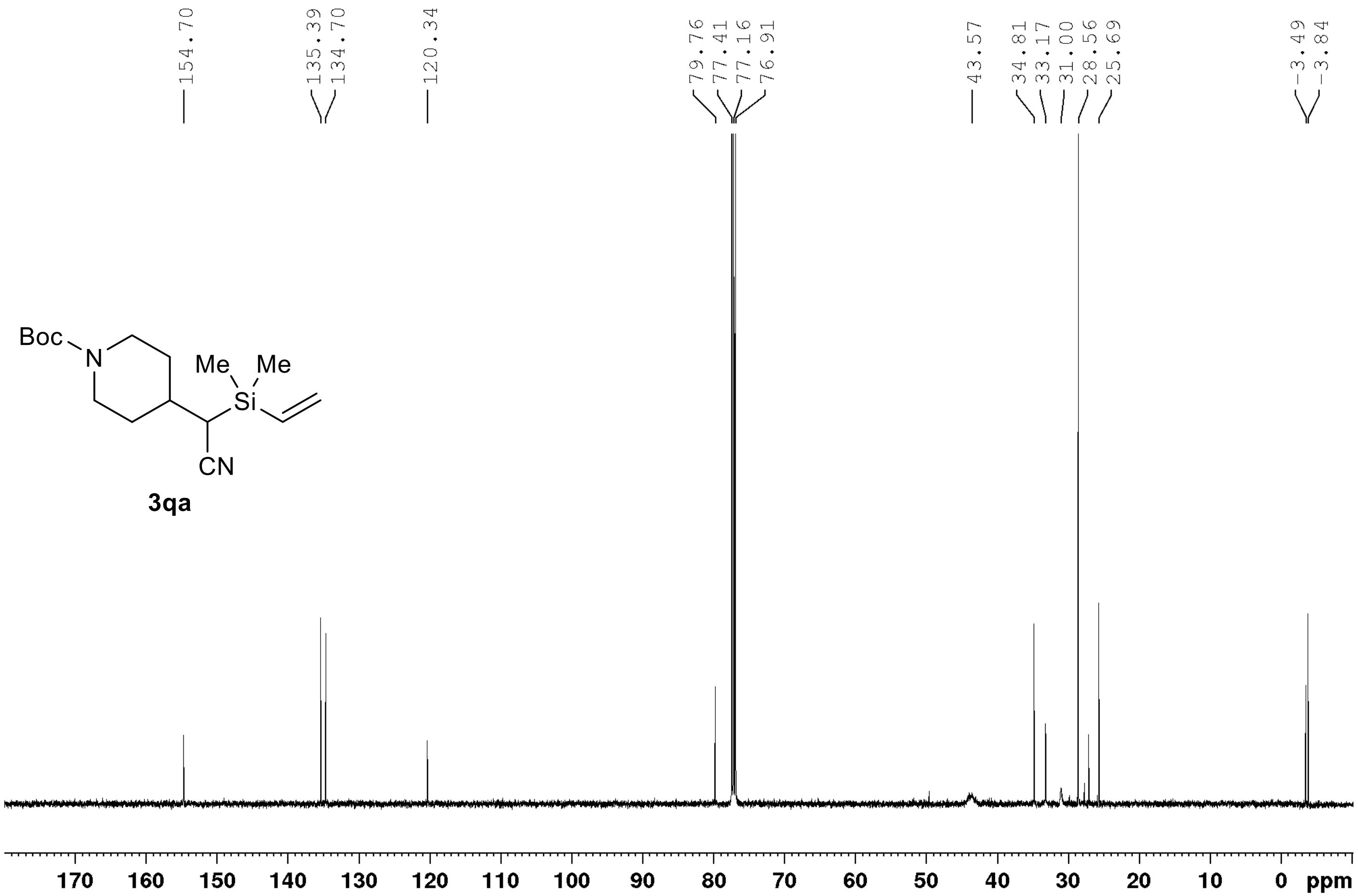

Figure S75. ^{29}Si{^{1}H} DEPT NMR (99 MHz, $CDCl_3$) of ***tert*-Butyl 4-(cyano(dimethyl(vinyl)silyl)methyl)piperidine-1-carboxylate (3qa)**

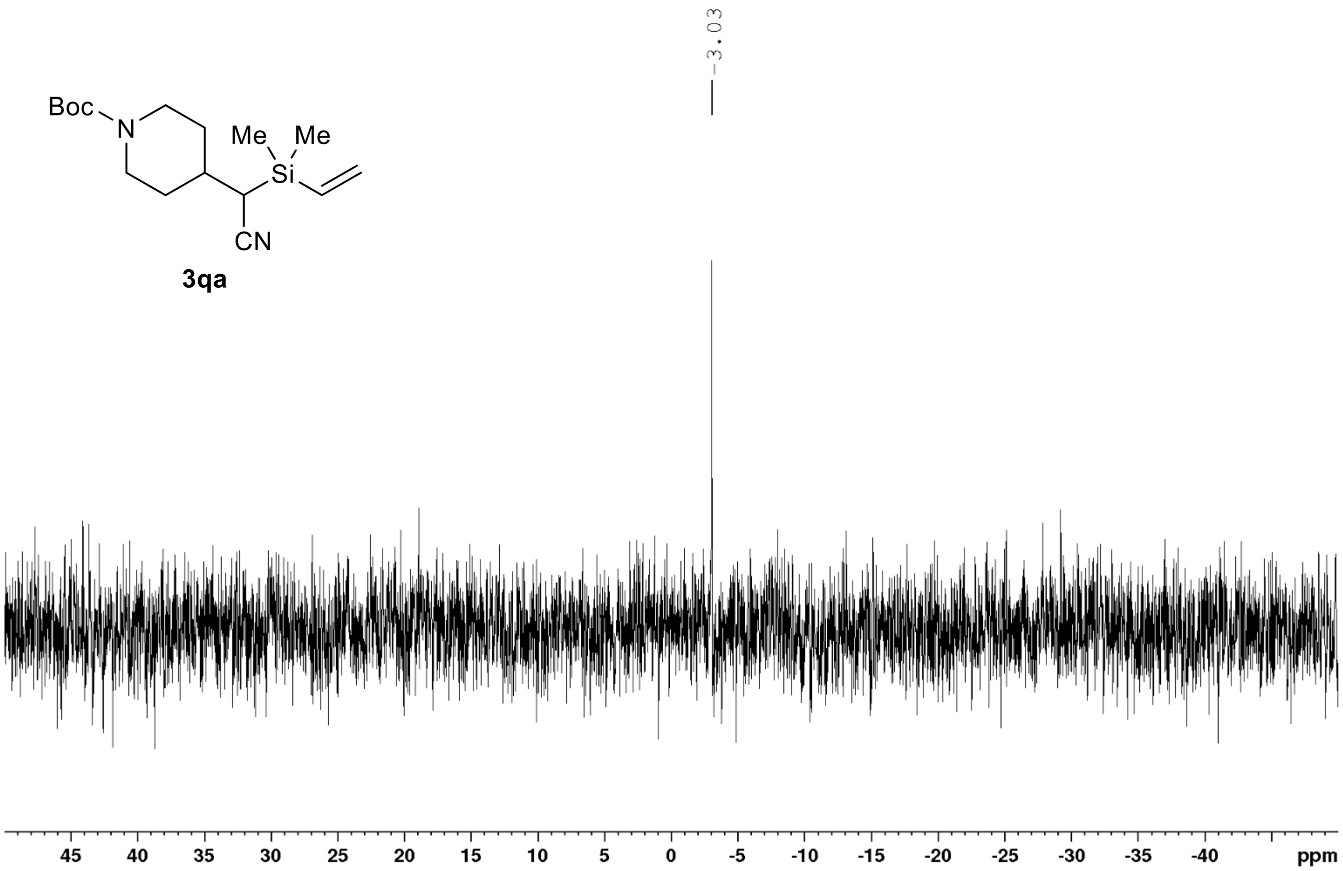

Figure S76. ^{1}**H NMR** (500 MHz, $CDCl_3$, 298K) of **2-(Dimethyl(vinyl)silyl)-3,3-dimethylbutanenitrile (3ra)**

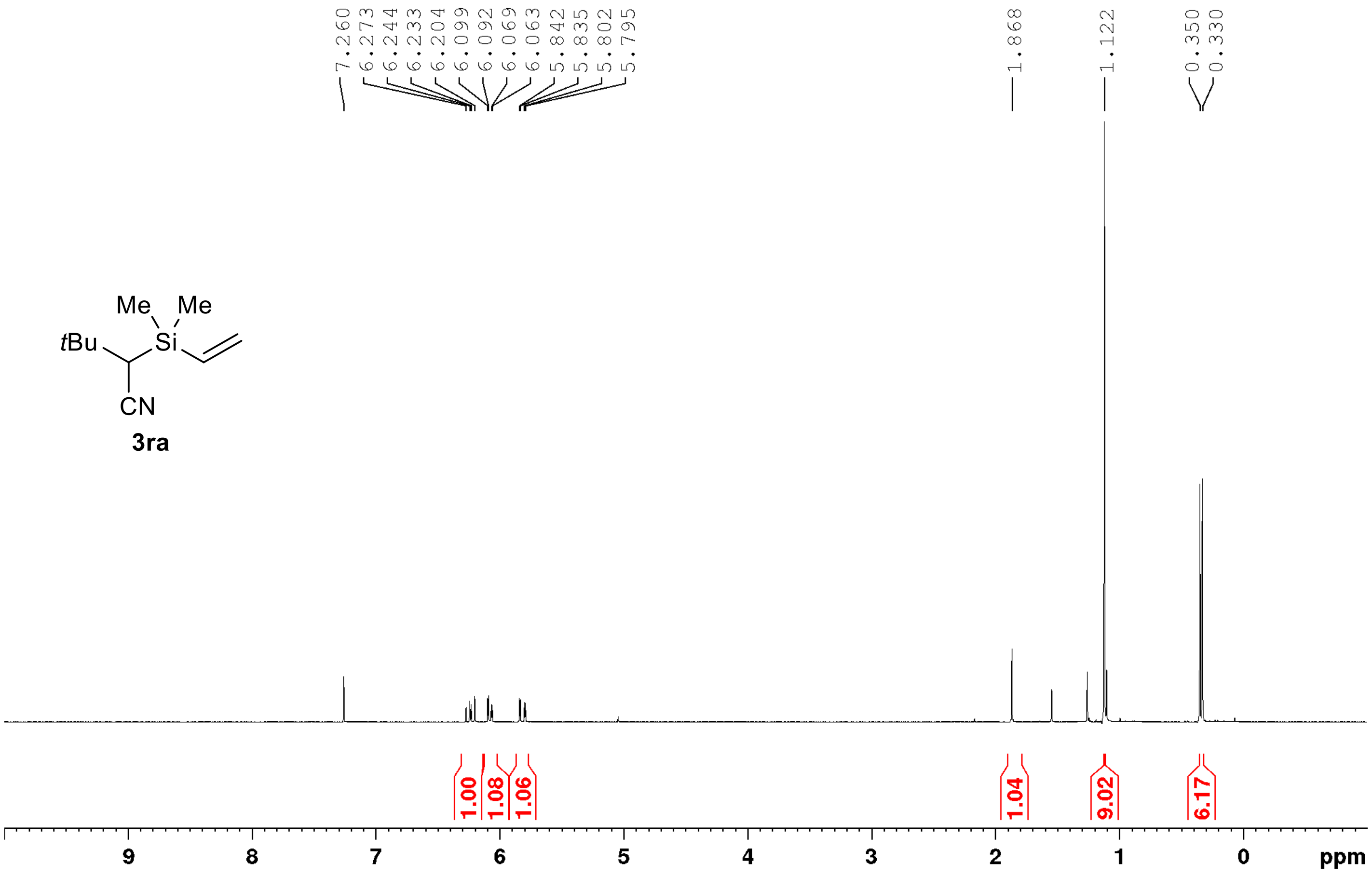

Figure S77. $^{13}C\{^1H\}$ **NMR** (125 MHz, $CDCl_3$, 298K) of **2-(Dimethyl(vinyl)silyl)-3,3-dimethylbutanenitrile (3ra)**

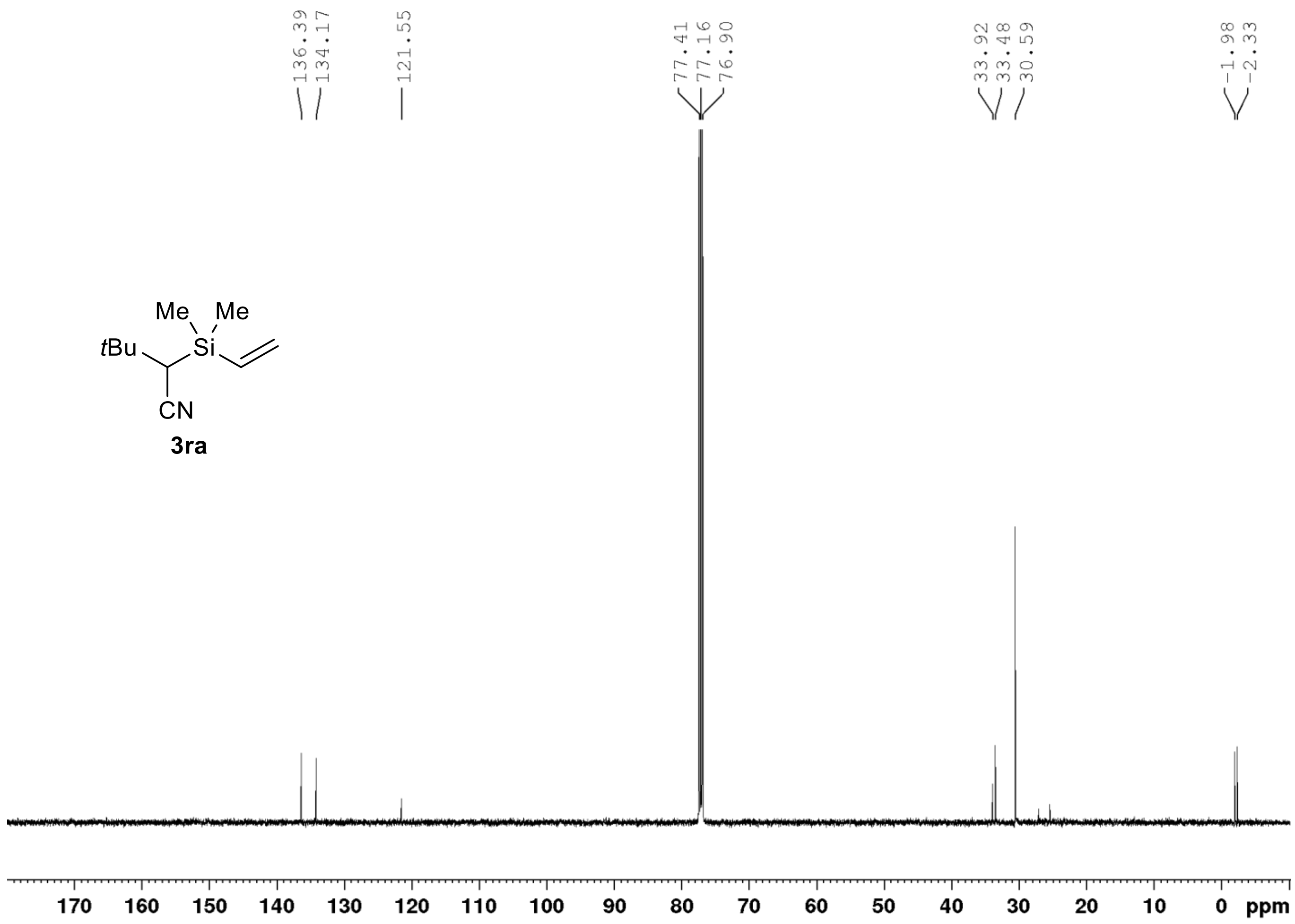

3ra

Figure S78. ^{1}H/^{29}Si **HMQC NMR** (500/99 MHz, $CDCl_3$, optimized for J = 7.0 Hz) of **2-(Dimethyl(vinyl)silyl)-3,3-dimethylbutanenitrile (3ra)**

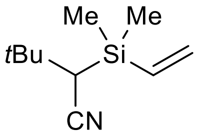

3ra

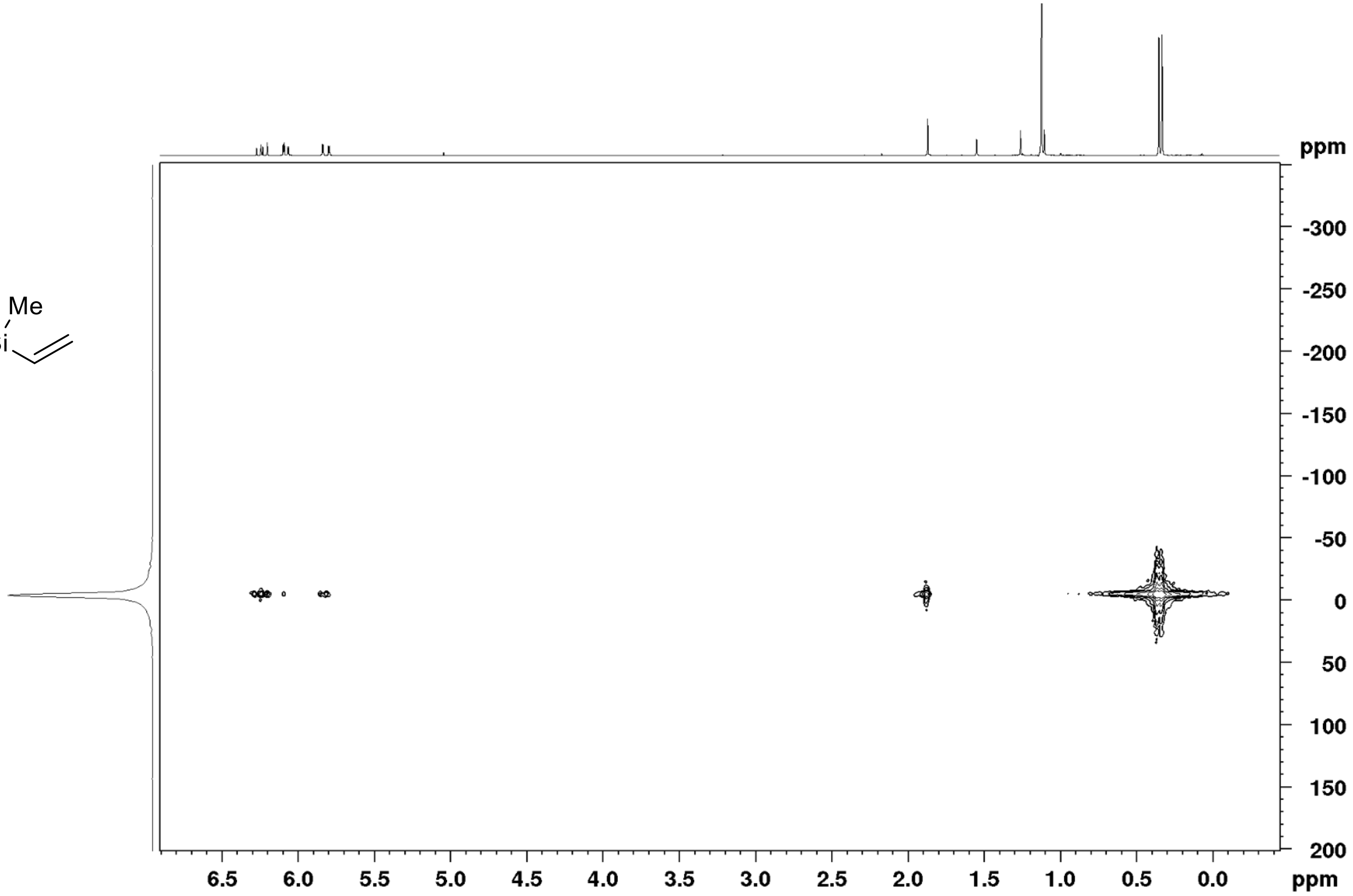

Figure S79. ^{1}H NMR (500 MHz, $CDCl_3$, 298K) of **2-Cyclopropyl-2-(dimethyl(vinyl)silyl)acetonitrile (3sa)**

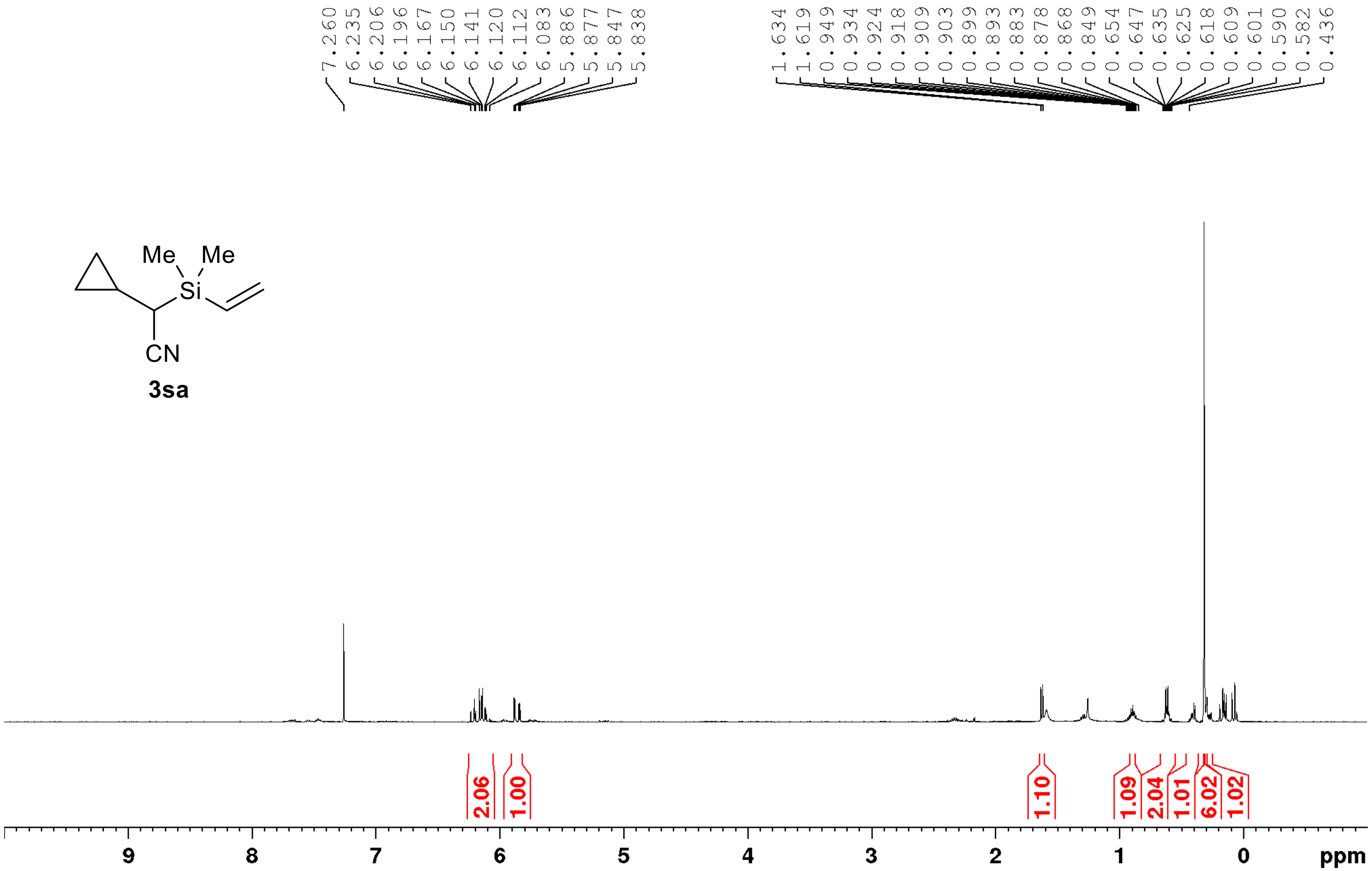

Figure S80. $^{13}C\{^1H\}$ NMR (125 MHz, $CDCl_3$, 298K) of **2-Cyclopropyl-2-(dimethyl(vinyl)silyl)acetonitrile (3sa)**

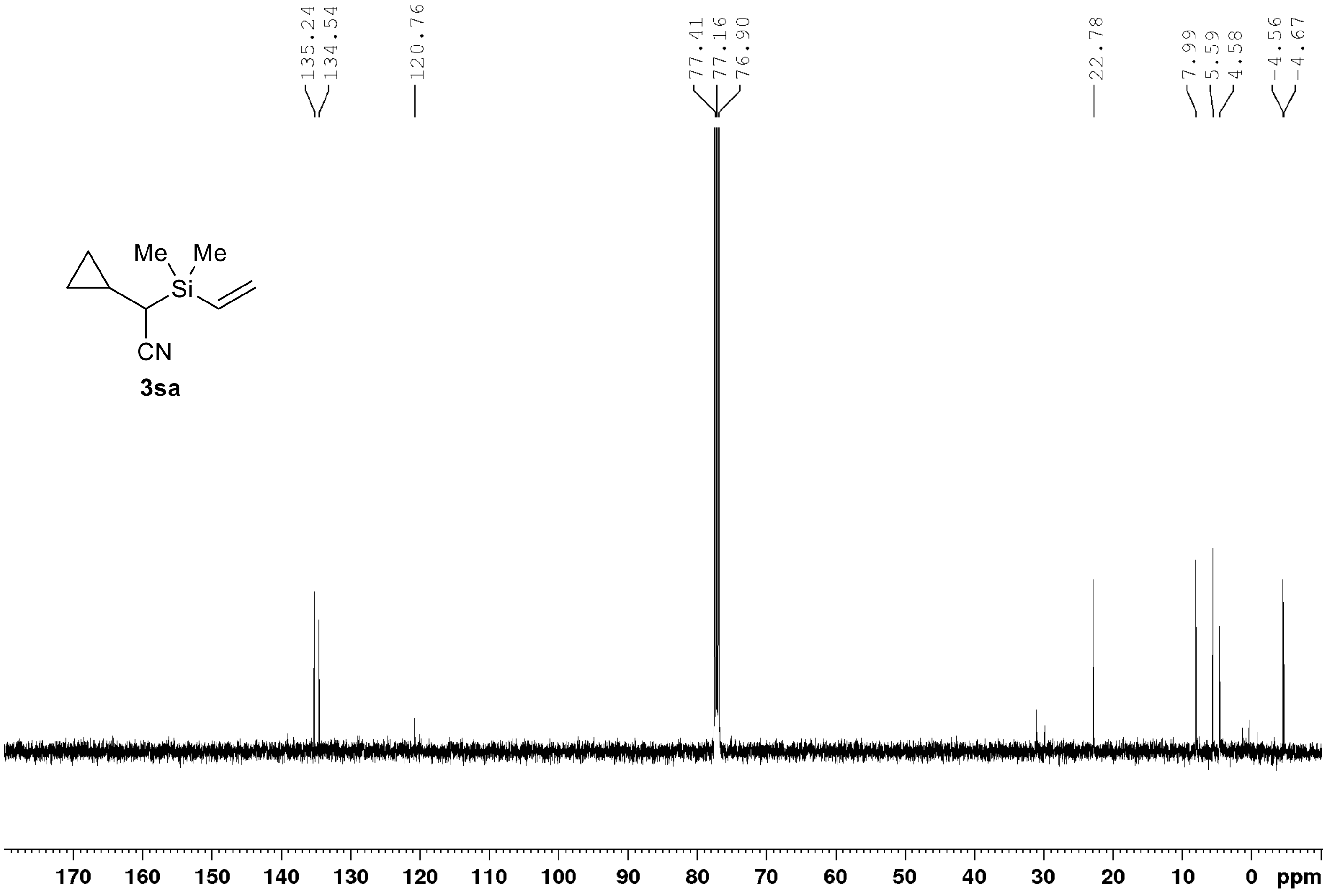

Figure S81. $^{1}H/^{29}Si$ HMQC NMR (500/99 MHz, $CDCl_3$, optimized for J = 7.0 Hz) of **2-Cyclopropyl-2-(dimethyl(vinyl)silyl)acetonitrile (3sa)**

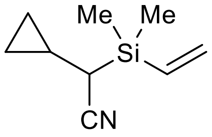

3sa

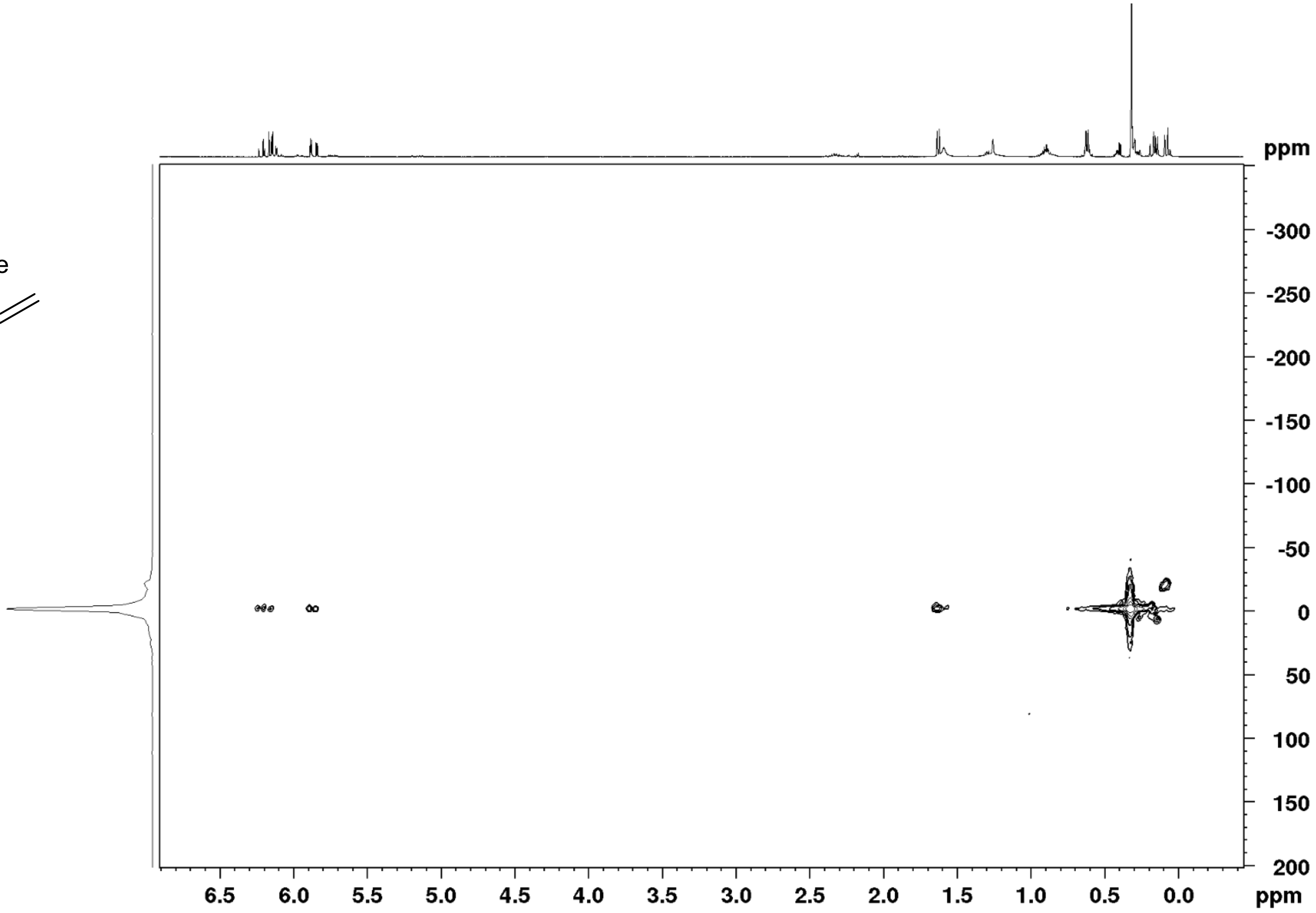

9. References

[S1] A. He, J. R. Falck, *J. Am. Chem. Soc.* **2010**, *132*, 2524–2525.

[S2] H. Dang, M. Mailig, G. Lalic, *Angew. Chem. Int. Ed.* **2014**, *53*, 6473–6476.

[S3] J. Scharfbier, H. Hazrati, E. Irran, M. Oestreich, *Org. Lett.* **2017**, *19*, 6562–6565.

[S4] S. Liu, X. Zeng, G. B. Hammond, B. Xu, *Adv. Synth. Catal.* **2018**, *360*, 3667–3671.

[S5] K. W. Shimkin, P. G. Gildner, D. A. Watson, *Org. Lett.* **2016**, *18*, 988−991.

[S6] A. S. Donslund, K. T. Neumann, N. P. Corneliussen, E. K. Grove, D. Herbstritt, K. Daasbjerg, T. Skrydstrup, *Chem. Eur. J.* **2019**, *25*, 9856–9860.

[S7] N. T. Kadunce, S. E. Reisman, *J. Am. Chem. Soc.* **2015**, *137*, 10480−10483.
